# Supplementary material for: TUT1-catalyzed U6 snRNA 3′-end maturation is essential for RNA splicing and stem cell survival
Source: EMBO Rep. 2026 Apr 9;27(10):2703–30. doi: 10.1038/s44319-026-00759-8 (PMC13219678; doi:10.1038/s44319-026-00759-8)
Supplement: Supplementary file 2 — Movie EV1 [file 44319_2026_759_MOESM2_ESM.zip › Movie EV1/Movie EV1.docx]

**Movie EV1. P14 littermates of wild-type and *Tut1^f/f^*;Emx1-Cre mice.**
