# Supplementary figures and images for: TUT1-catalyzed U6 snRNA 3′-end maturation is essential for RNA splicing and stem cell survival
Source: EMBO Rep. 2026 Apr 9;27(10):2703–30. doi: 10.1038/s44319-026-00759-8 (PMC13219678; doi:10.1038/s44319-026-00759-8)

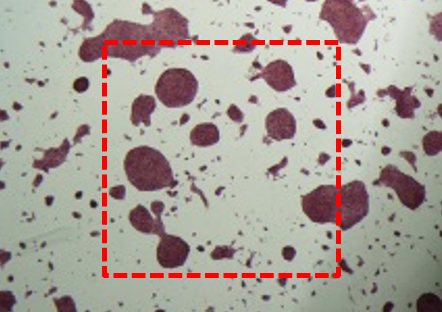

Supplement: Supplementary file 3 — Source data Fig. 1 [file 44319_2026_759_MOESM3_ESM.zip › Source Data for Figure 1/1E/Scr.tif]

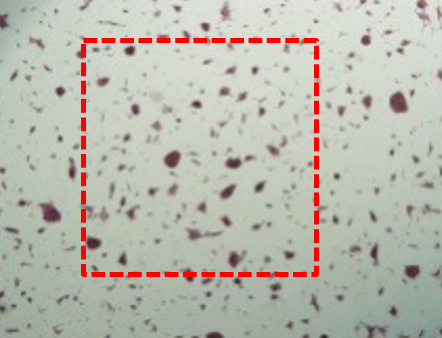

Supplement: Supplementary file 3 — Source data Fig. 1 [file 44319_2026_759_MOESM3_ESM.zip › Source Data for Figure 1/1E/T1.tif]

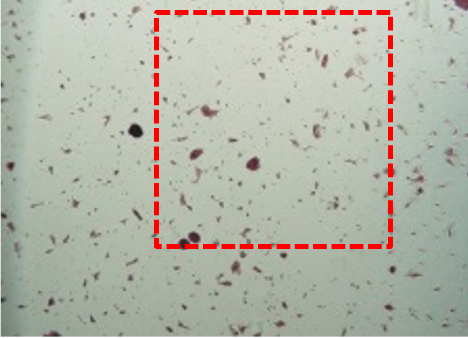

Supplement: Supplementary file 3 — Source data Fig. 1 [file 44319_2026_759_MOESM3_ESM.zip › Source Data for Figure 1/1E/T2.tif]

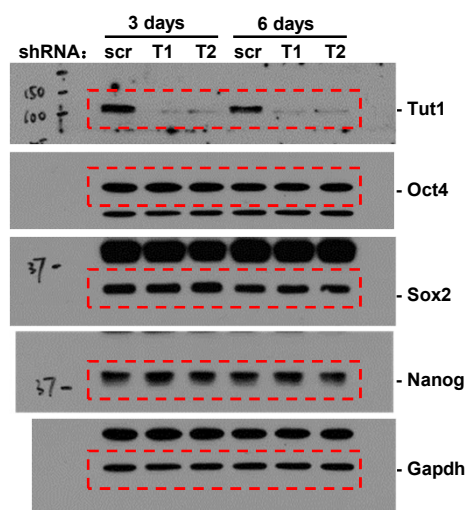

Supplement: Supplementary file 3 — Source data Fig. 1 [file 44319_2026_759_MOESM3_ESM.zip › Source Data for Figure 1/1F/western.pdf]

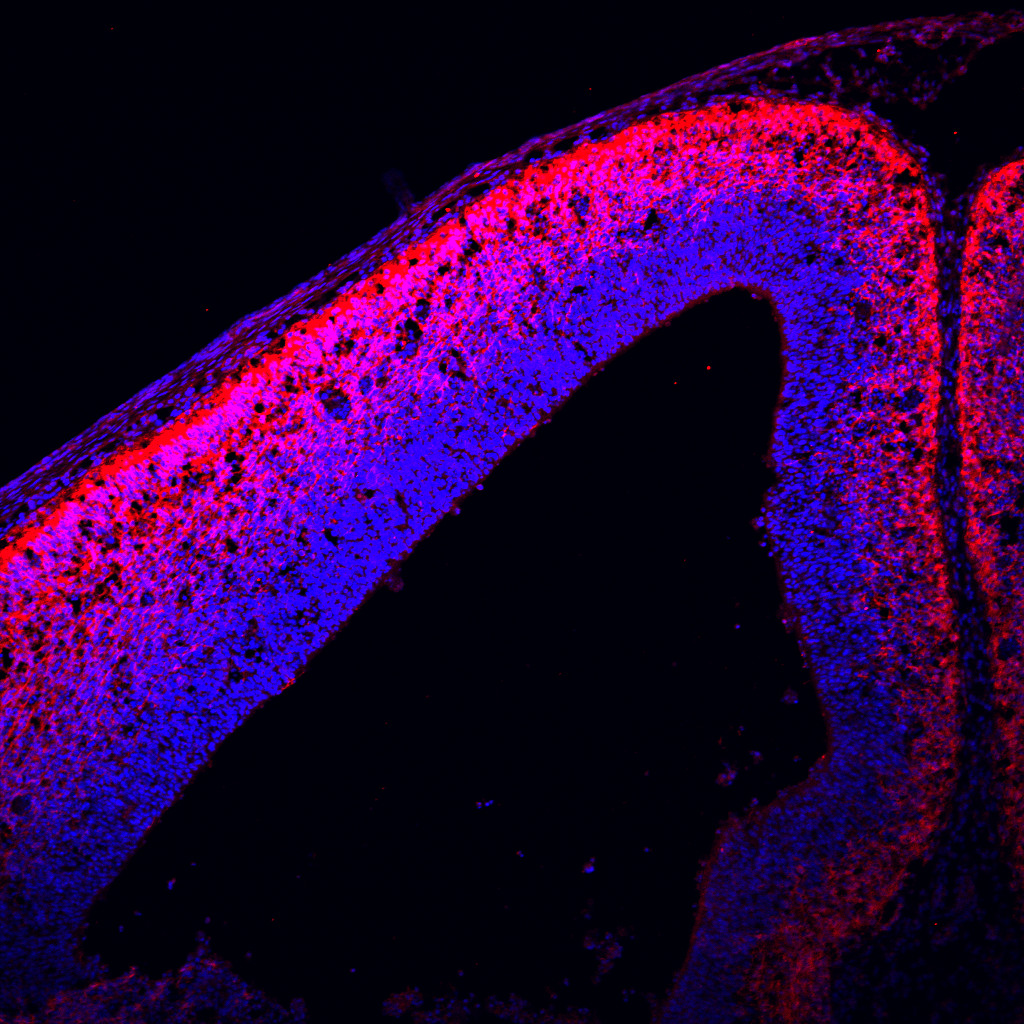

Supplement: Supplementary file 4 — Source data Fig. 2 [file 44319_2026_759_MOESM4_ESM.zip › Source Data for Figure 2/2E/TUT1 ff; nestin-cre-E14.5.tif]

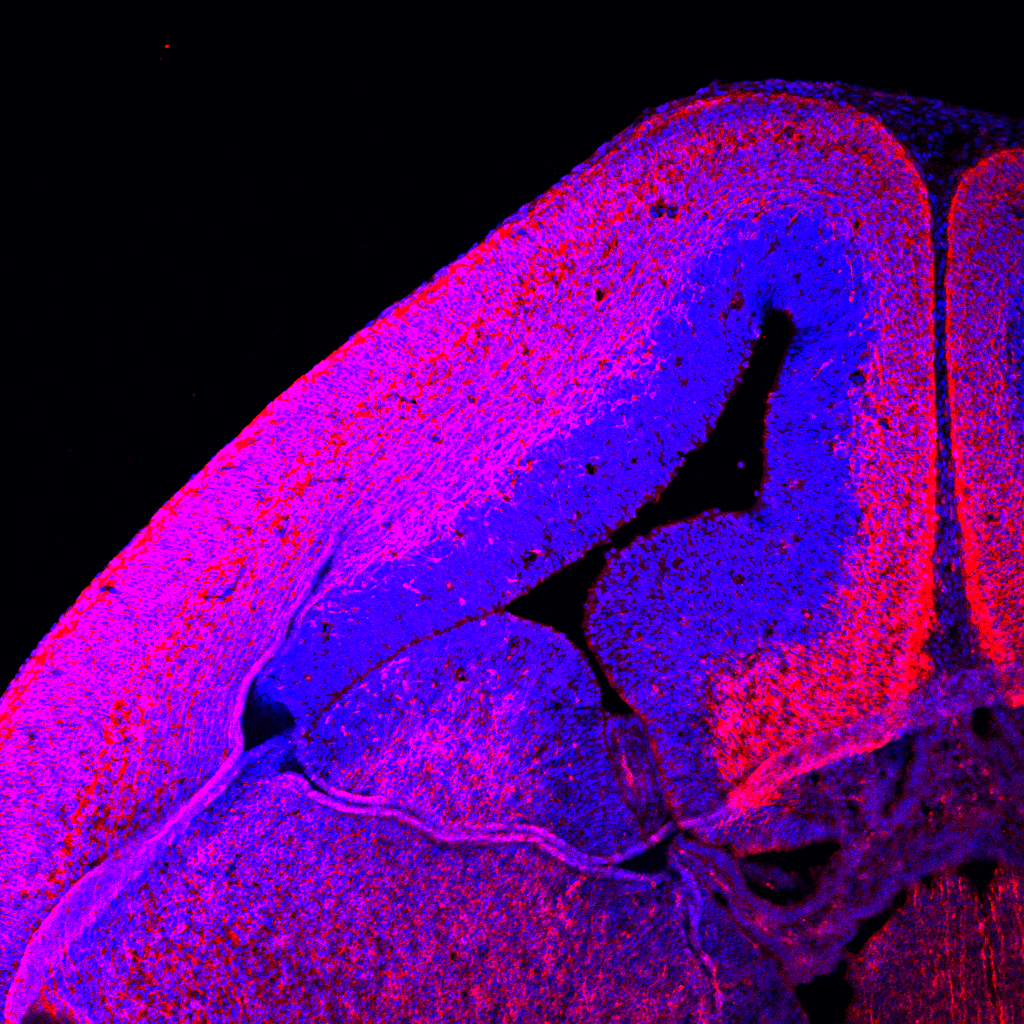

Supplement: Supplementary file 4 — Source data Fig. 2 [file 44319_2026_759_MOESM4_ESM.zip › Source Data for Figure 2/2E/WT-E14.5.tif]

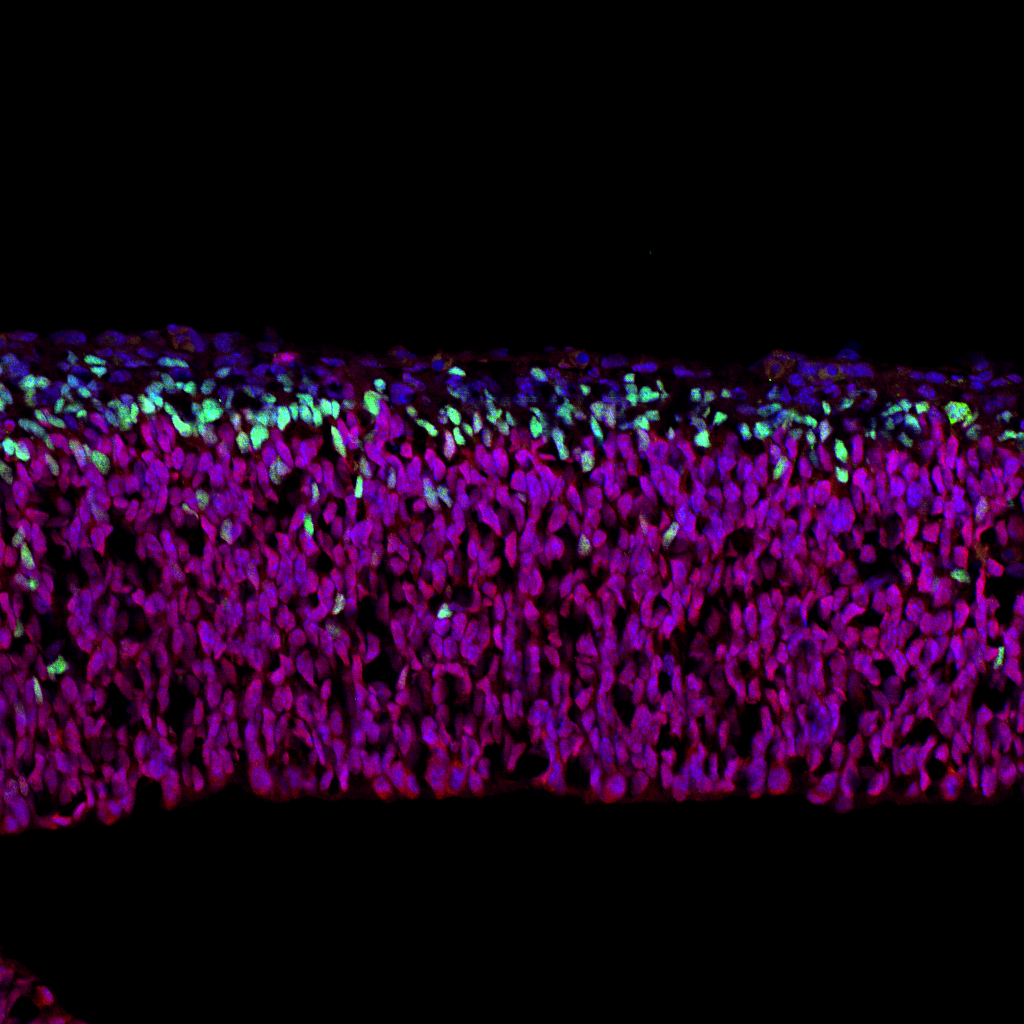

Supplement: Supplementary file 4 — Source data Fig. 2 [file 44319_2026_759_MOESM4_ESM.zip › Source Data for Figure 2/2F/E12.5-TUT1 ff; nestin-Cre.tif]

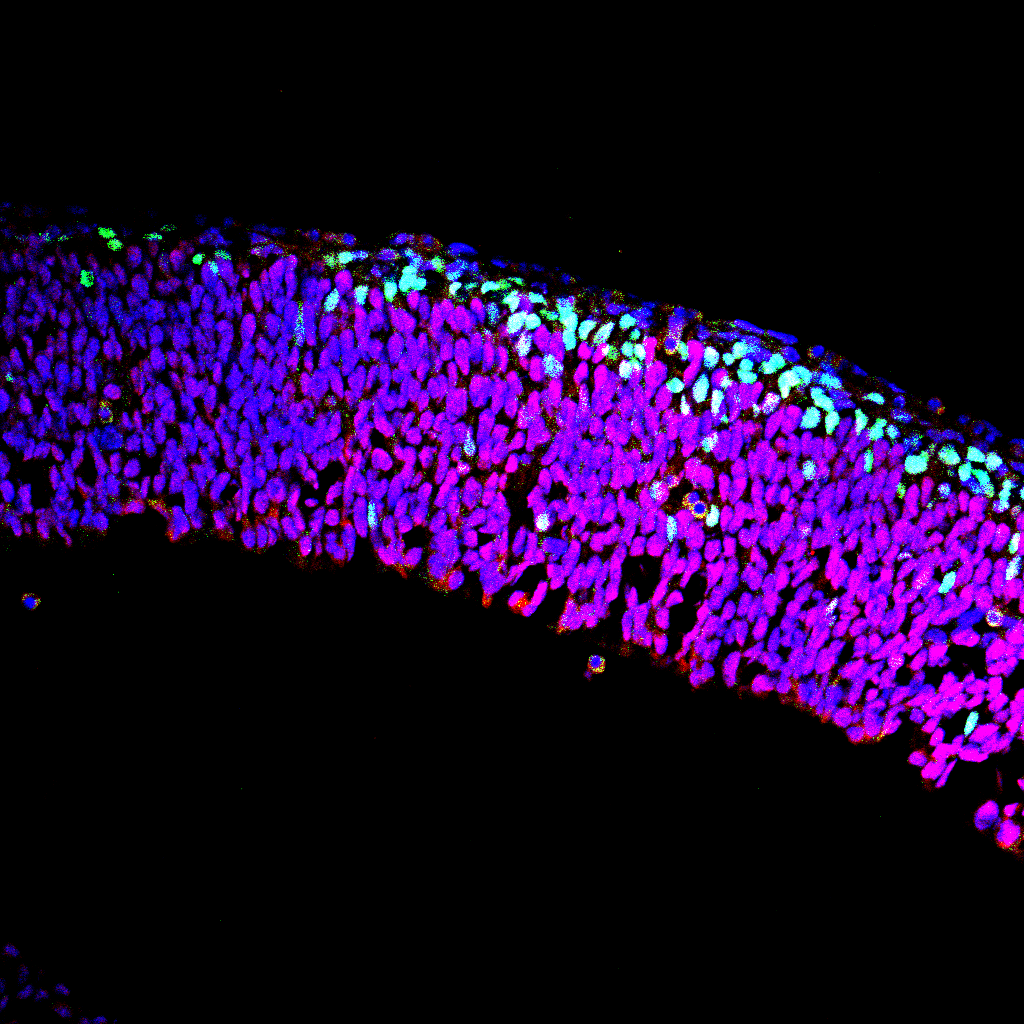

Supplement: Supplementary file 4 — Source data Fig. 2 [file 44319_2026_759_MOESM4_ESM.zip › Source Data for Figure 2/2F/E12.5-WT.tif]

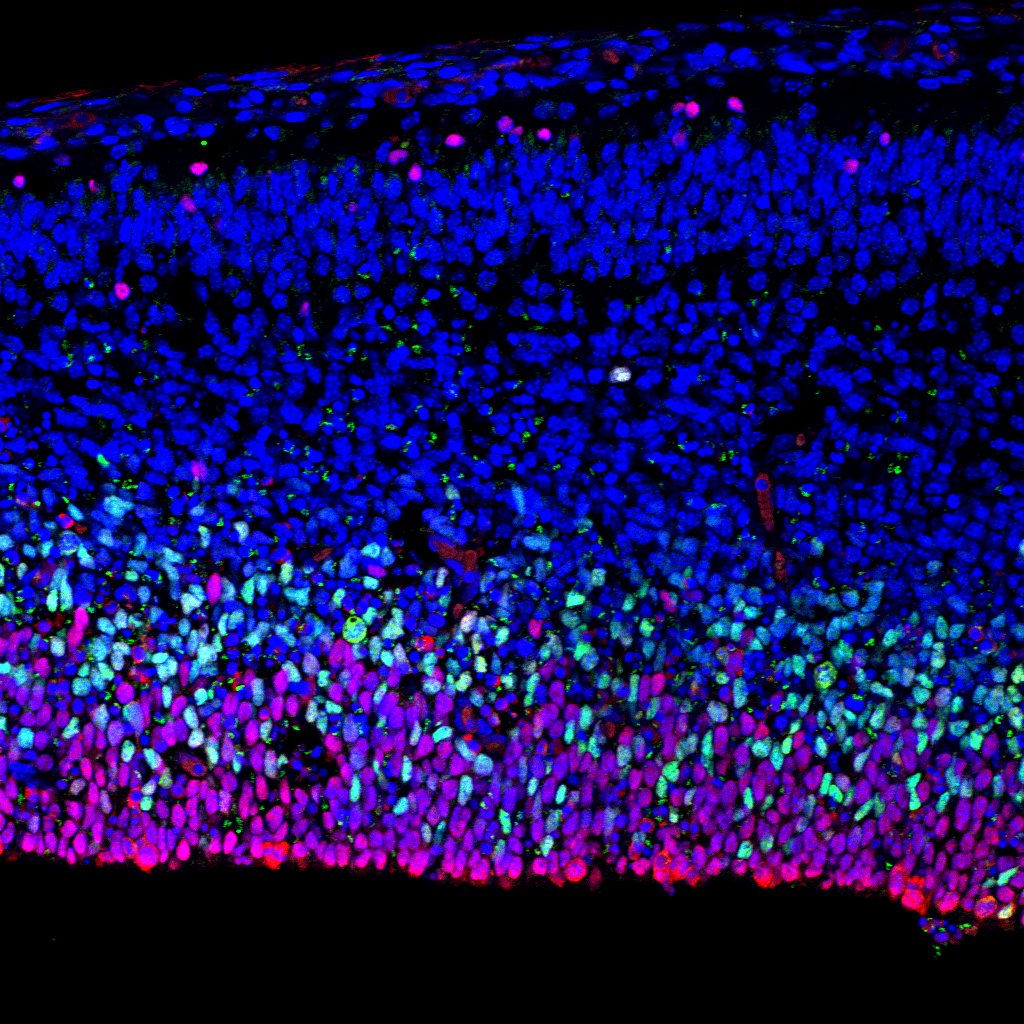

Supplement: Supplementary file 4 — Source data Fig. 2 [file 44319_2026_759_MOESM4_ESM.zip › Source Data for Figure 2/2F/E14.5-TUT1 ff; nestin-Cre.tif]

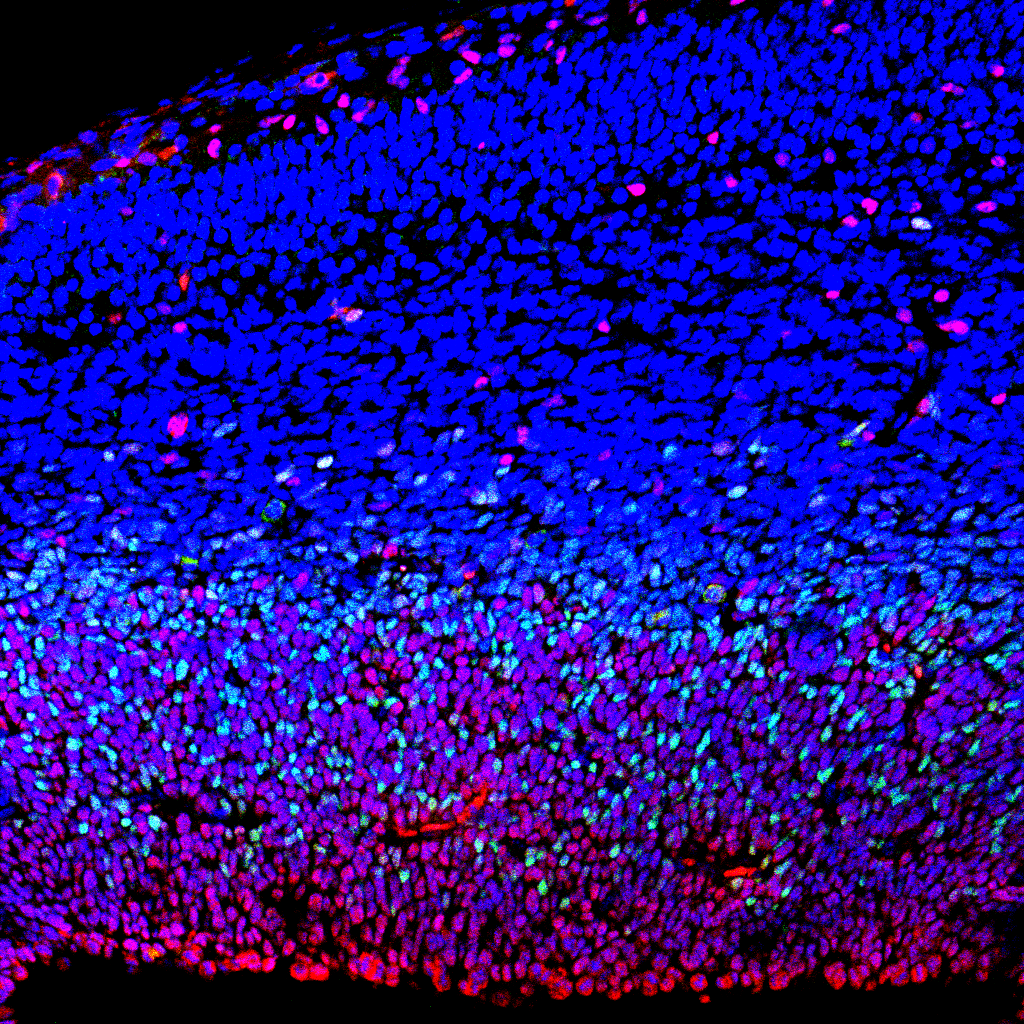

Supplement: Supplementary file 4 — Source data Fig. 2 [file 44319_2026_759_MOESM4_ESM.zip › Source Data for Figure 2/2F/E14.5-WT.tif]

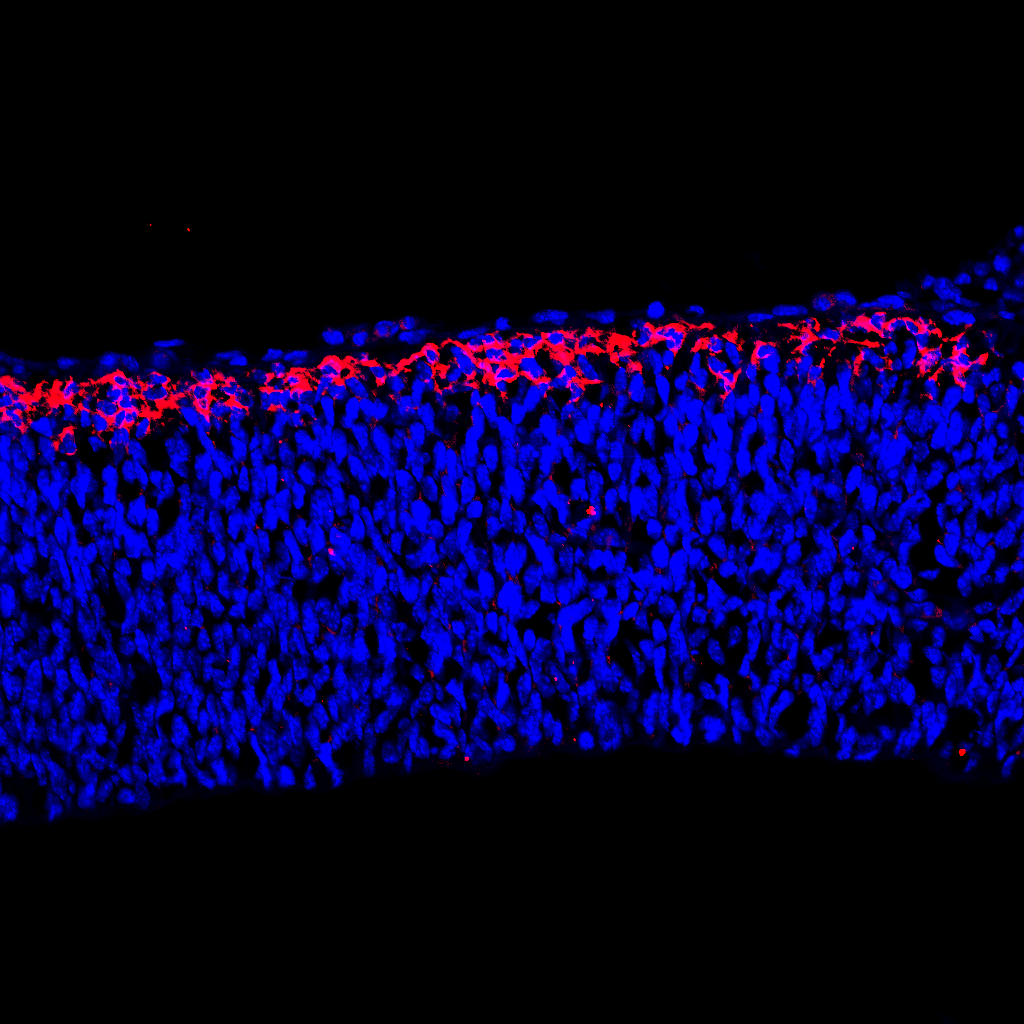

Supplement: Supplementary file 4 — Source data Fig. 2 [file 44319_2026_759_MOESM4_ESM.zip › Source Data for Figure 2/2G/E12.5-TUT1 ff; nestin-Cre.tif]

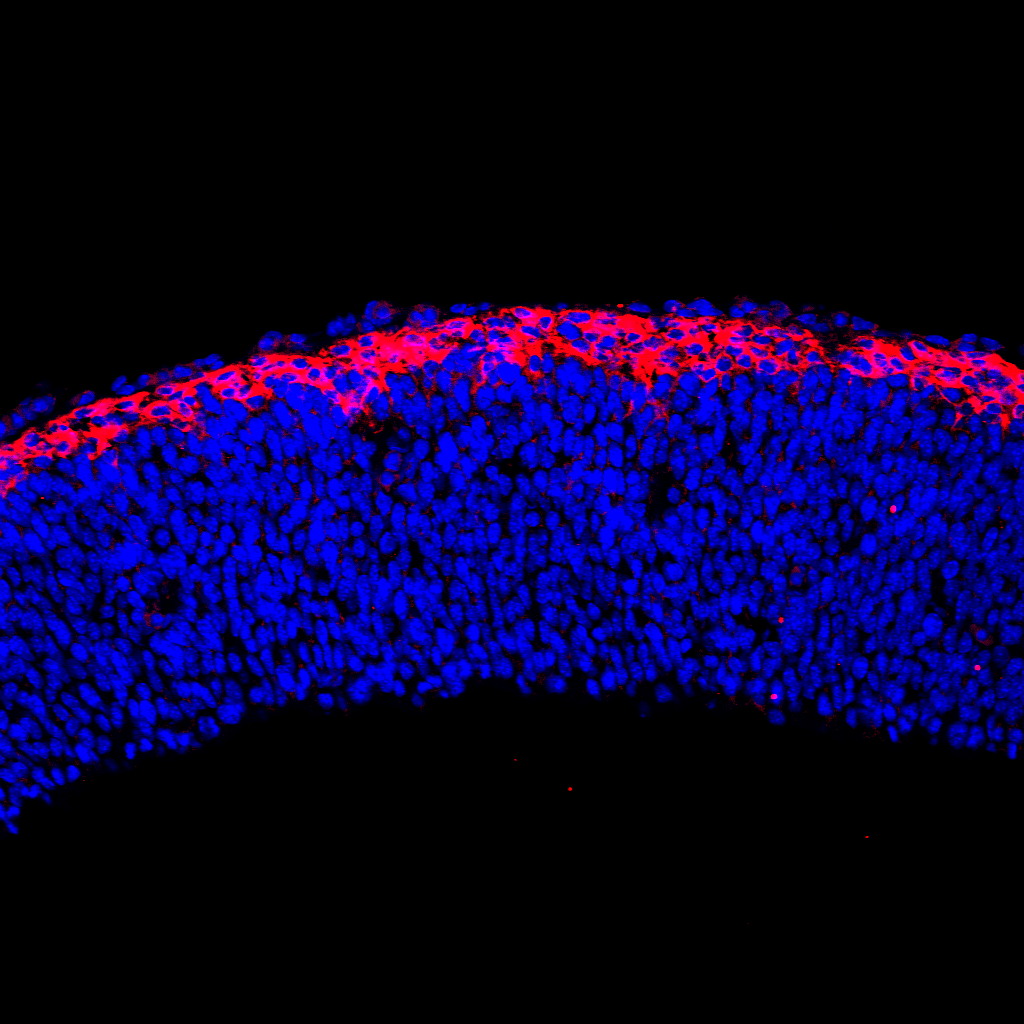

Supplement: Supplementary file 4 — Source data Fig. 2 [file 44319_2026_759_MOESM4_ESM.zip › Source Data for Figure 2/2G/E12.5-WT.tif]

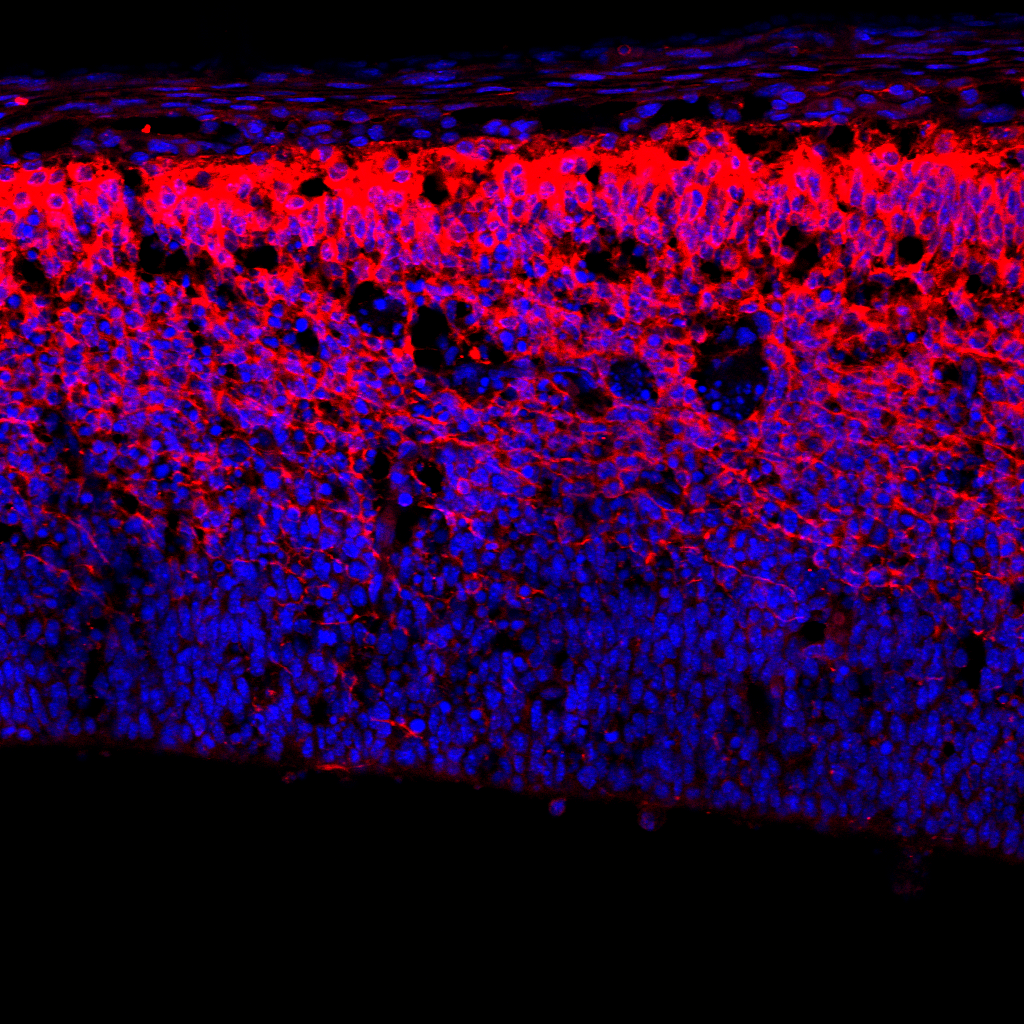

Supplement: Supplementary file 4 — Source data Fig. 2 [file 44319_2026_759_MOESM4_ESM.zip › Source Data for Figure 2/2G/E14.5-TUT1 ff; nestin-Cre.tif]

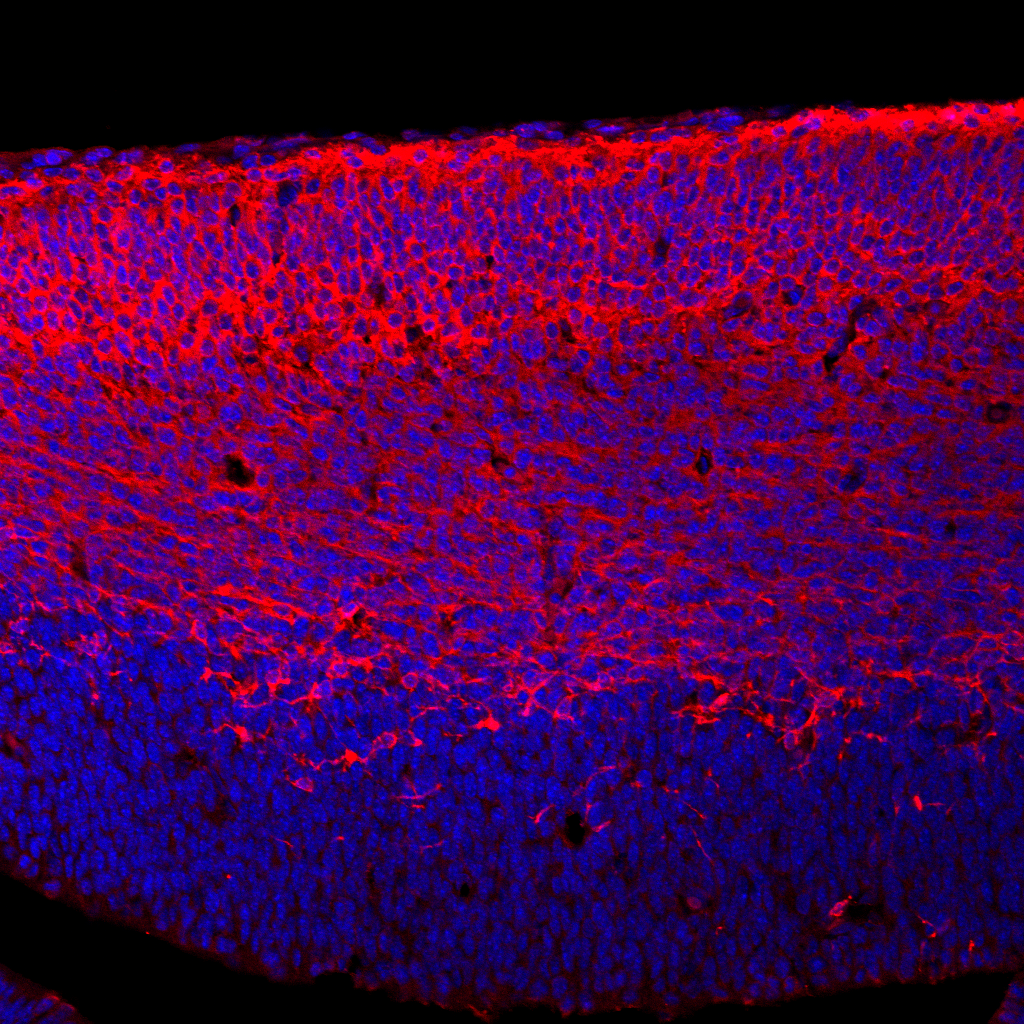

Supplement: Supplementary file 4 — Source data Fig. 2 [file 44319_2026_759_MOESM4_ESM.zip › Source Data for Figure 2/2G/E14.5-WT.tif]

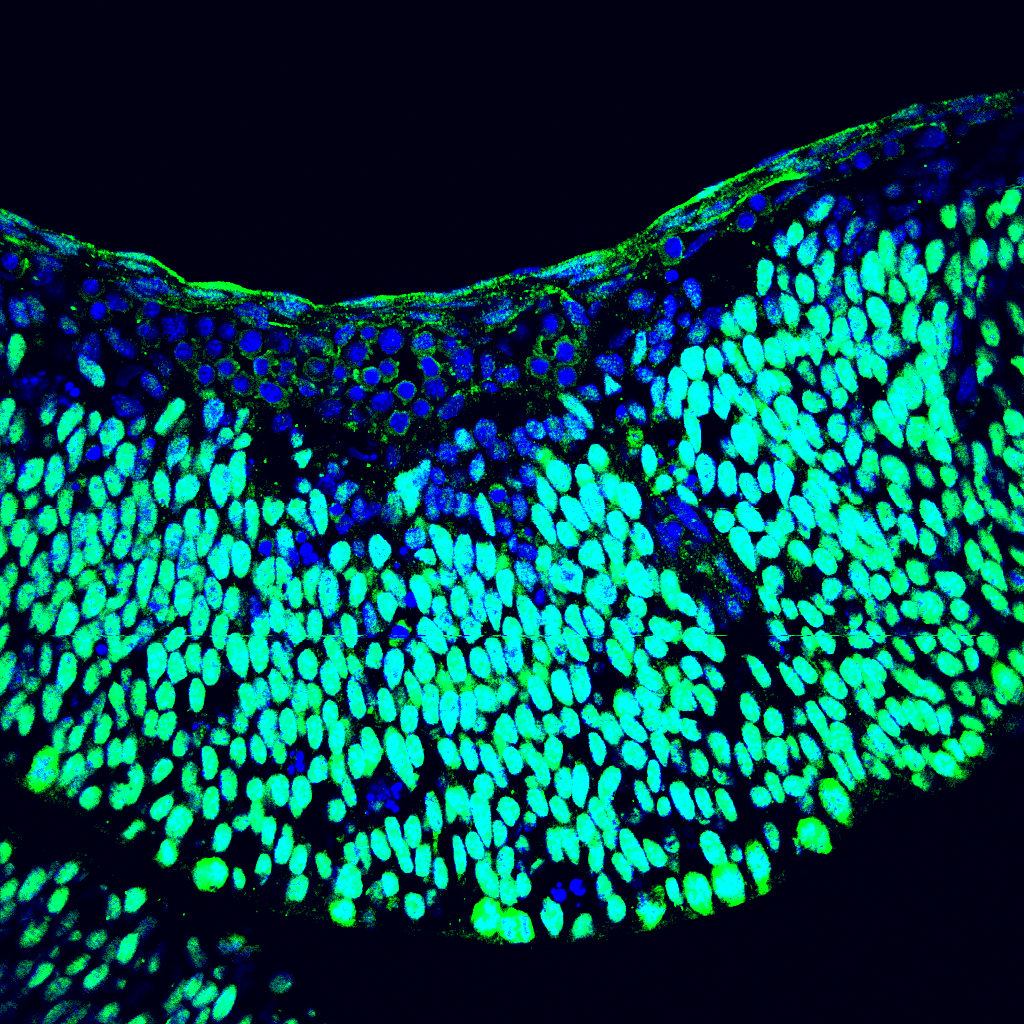

Supplement: Supplementary file 4 — Source data Fig. 2 [file 44319_2026_759_MOESM4_ESM.zip › Source Data for Figure 2/2M/E11.5_tut1_ff__emx1-cre.tif]

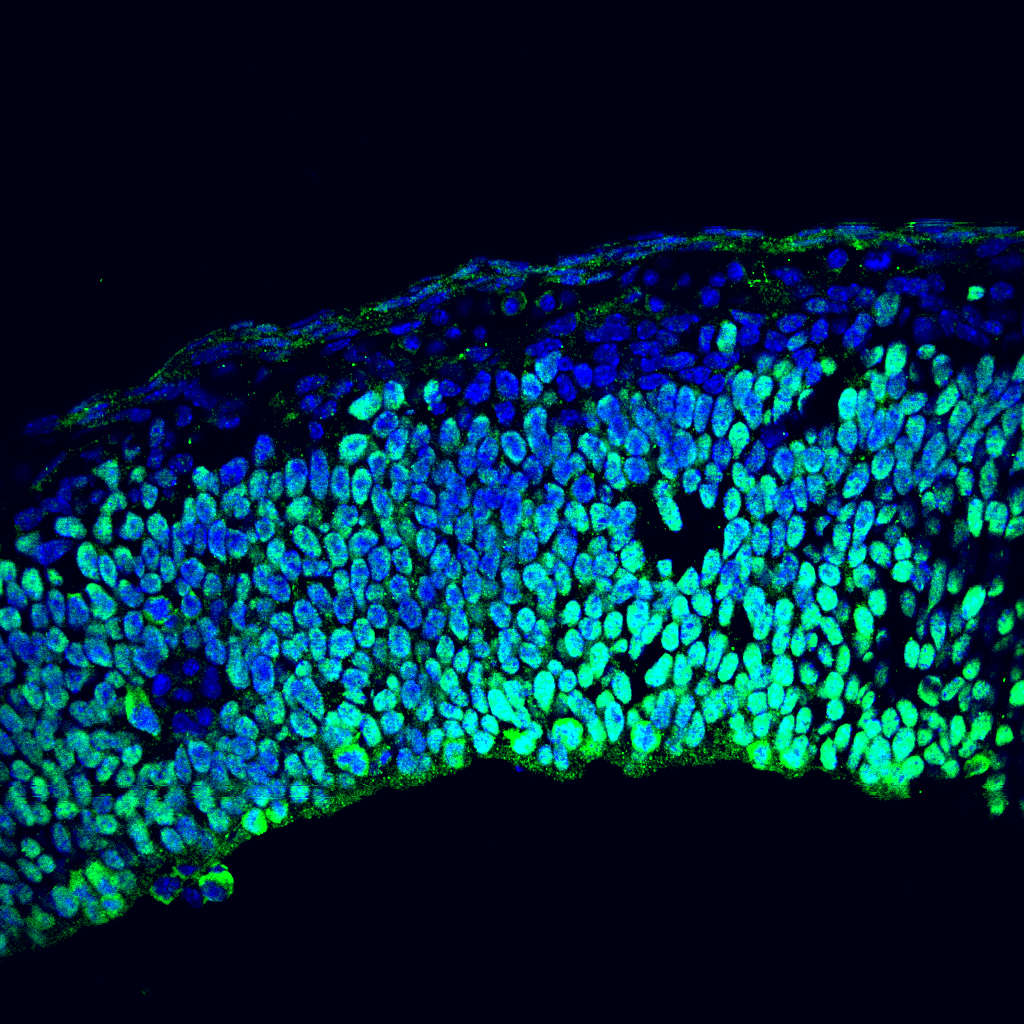

Supplement: Supplementary file 4 — Source data Fig. 2 [file 44319_2026_759_MOESM4_ESM.zip › Source Data for Figure 2/2M/E11.5_WT.tif]

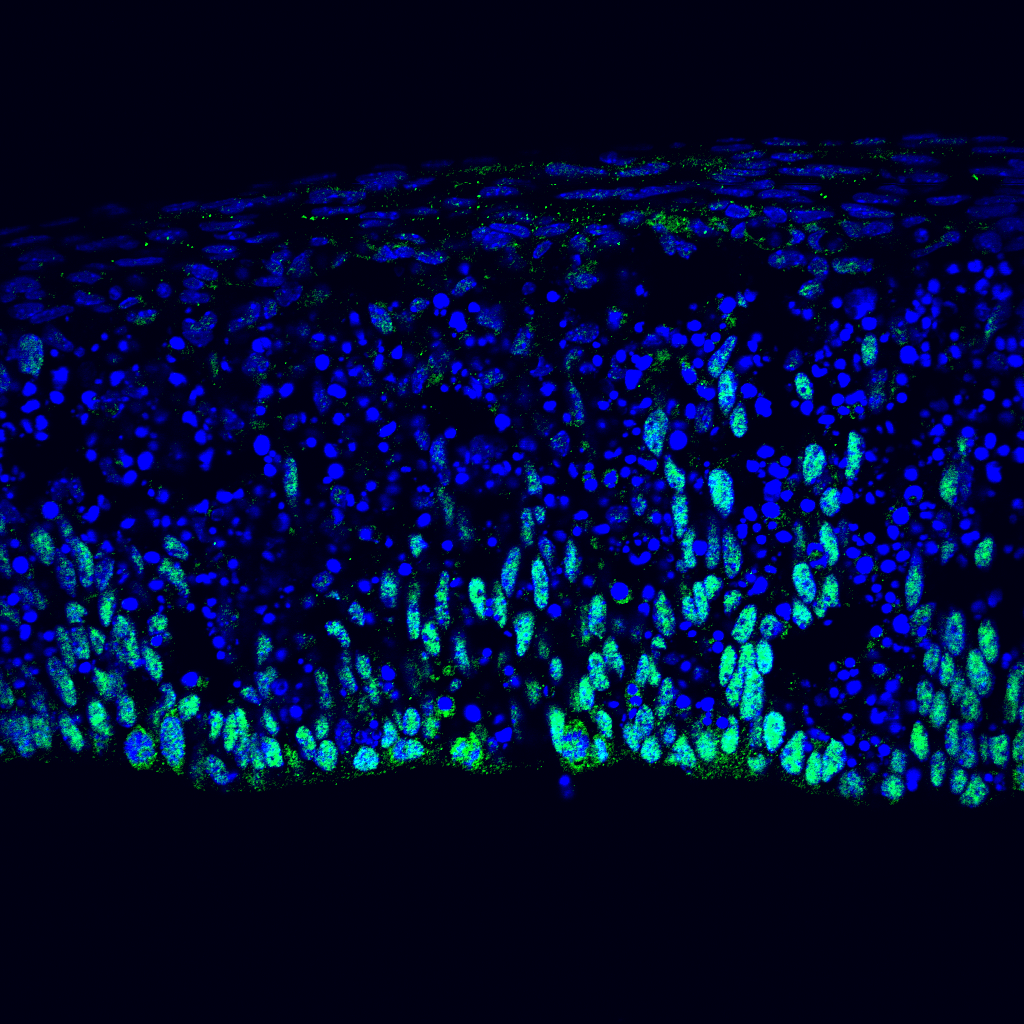

Supplement: Supplementary file 4 — Source data Fig. 2 [file 44319_2026_759_MOESM4_ESM.zip › Source Data for Figure 2/2M/E112.5_tut1_ff__emx1-cre.tif]

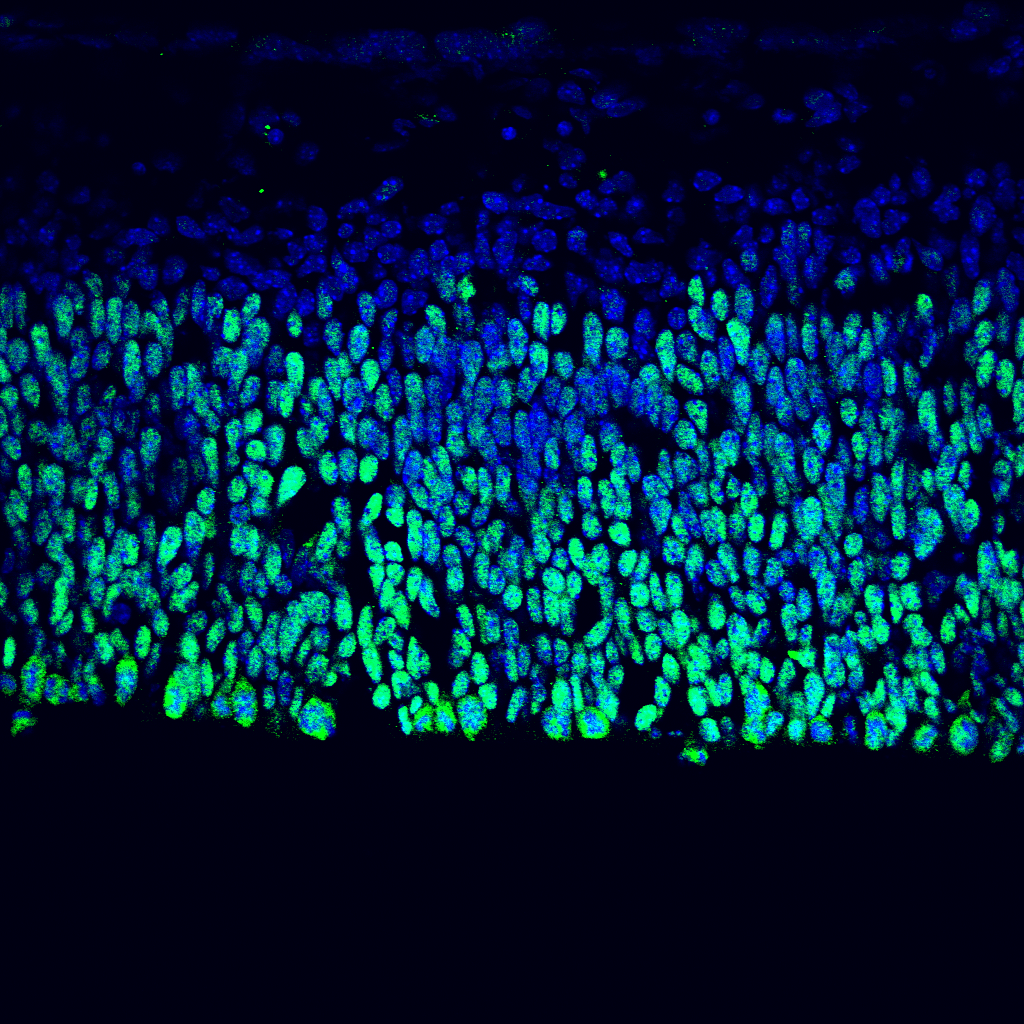

Supplement: Supplementary file 4 — Source data Fig. 2 [file 44319_2026_759_MOESM4_ESM.zip › Source Data for Figure 2/2M/E12.5_WT.tif]

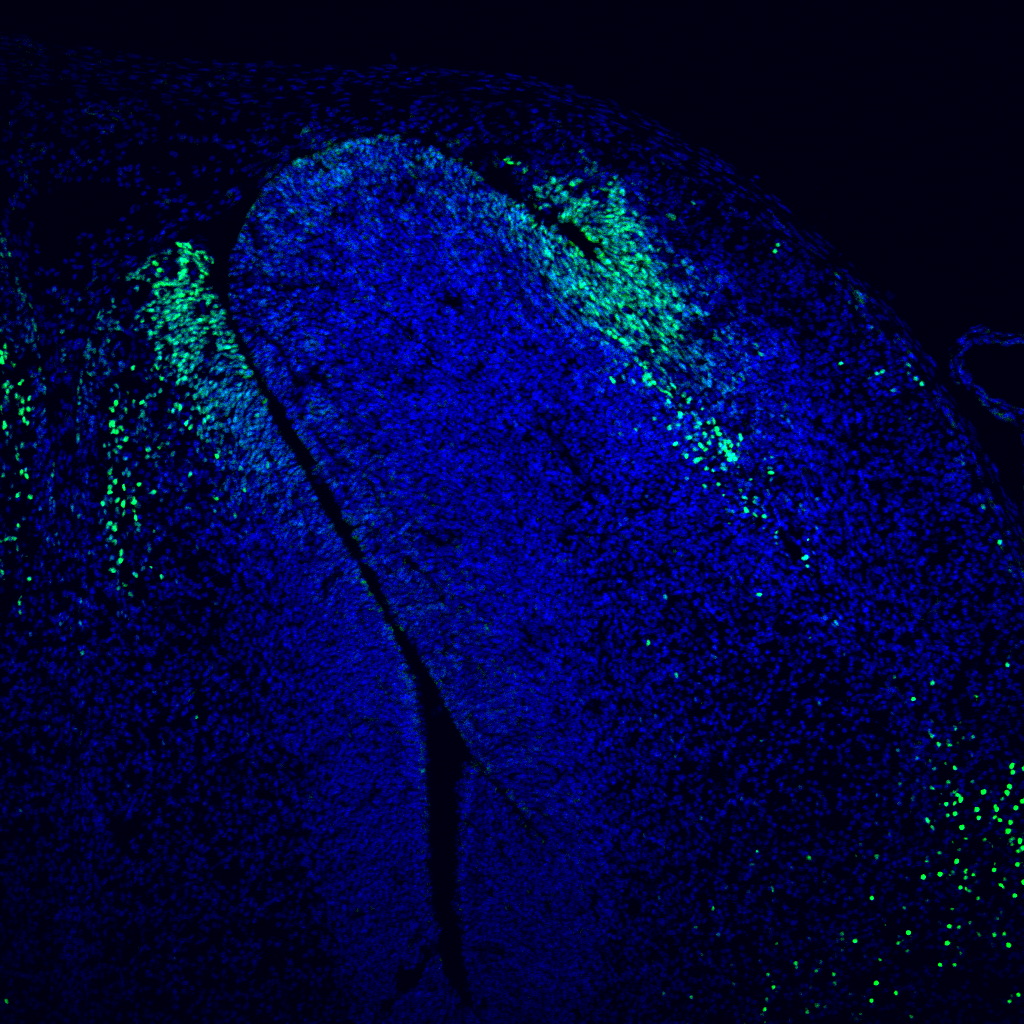

Supplement: Supplementary file 4 — Source data Fig. 2 [file 44319_2026_759_MOESM4_ESM.zip › Source Data for Figure 2/2M/E14.5_tut1_ff__emx1-cre.tif]

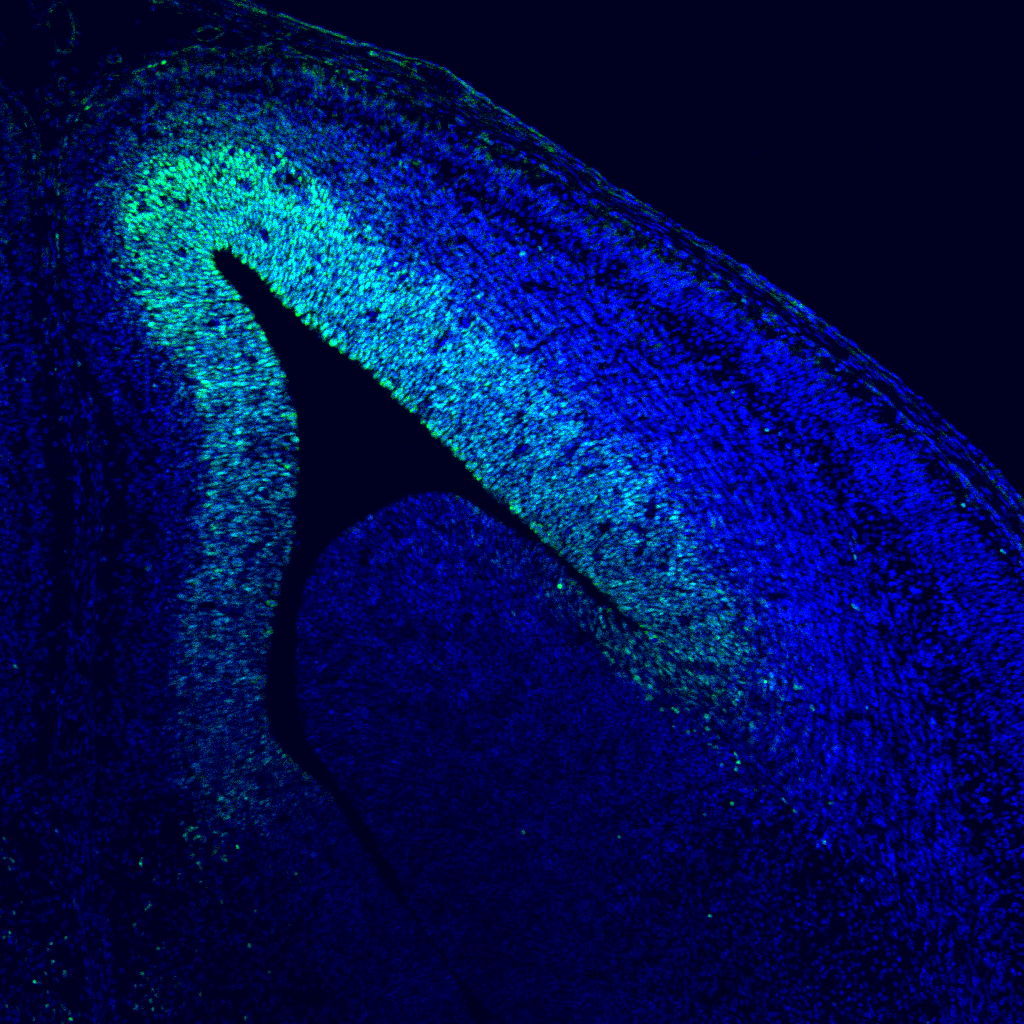

Supplement: Supplementary file 4 — Source data Fig. 2 [file 44319_2026_759_MOESM4_ESM.zip › Source Data for Figure 2/2M/E14.5_WT.tif]

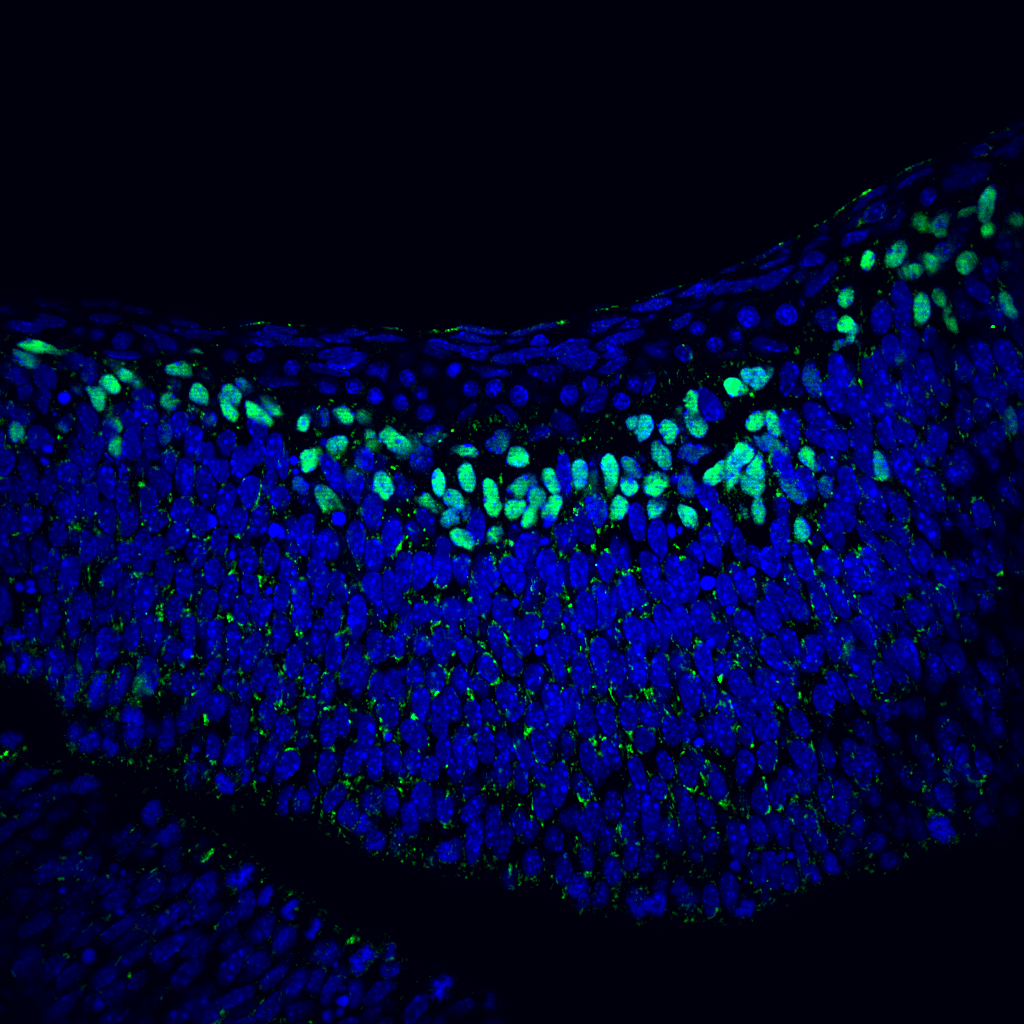

Supplement: Supplementary file 4 — Source data Fig. 2 [file 44319_2026_759_MOESM4_ESM.zip › Source Data for Figure 2/2N/E11.5_tut1_ff__emx1-cre.tif]

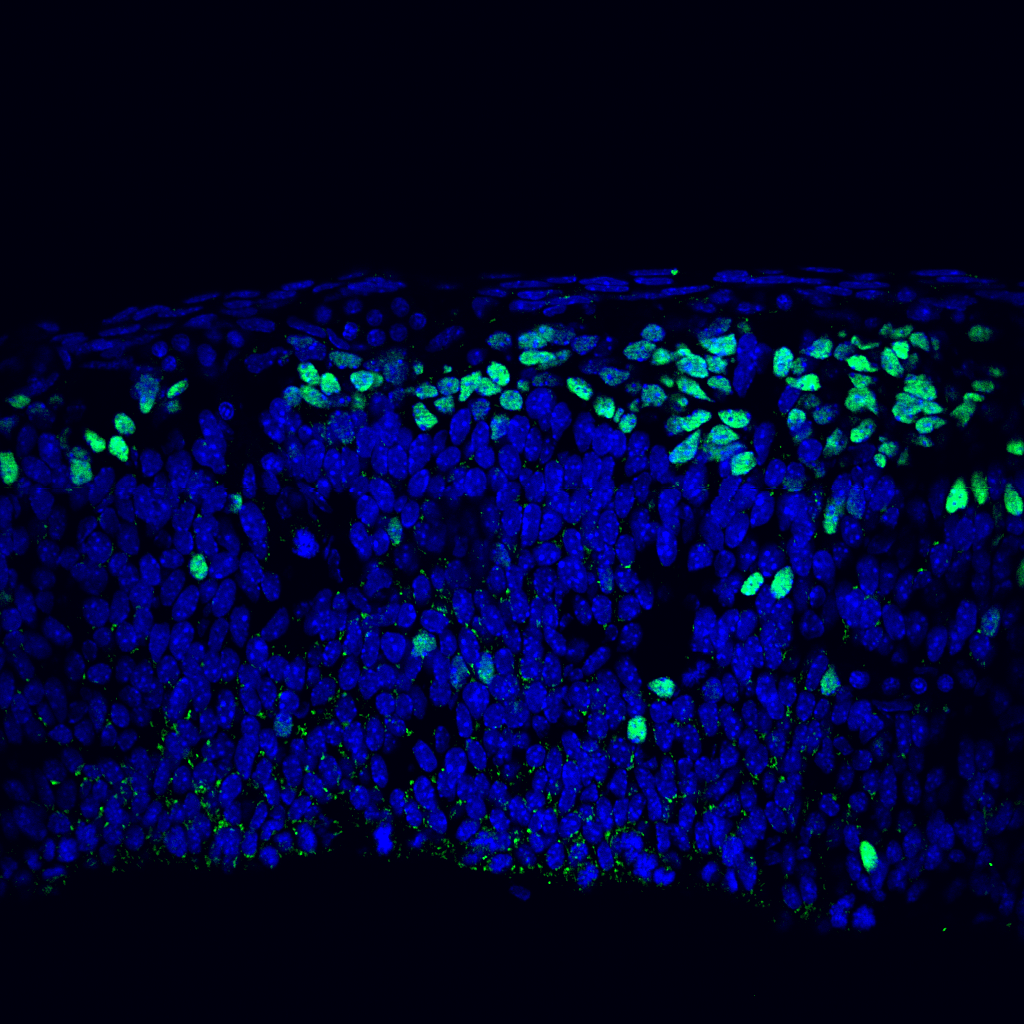

Supplement: Supplementary file 4 — Source data Fig. 2 [file 44319_2026_759_MOESM4_ESM.zip › Source Data for Figure 2/2N/E11.5_WT.tif]

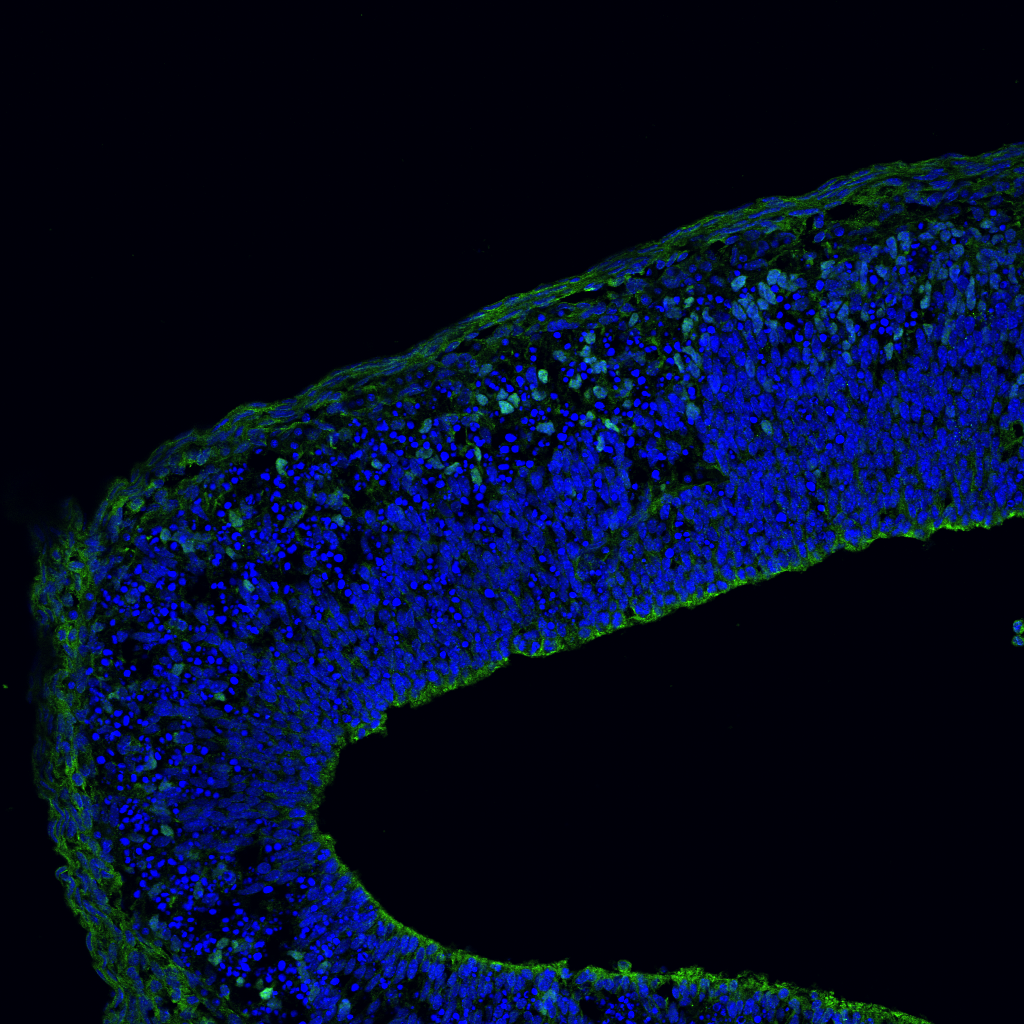

Supplement: Supplementary file 4 — Source data Fig. 2 [file 44319_2026_759_MOESM4_ESM.zip › Source Data for Figure 2/2N/E12.5_tut1_ff__emx1-cre.tif]

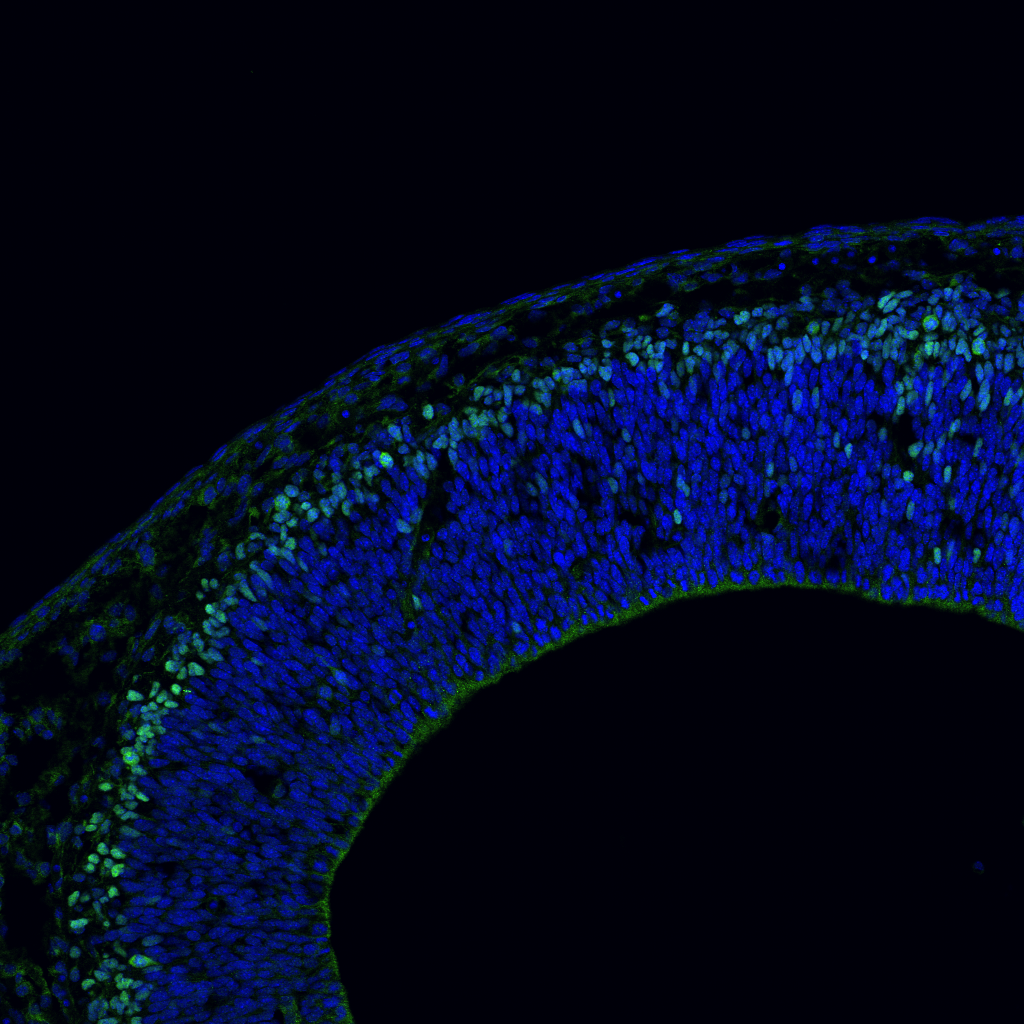

Supplement: Supplementary file 4 — Source data Fig. 2 [file 44319_2026_759_MOESM4_ESM.zip › Source Data for Figure 2/2N/E12.5_WT.tif]

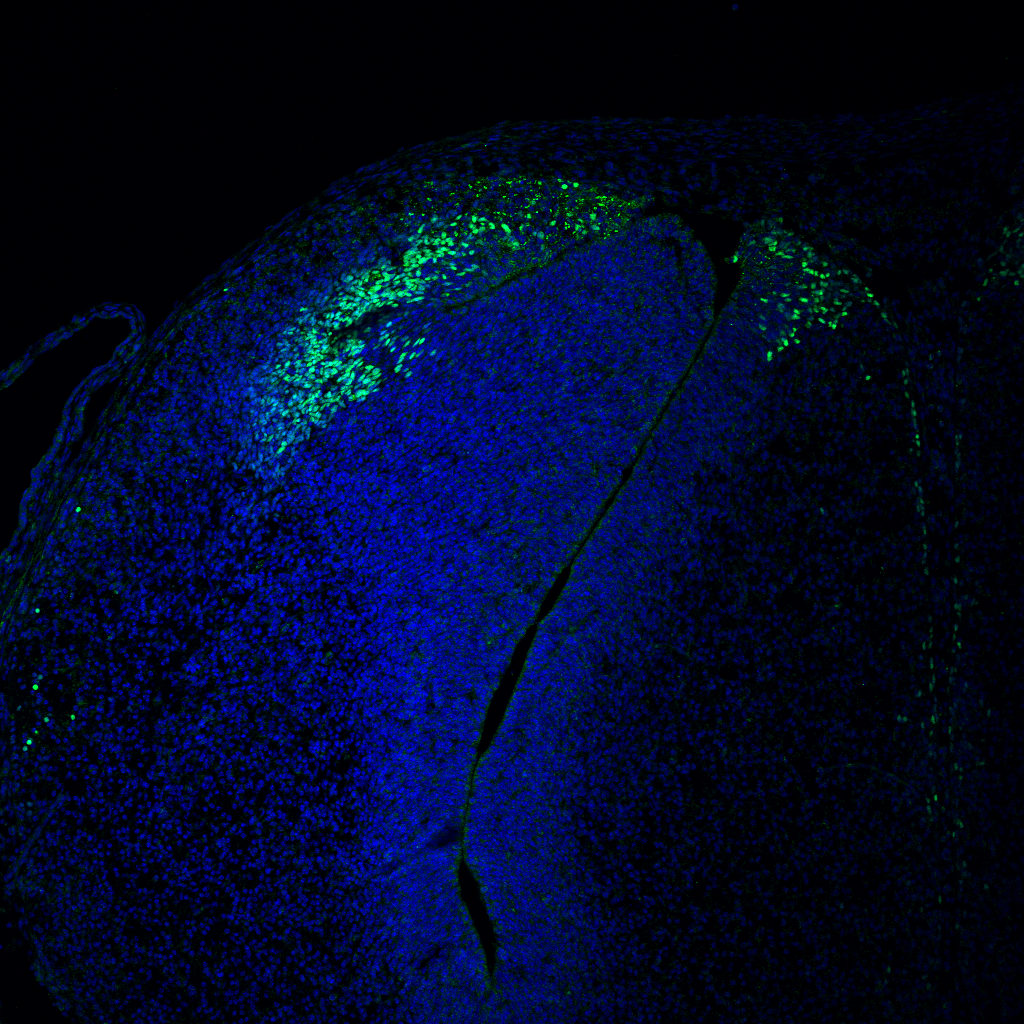

Supplement: Supplementary file 4 — Source data Fig. 2 [file 44319_2026_759_MOESM4_ESM.zip › Source Data for Figure 2/2N/E14.5_tut1_ff__emx1-cre.tif]

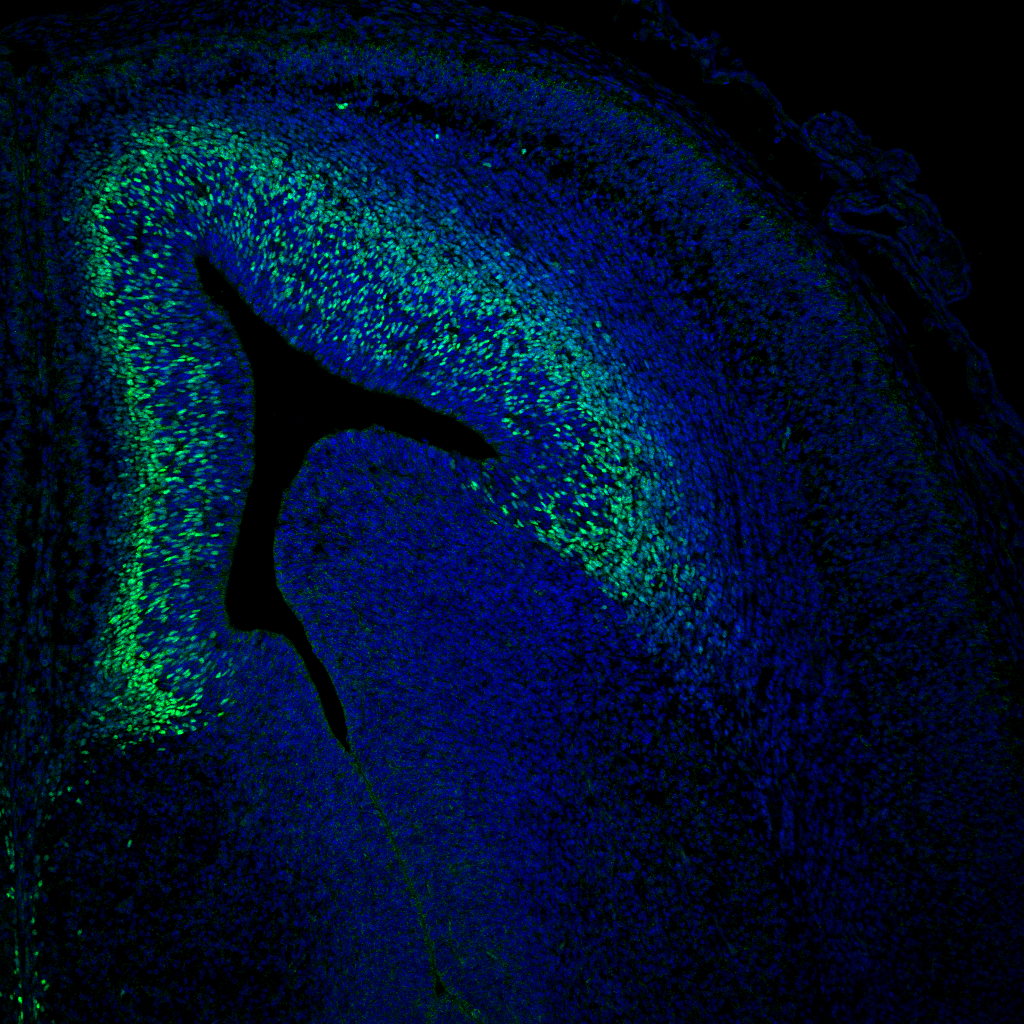

Supplement: Supplementary file 4 — Source data Fig. 2 [file 44319_2026_759_MOESM4_ESM.zip › Source Data for Figure 2/2N/E14.5_WT.tif]

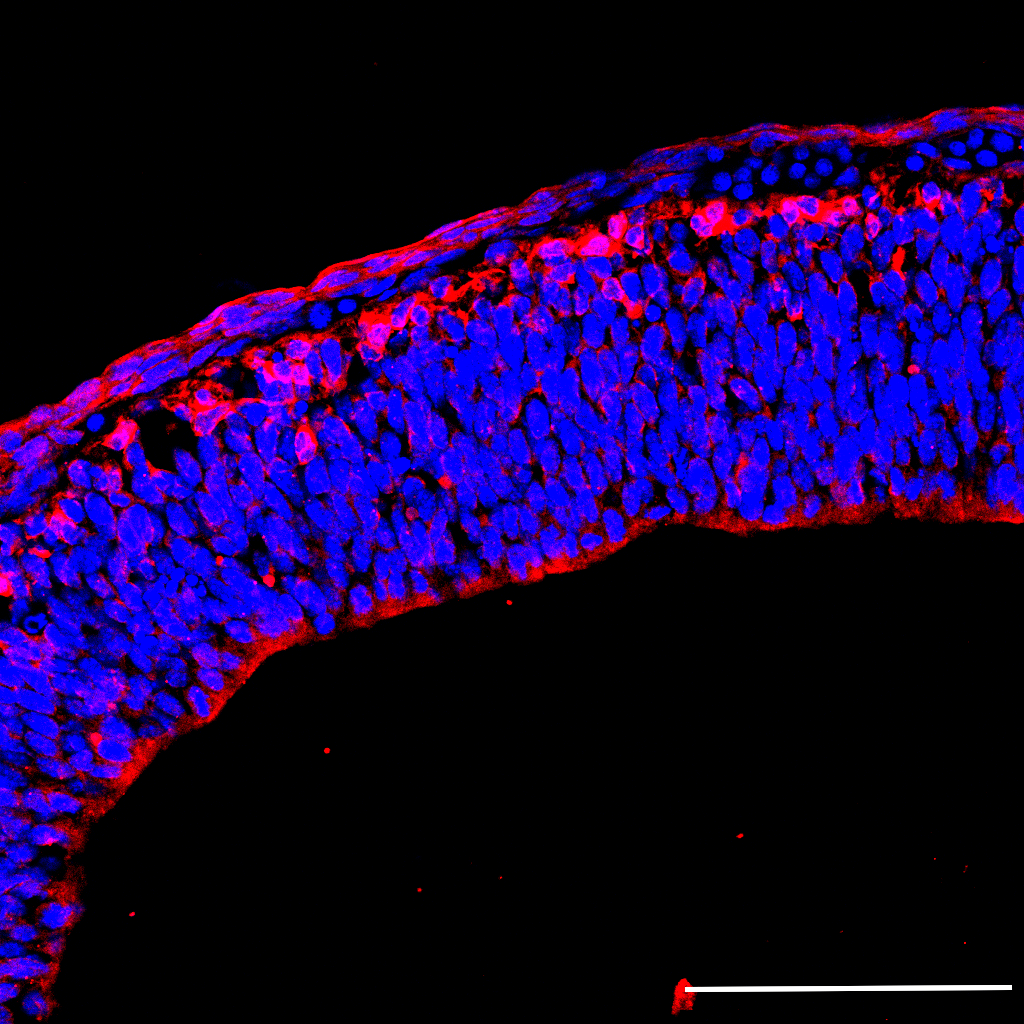

Supplement: Supplementary file 4 — Source data Fig. 2 [file 44319_2026_759_MOESM4_ESM.zip › Source Data for Figure 2/2O/E11.5_tut1_ff__emx1-cre.tif]

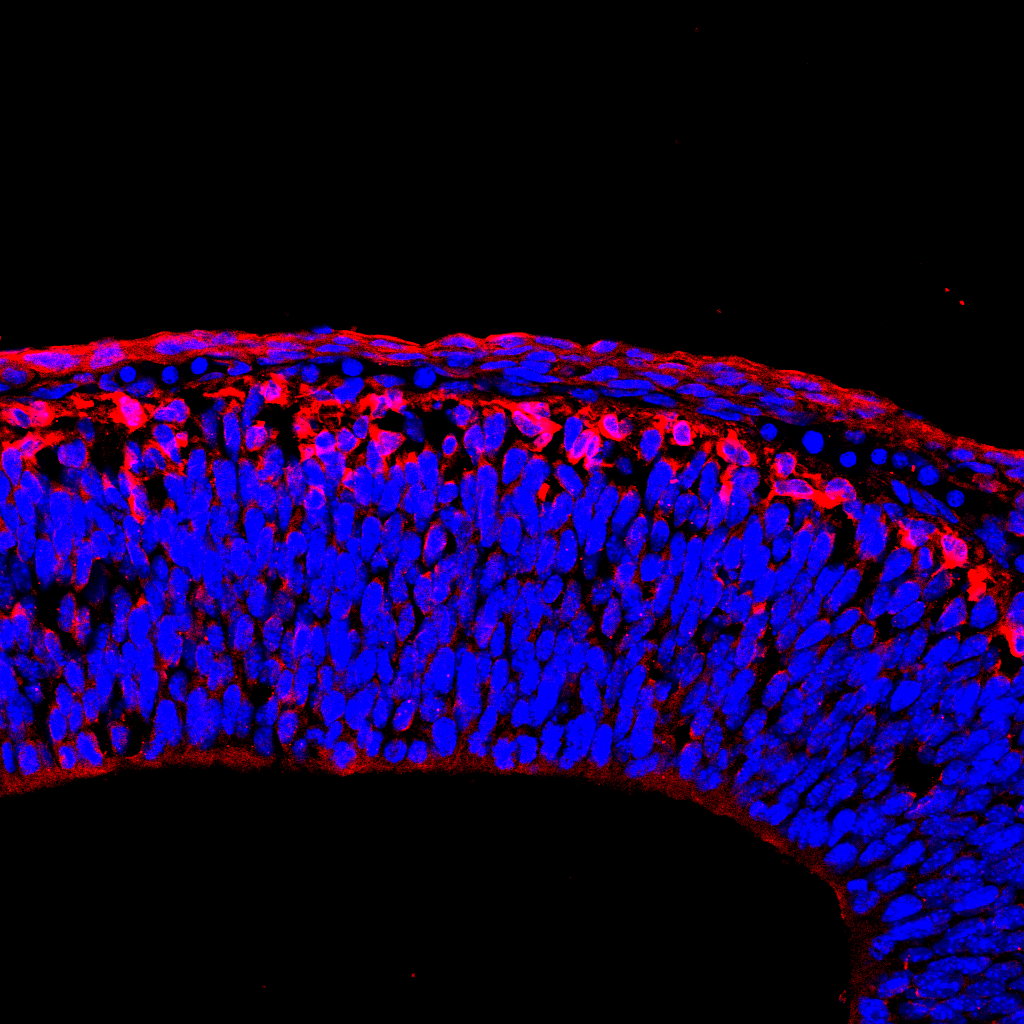

Supplement: Supplementary file 4 — Source data Fig. 2 [file 44319_2026_759_MOESM4_ESM.zip › Source Data for Figure 2/2O/E11.5_WT.tif]

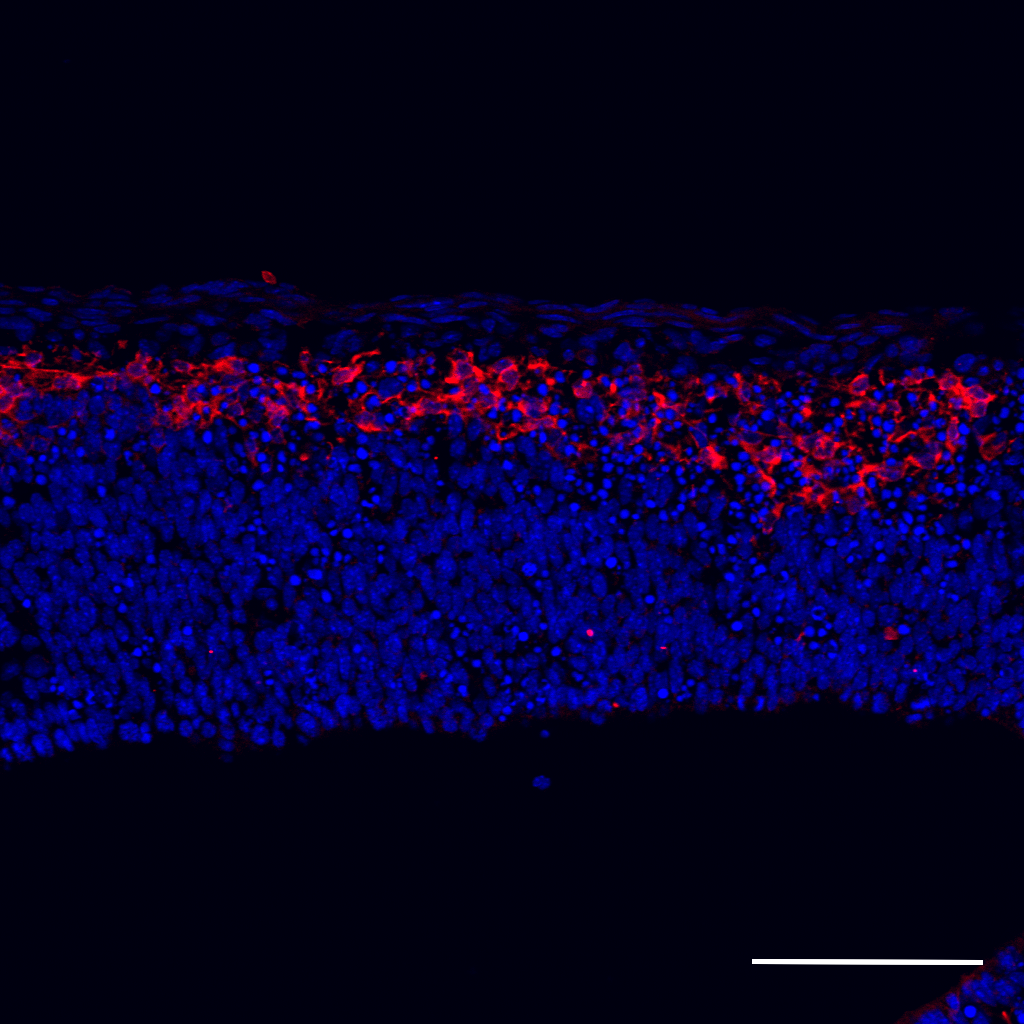

Supplement: Supplementary file 4 — Source data Fig. 2 [file 44319_2026_759_MOESM4_ESM.zip › Source Data for Figure 2/2O/E12.5_tut1_ff__emx1-cre.tif]

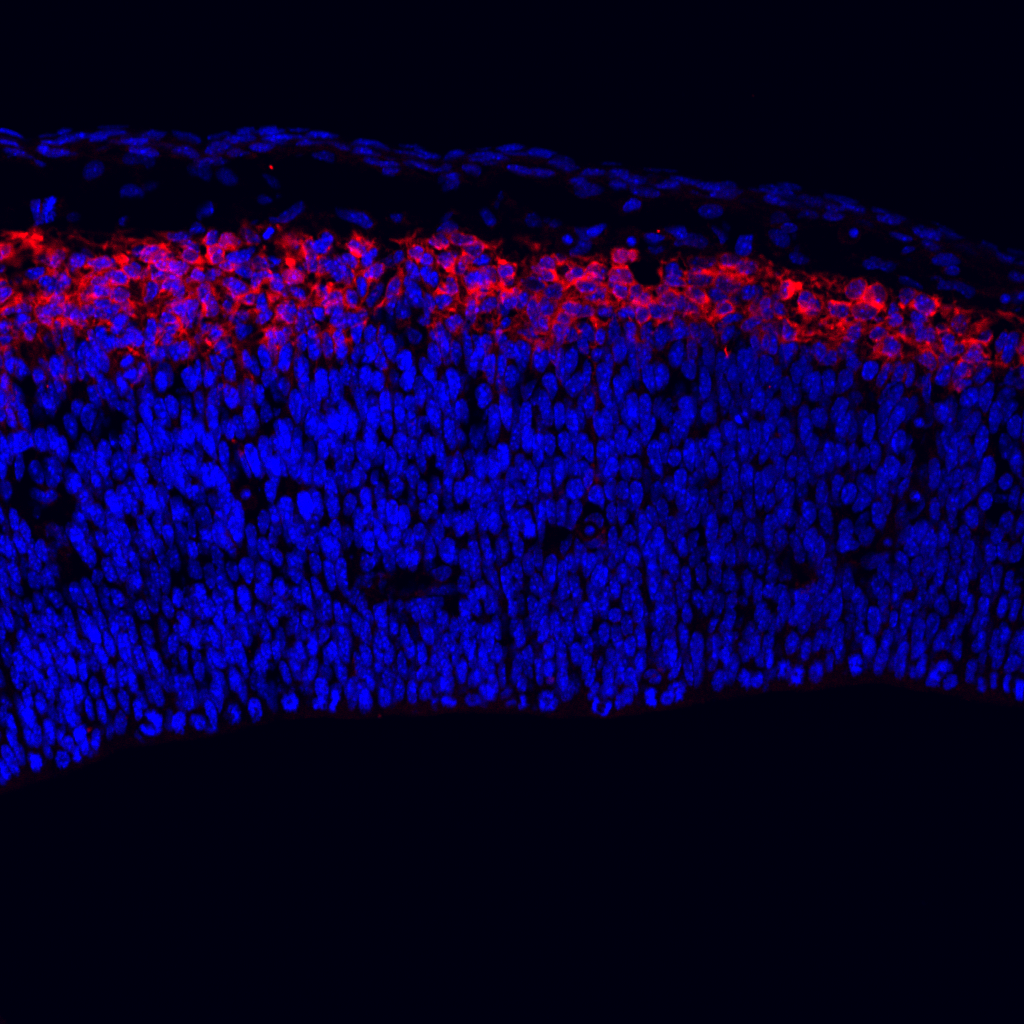

Supplement: Supplementary file 4 — Source data Fig. 2 [file 44319_2026_759_MOESM4_ESM.zip › Source Data for Figure 2/2O/e12.5_WT.tif]

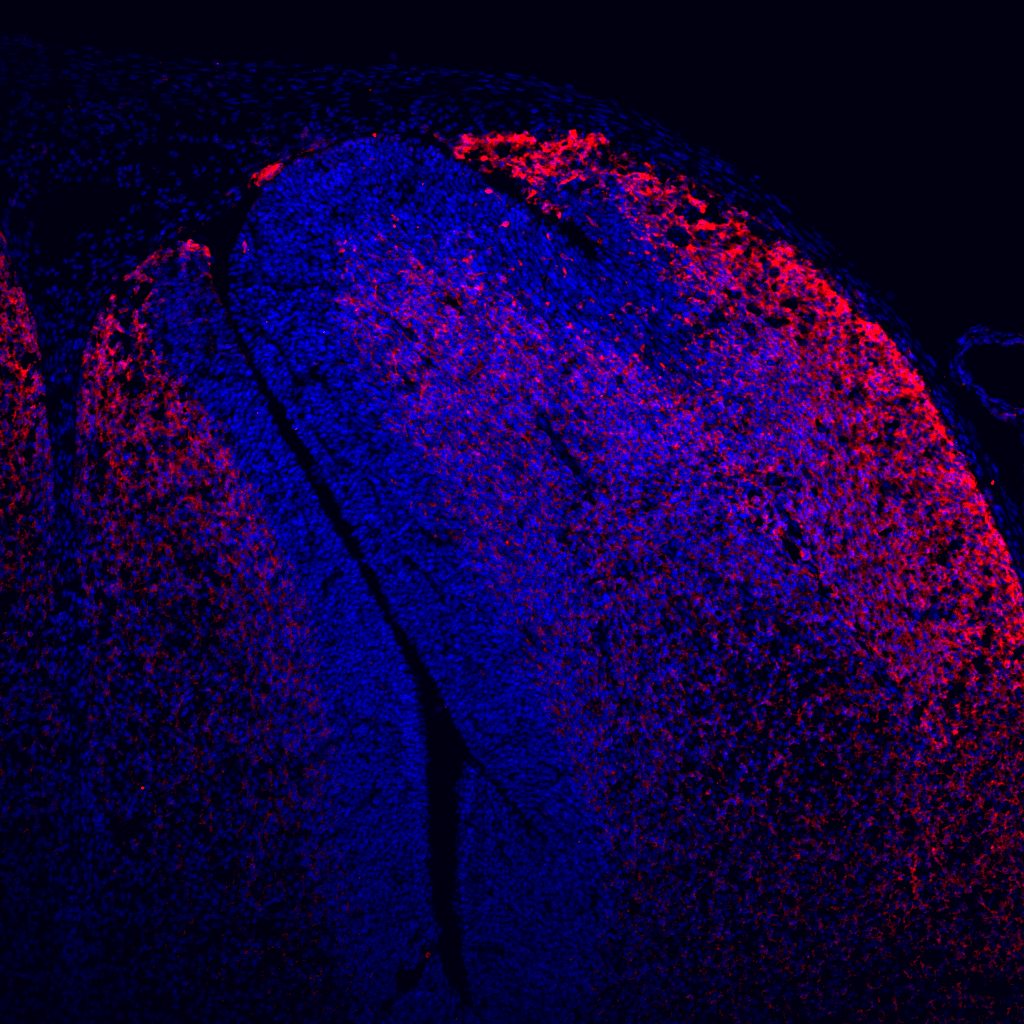

Supplement: Supplementary file 4 — Source data Fig. 2 [file 44319_2026_759_MOESM4_ESM.zip › Source Data for Figure 2/2O/E14.5_tut1_ff__emx1-cre.tif]

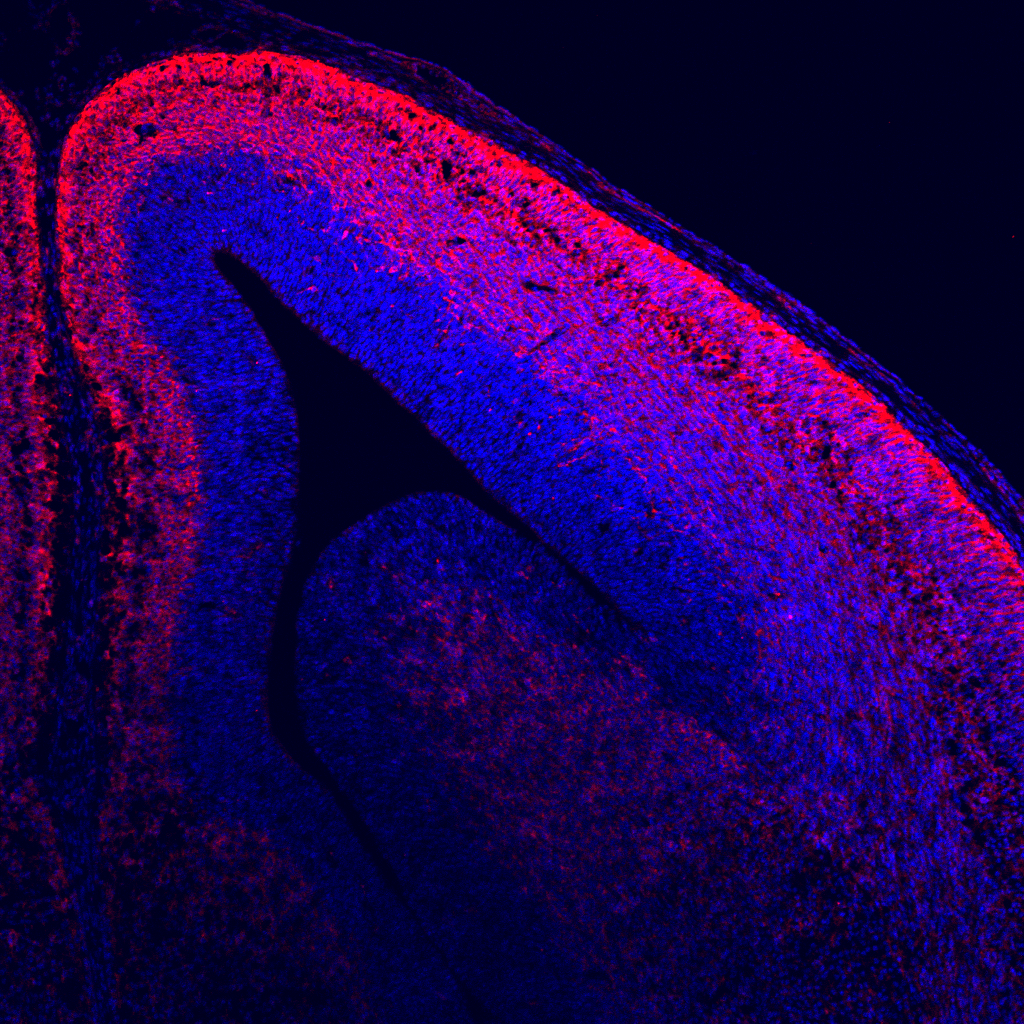

Supplement: Supplementary file 4 — Source data Fig. 2 [file 44319_2026_759_MOESM4_ESM.zip › Source Data for Figure 2/2O/e14.5_WT.tif]

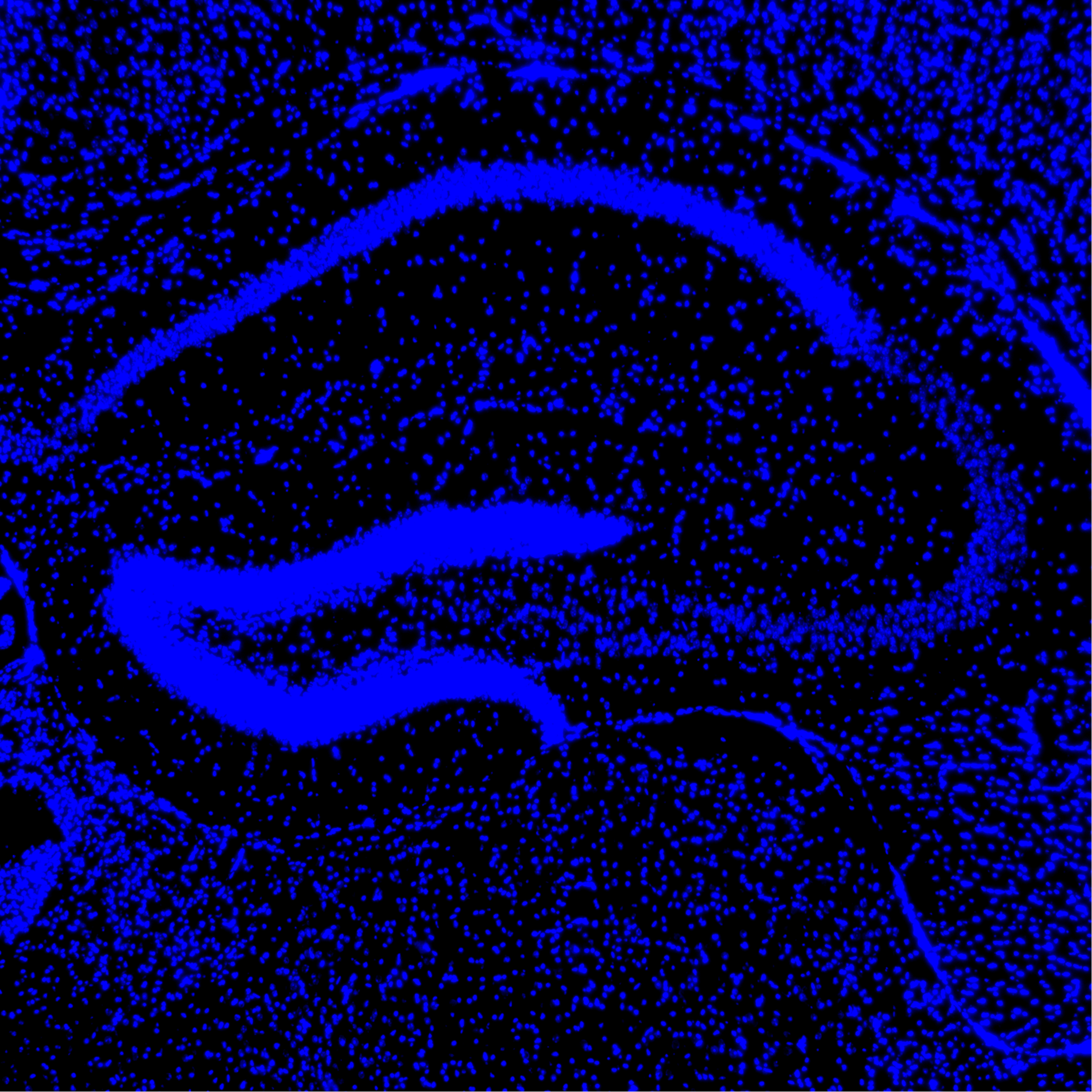

Supplement: Supplementary file 4 — Source data Fig. 2 [file 44319_2026_759_MOESM4_ESM.zip › Source Data for Figure 2/2S/Tut1ff;camk2a-cre.tif]

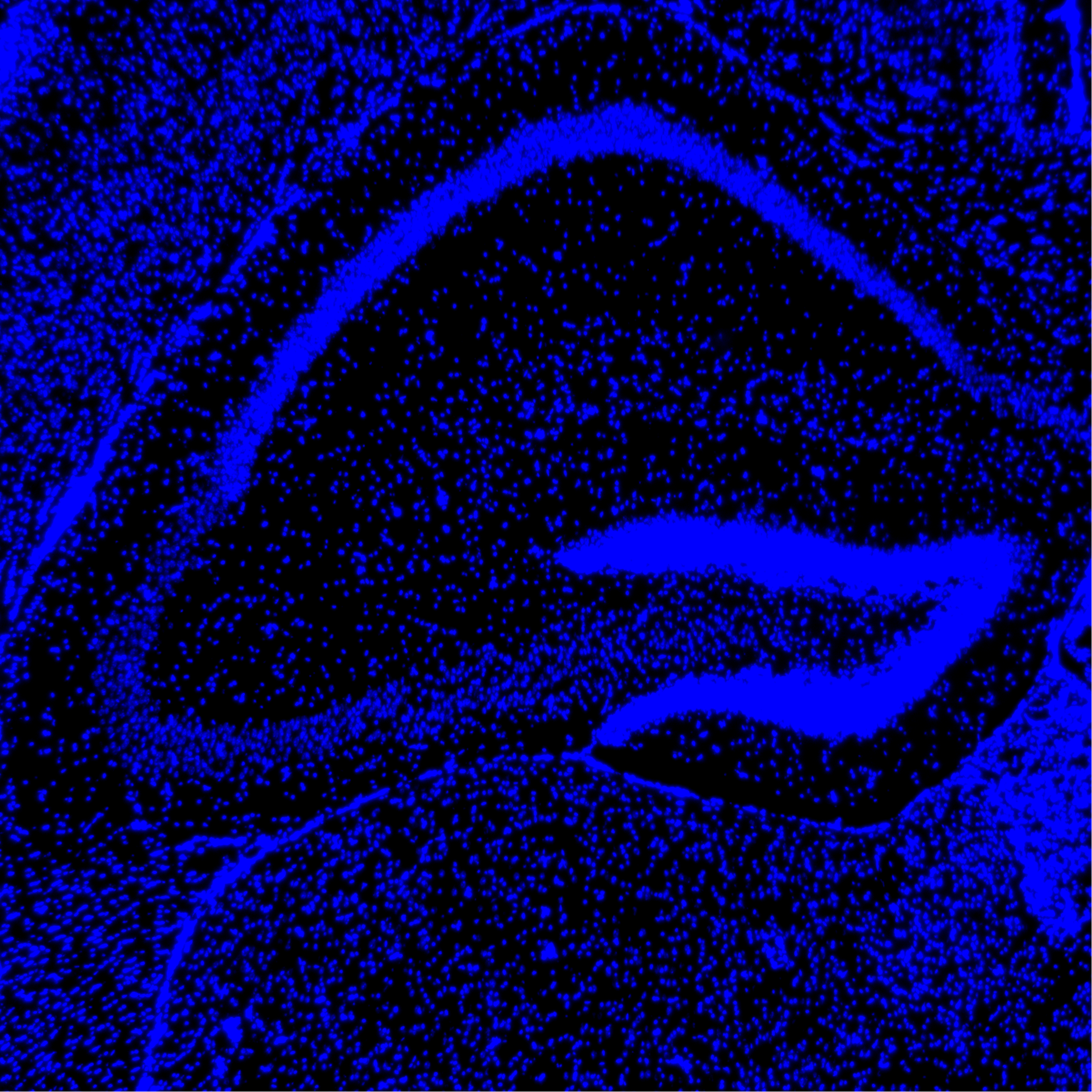

Supplement: Supplementary file 4 — Source data Fig. 2 [file 44319_2026_759_MOESM4_ESM.zip › Source Data for Figure 2/2S/WT.tif]

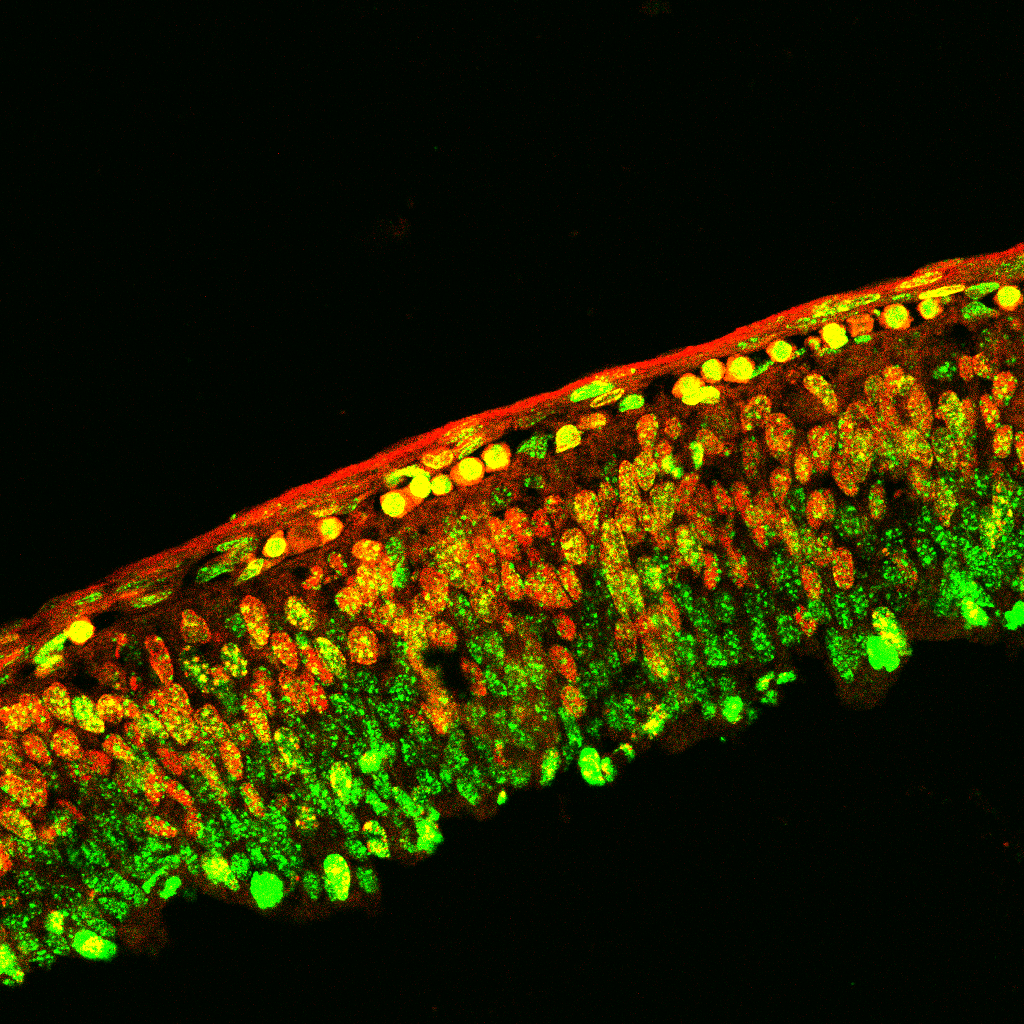

Supplement: Supplementary file 5 — Source data Fig. 3 [file 44319_2026_759_MOESM5_ESM.zip › Source Data for Figure 3/Fig. 3A/e11.5_tut1_ff_emx1-Cre.tif]

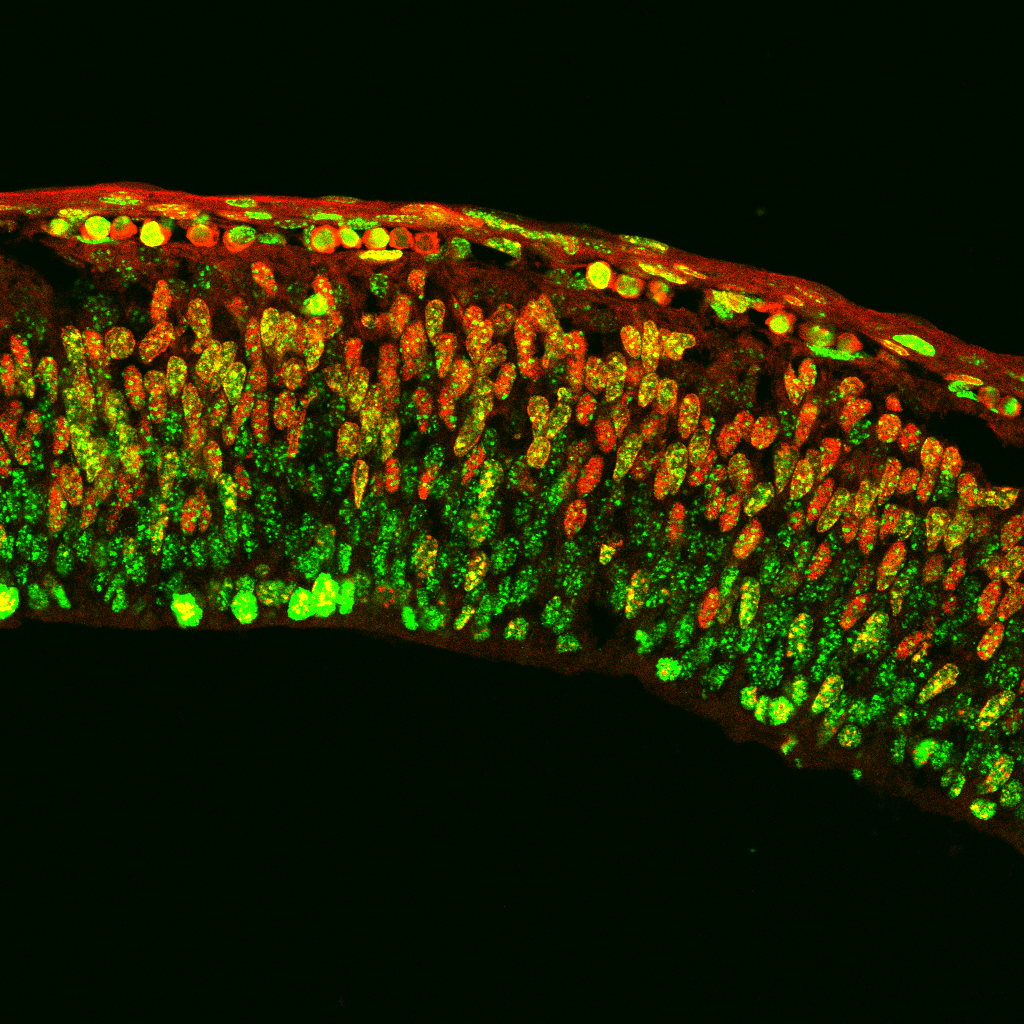

Supplement: Supplementary file 5 — Source data Fig. 3 [file 44319_2026_759_MOESM5_ESM.zip › Source Data for Figure 3/Fig. 3A/e11.5_WT.tif]

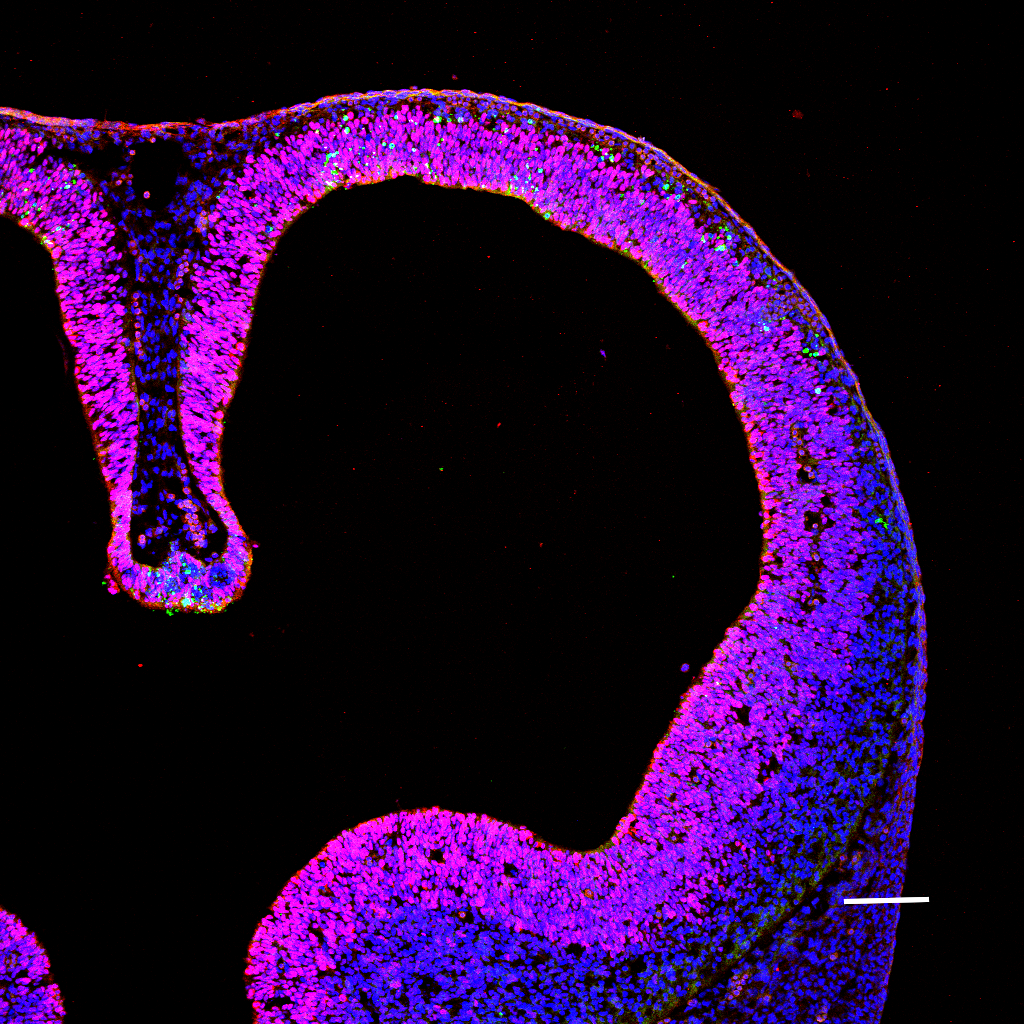

Supplement: Supplementary file 5 — Source data Fig. 3 [file 44319_2026_759_MOESM5_ESM.zip › Source Data for Figure 3/Fig. 3B/e11.5_tut1_ff_emx1-cre.tif]

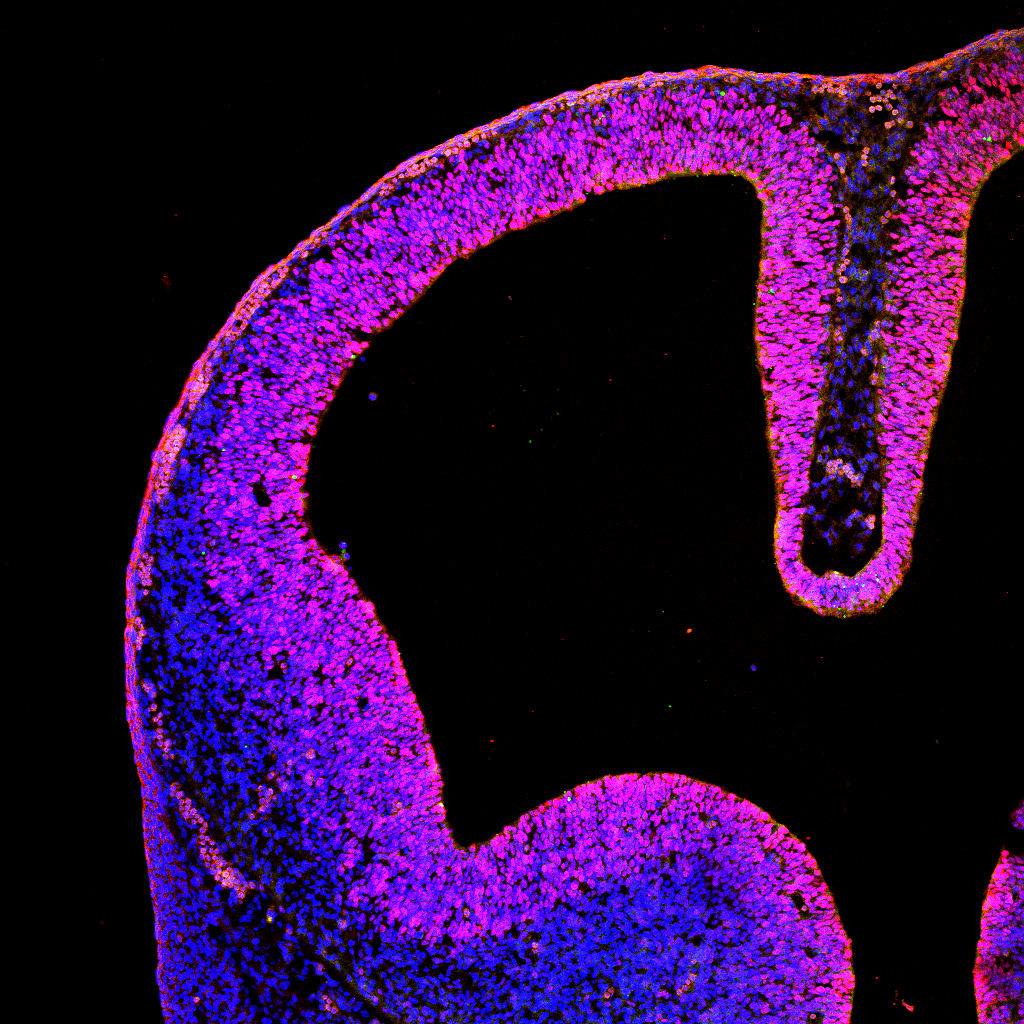

Supplement: Supplementary file 5 — Source data Fig. 3 [file 44319_2026_759_MOESM5_ESM.zip › Source Data for Figure 3/Fig. 3B/e11.5_WT.tif]

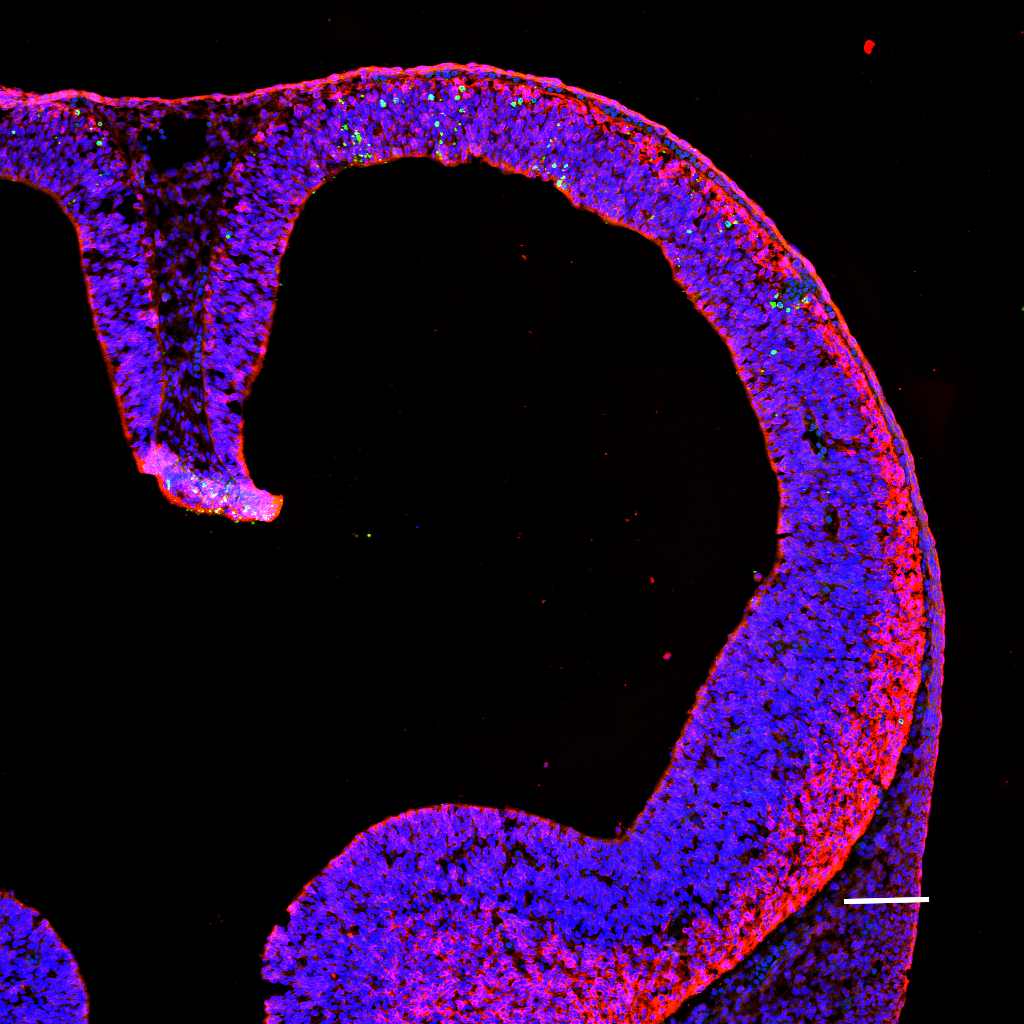

Supplement: Supplementary file 5 — Source data Fig. 3 [file 44319_2026_759_MOESM5_ESM.zip › Source Data for Figure 3/Fig. 3C/e11.5_tut1 ff_emx1_Cre.tif]

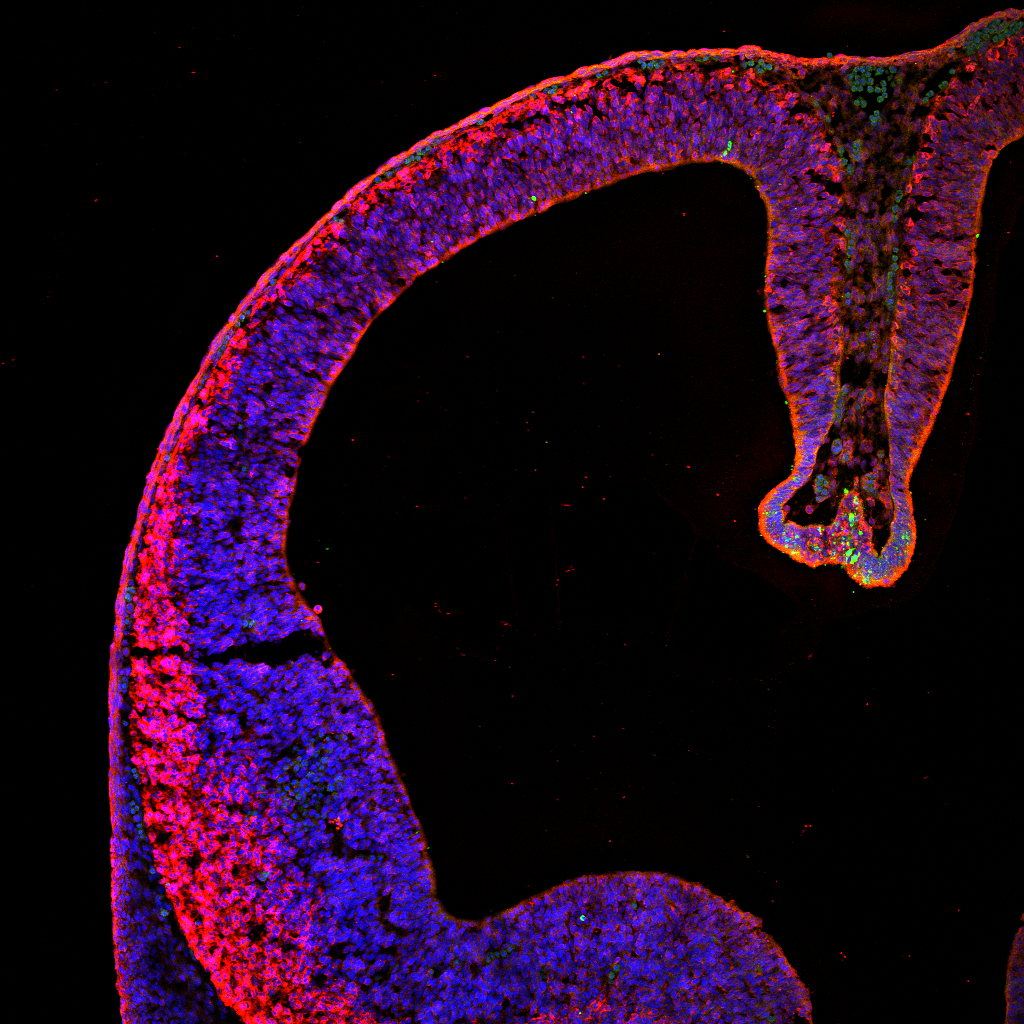

Supplement: Supplementary file 5 — Source data Fig. 3 [file 44319_2026_759_MOESM5_ESM.zip › Source Data for Figure 3/Fig. 3C/e11.5_WT.tif]

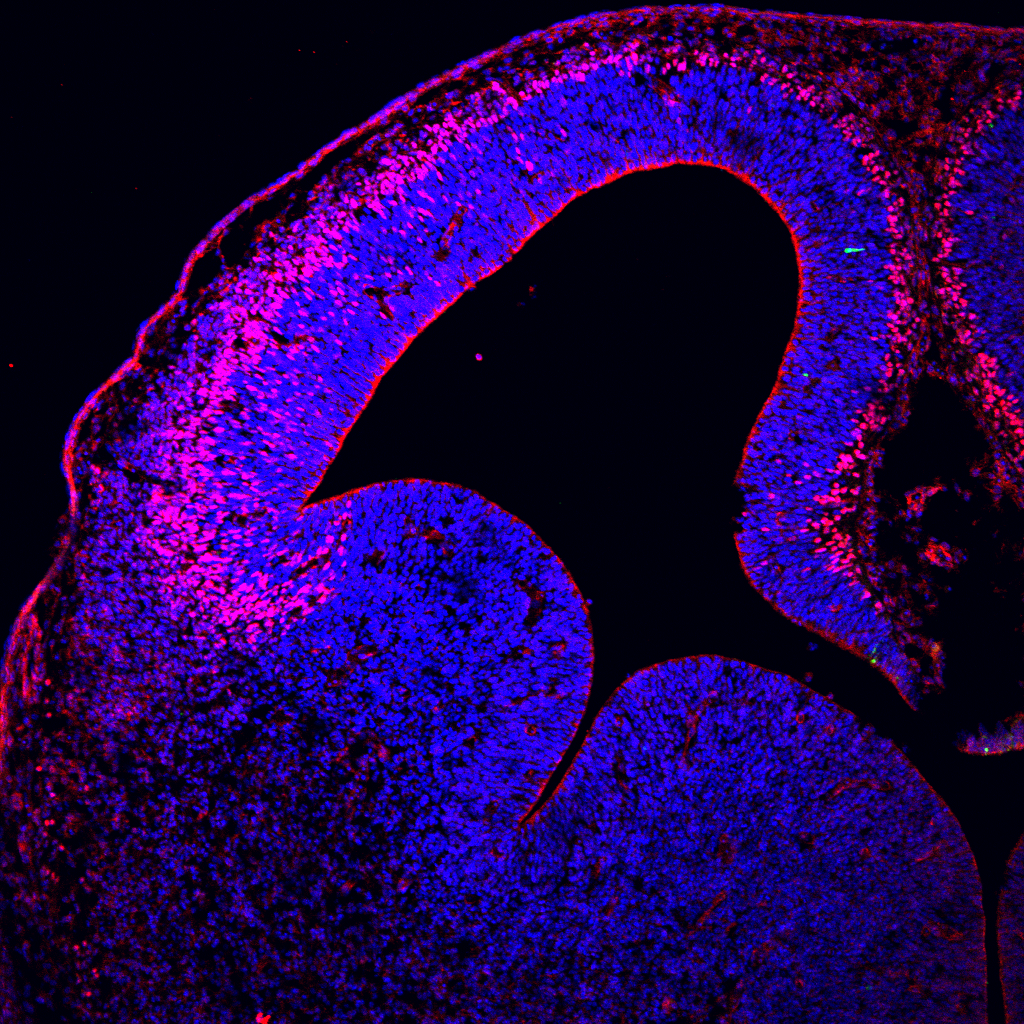

Supplement: Supplementary file 5 — Source data Fig. 3 [file 44319_2026_759_MOESM5_ESM.zip › Source Data for Figure 3/Fig. 3D/e12.5_tut1_cc_cy3_tbr2_488_cleaved casp3_10x_1_T001.tif]

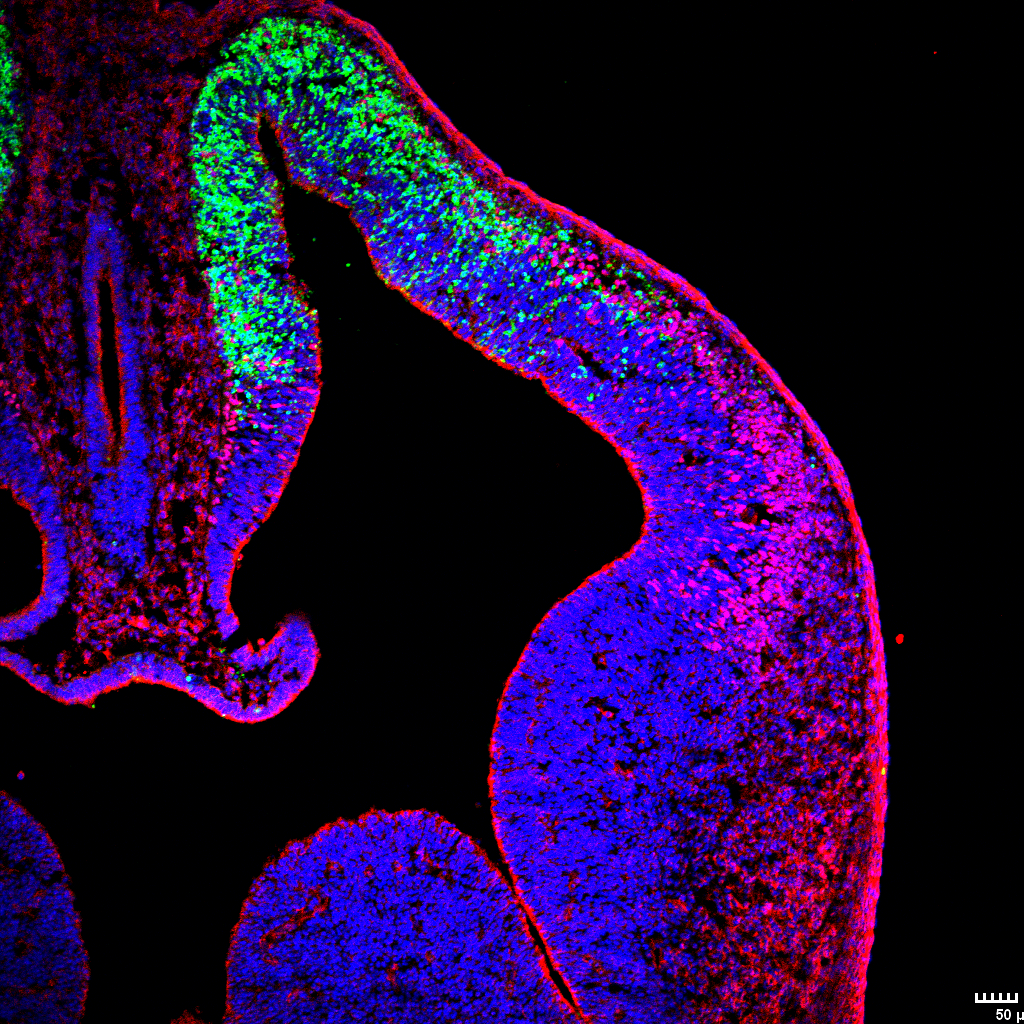

Supplement: Supplementary file 5 — Source data Fig. 3 [file 44319_2026_759_MOESM5_ESM.zip › Source Data for Figure 3/Fig. 3D/e12.5_tut1_cc_emx1 cre_cy3_tbr2_488_cleaved casp3_10x_1_T001.tif]

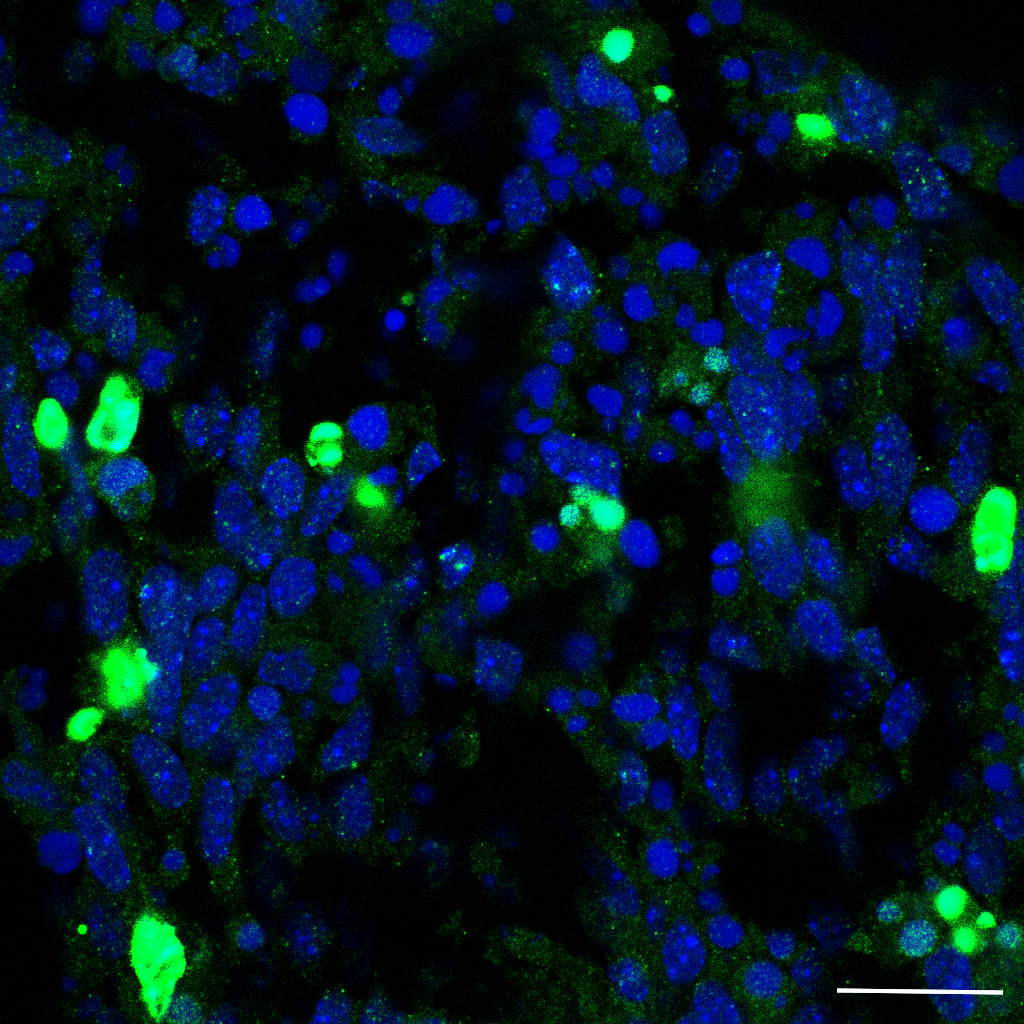

Supplement: Supplementary file 5 — Source data Fig. 3 [file 44319_2026_759_MOESM5_ESM.zip › Source Data for Figure 3/Fig. 3E/e12.5_tut1_ff_emx1-CRE.tif]

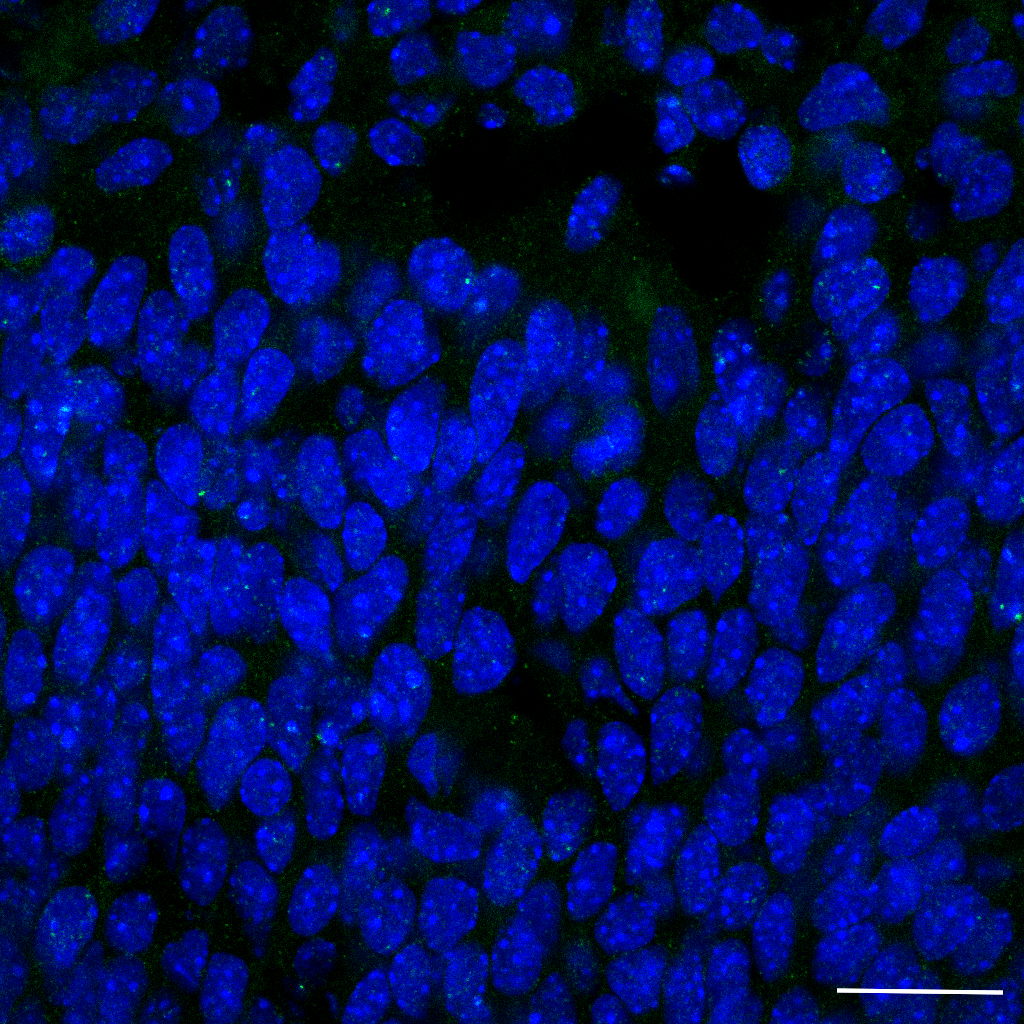

Supplement: Supplementary file 5 — Source data Fig. 3 [file 44319_2026_759_MOESM5_ESM.zip › Source Data for Figure 3/Fig. 3E/e12.5_WT.tif]

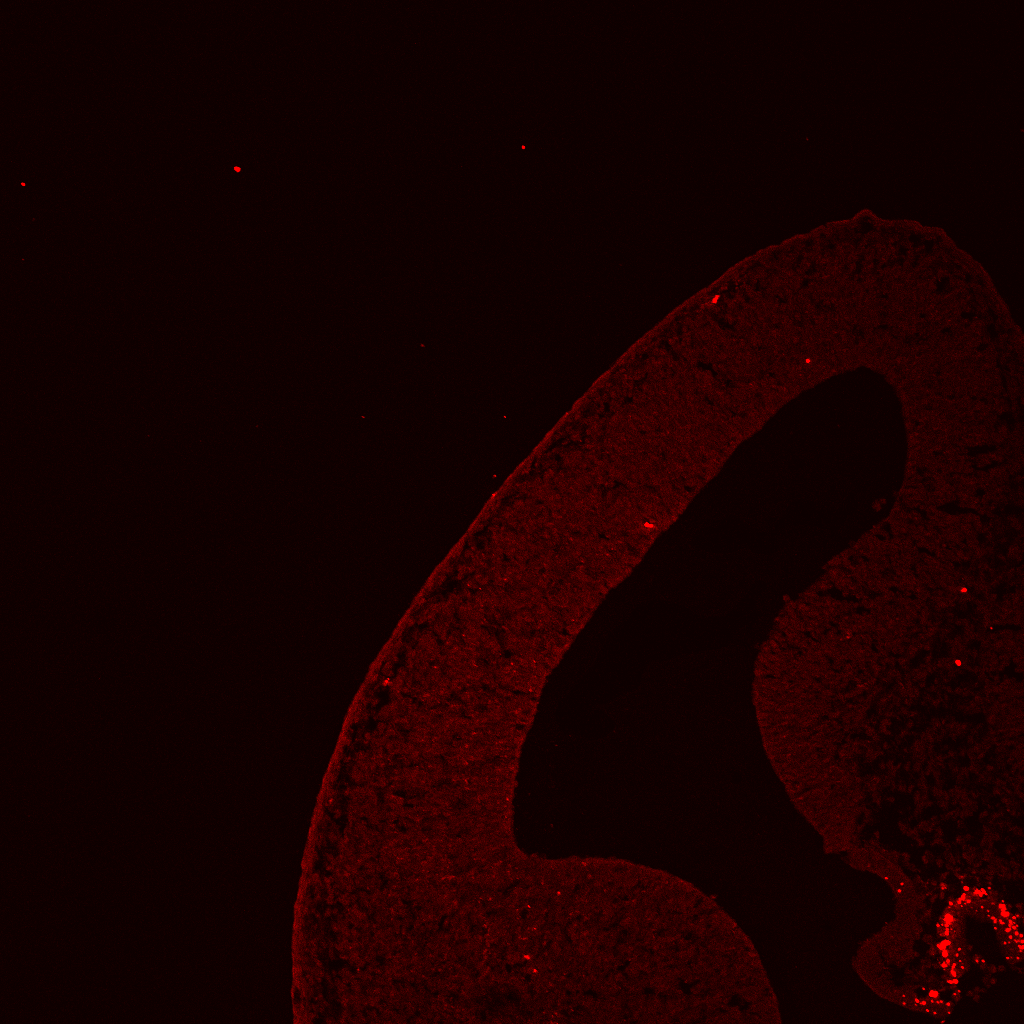

Supplement: Supplementary file 5 — Source data Fig. 3 [file 44319_2026_759_MOESM5_ESM.zip › Source Data for Figure 3/Fig. 3F/e12.5_tunel_wt.tif]

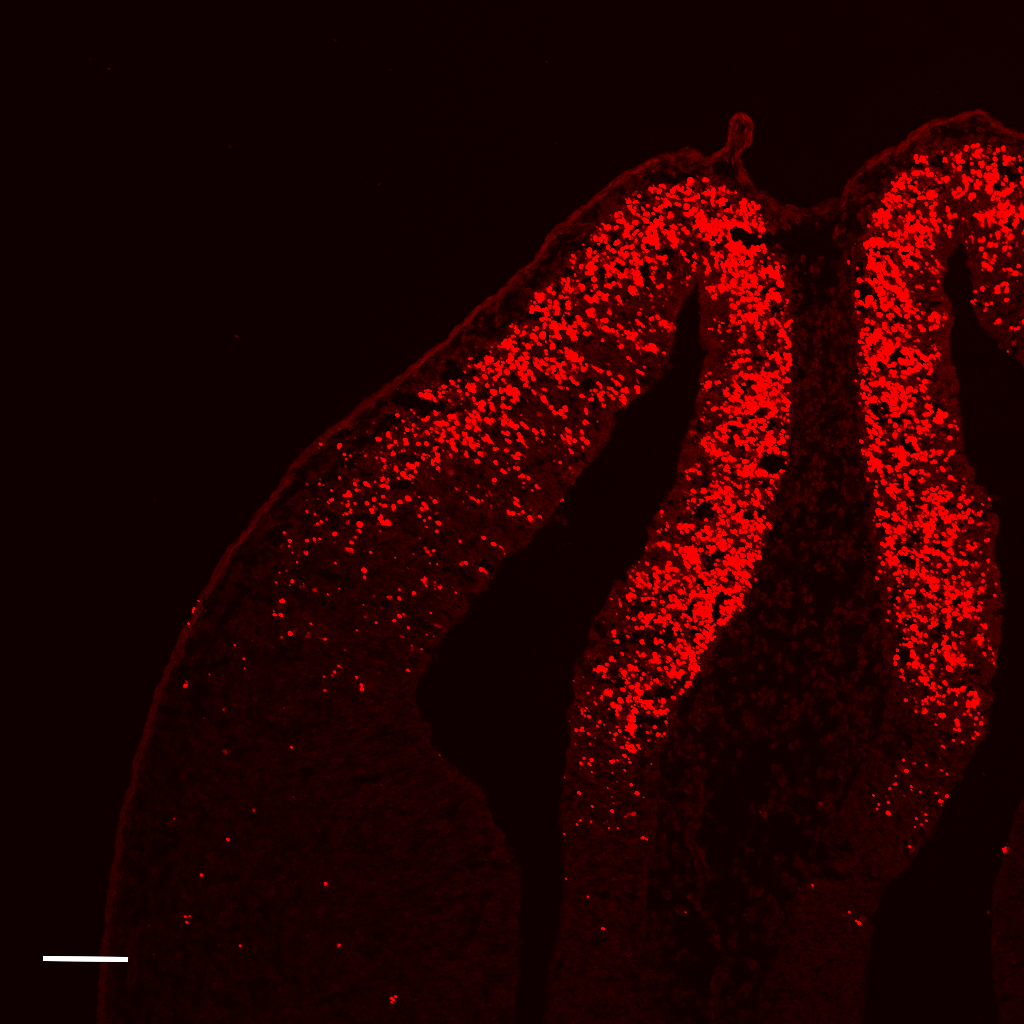

Supplement: Supplementary file 5 — Source data Fig. 3 [file 44319_2026_759_MOESM5_ESM.zip › Source Data for Figure 3/Fig. 3F/e12.5_tut1_ff-emx1-Cre_tunel.tif]

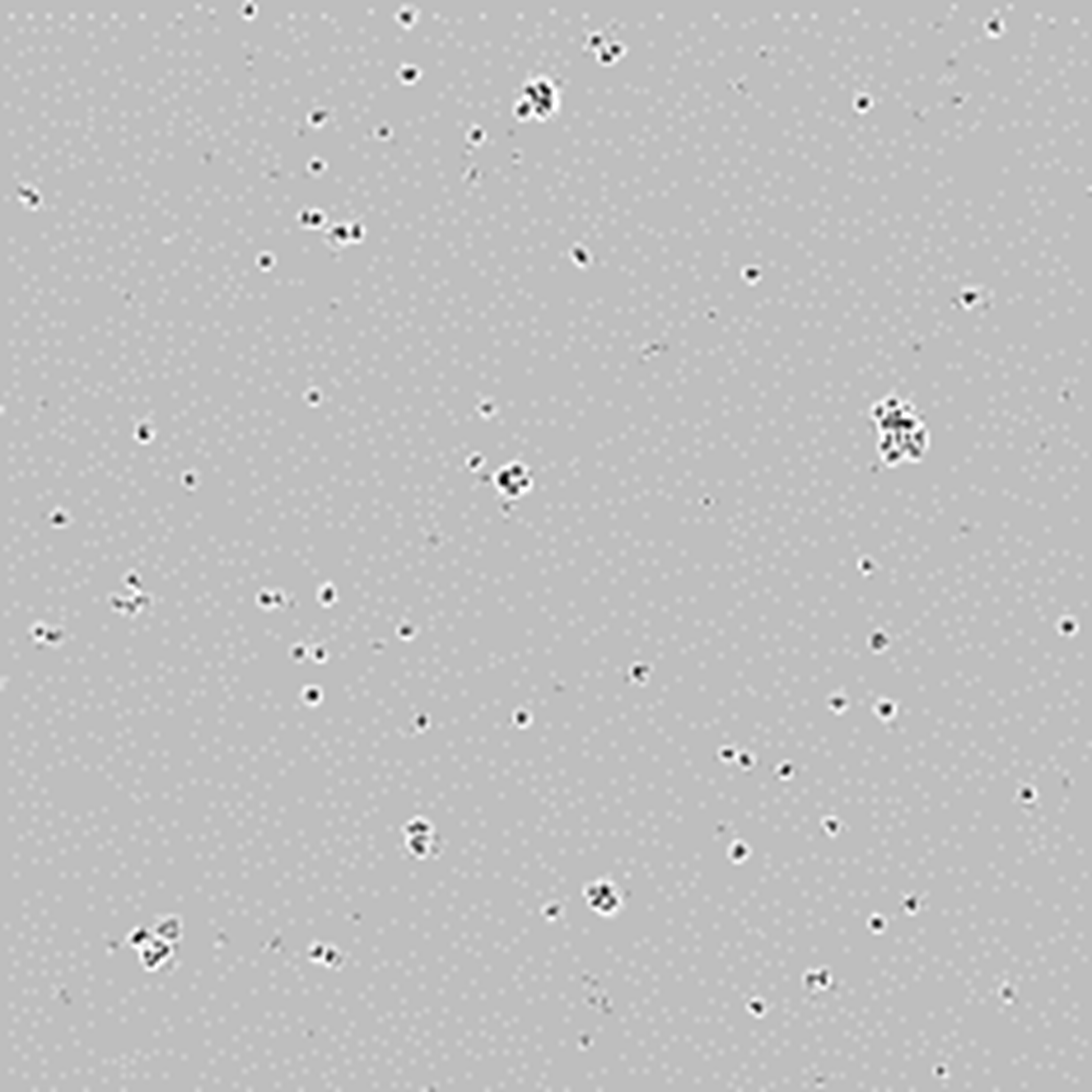

Supplement: Supplementary file 5 — Source data Fig. 3 [file 44319_2026_759_MOESM5_ESM.zip › Source Data for Figure 3/Fig. 3G/Tut1 ff; Emx1-Cre day 1.tif]

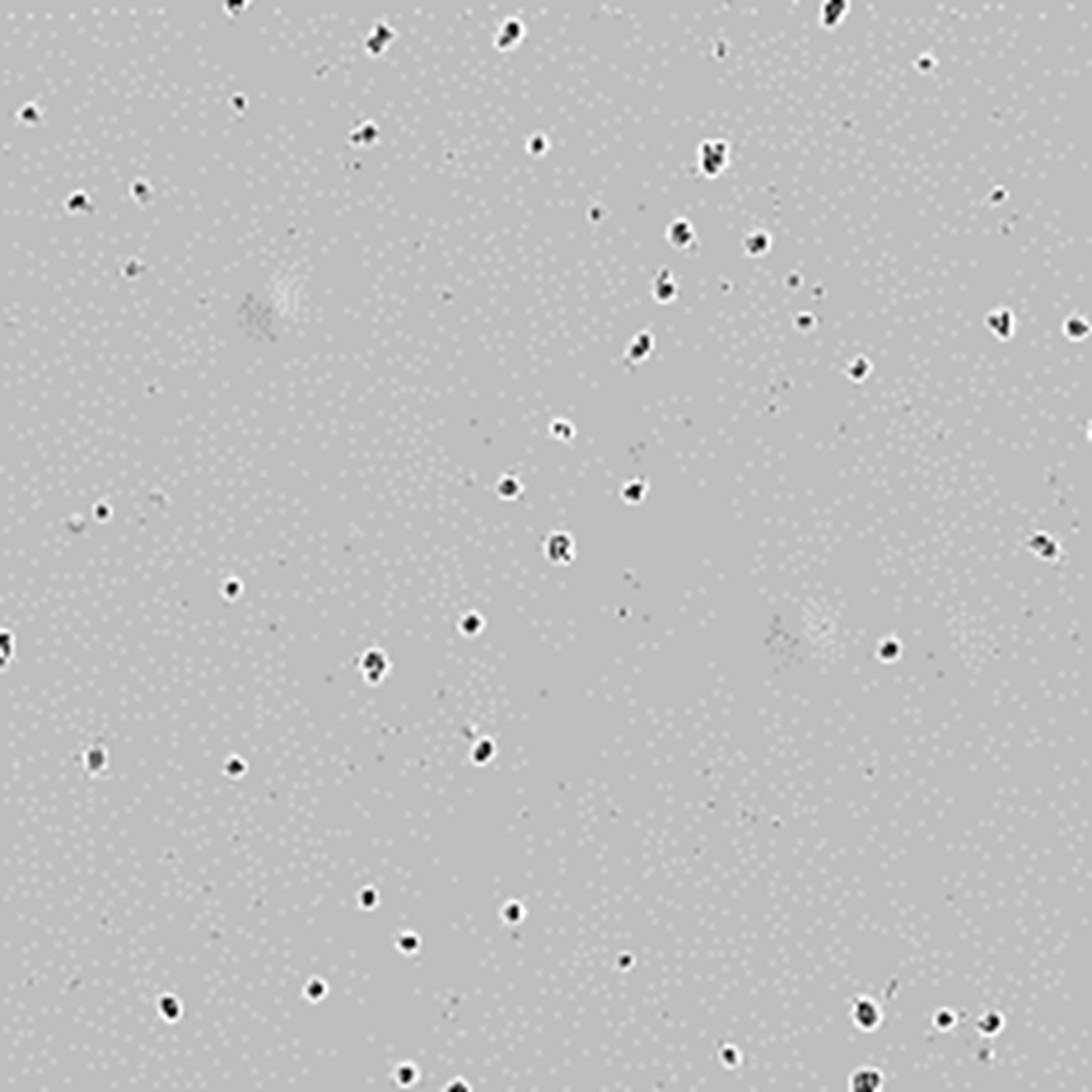

Supplement: Supplementary file 5 — Source data Fig. 3 [file 44319_2026_759_MOESM5_ESM.zip › Source Data for Figure 3/Fig. 3G/Tut1 ff; Emx1-Cre day 4.tif]

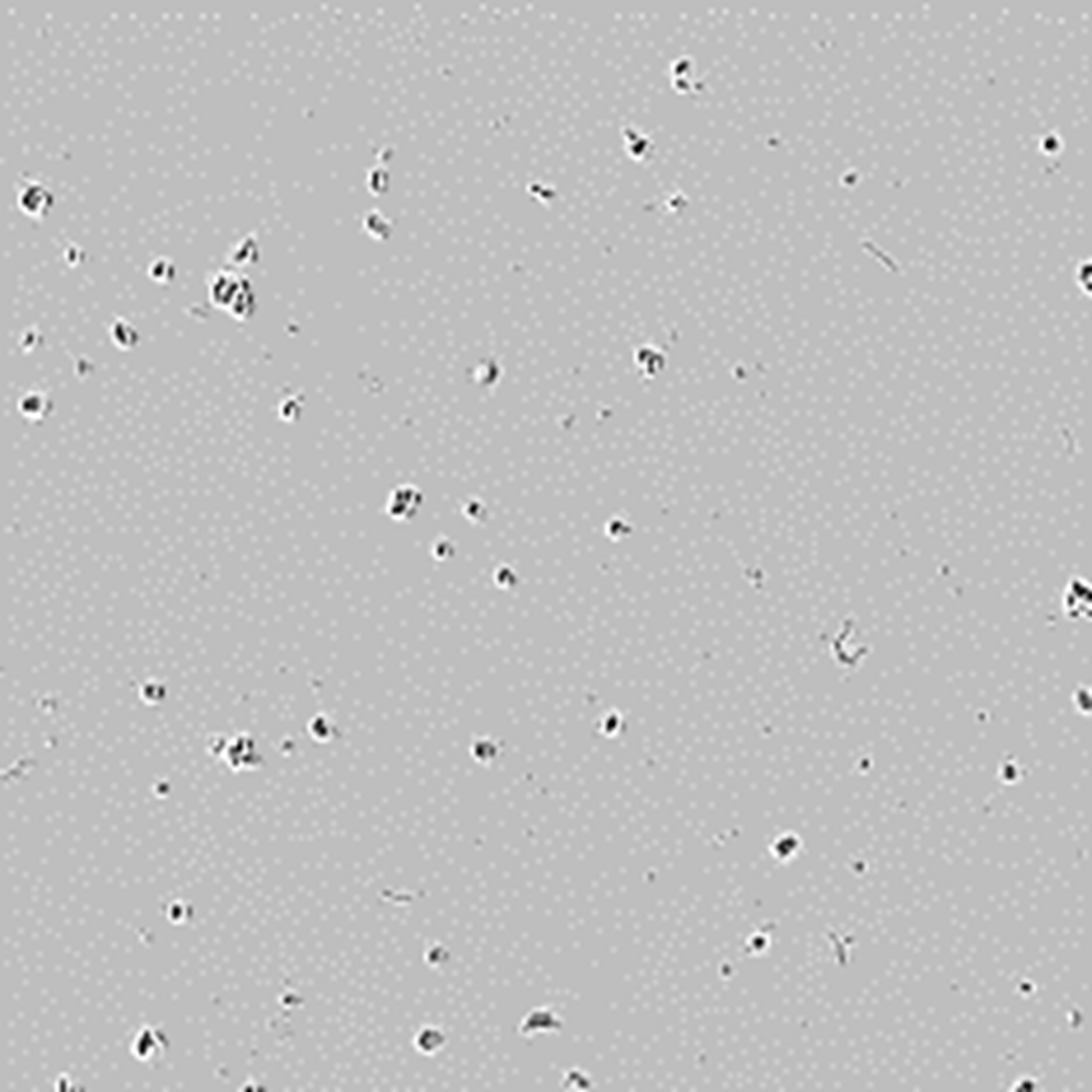

Supplement: Supplementary file 5 — Source data Fig. 3 [file 44319_2026_759_MOESM5_ESM.zip › Source Data for Figure 3/Fig. 3G/WT day 1.tif]

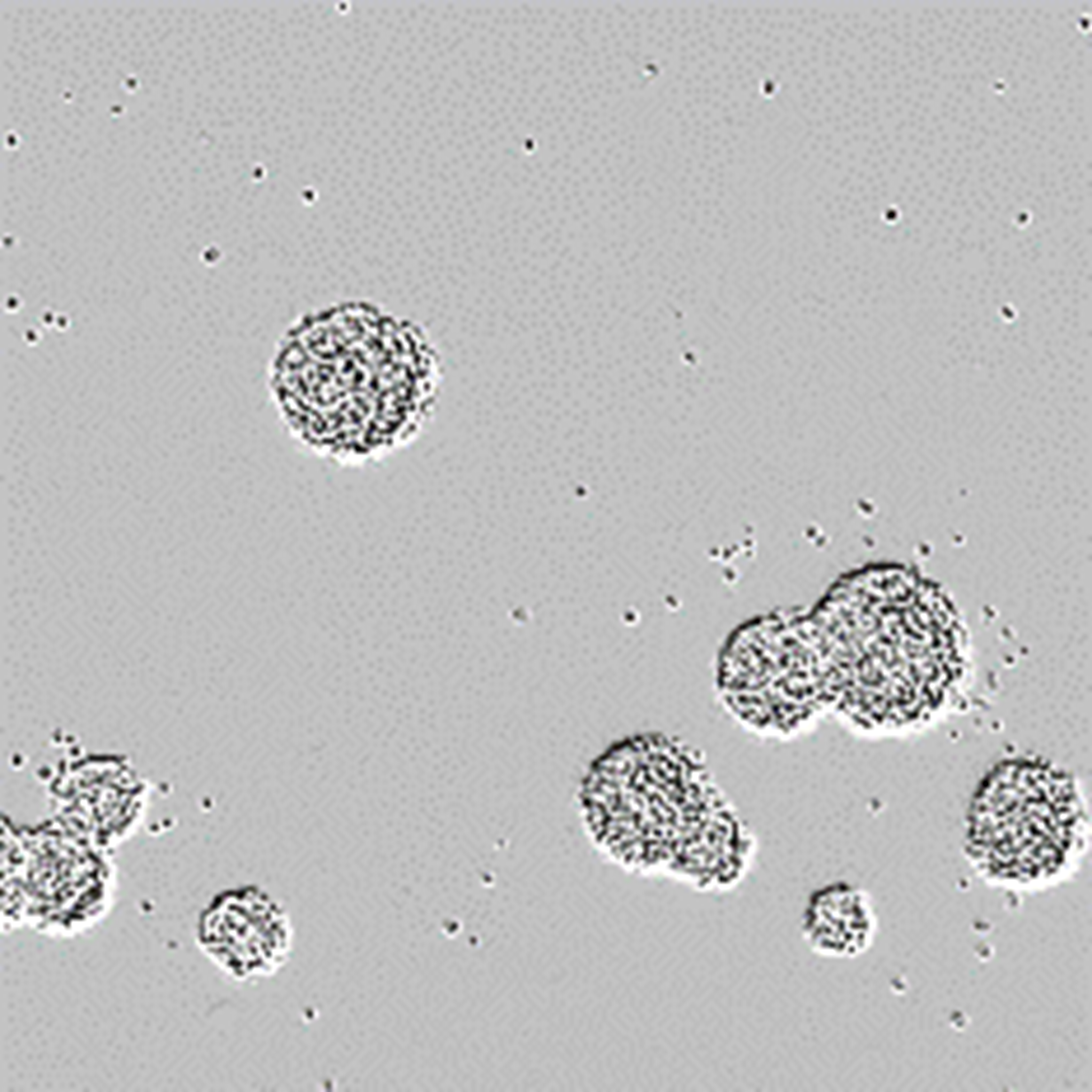

Supplement: Supplementary file 5 — Source data Fig. 3 [file 44319_2026_759_MOESM5_ESM.zip › Source Data for Figure 3/Fig. 3G/WT day 4.tif]

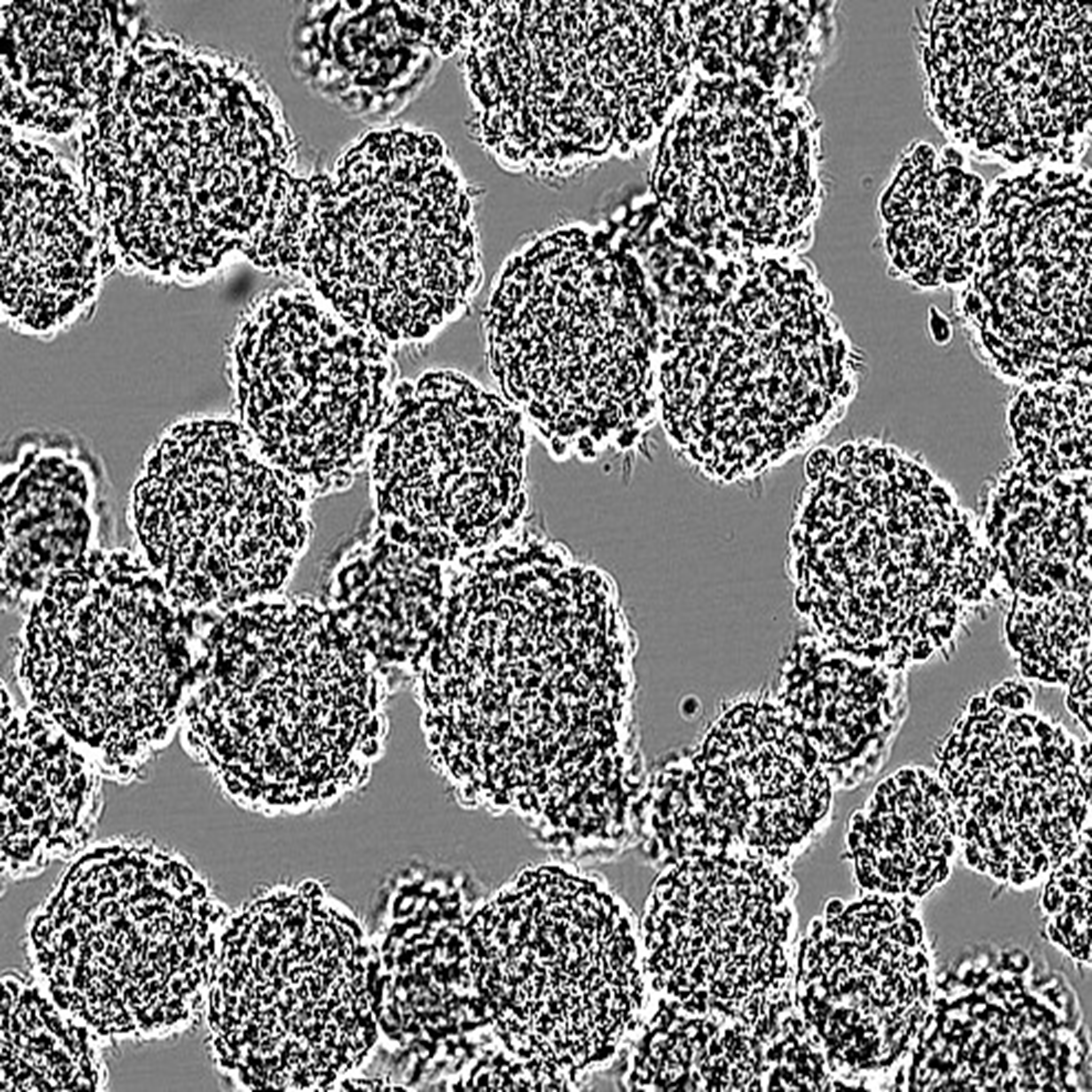

Supplement: Supplementary file 5 — Source data Fig. 3 [file 44319_2026_759_MOESM5_ESM.zip › Source Data for Figure 3/Fig. 3K/Tut1 ff;p53 ff;Emx1-cre.tif]

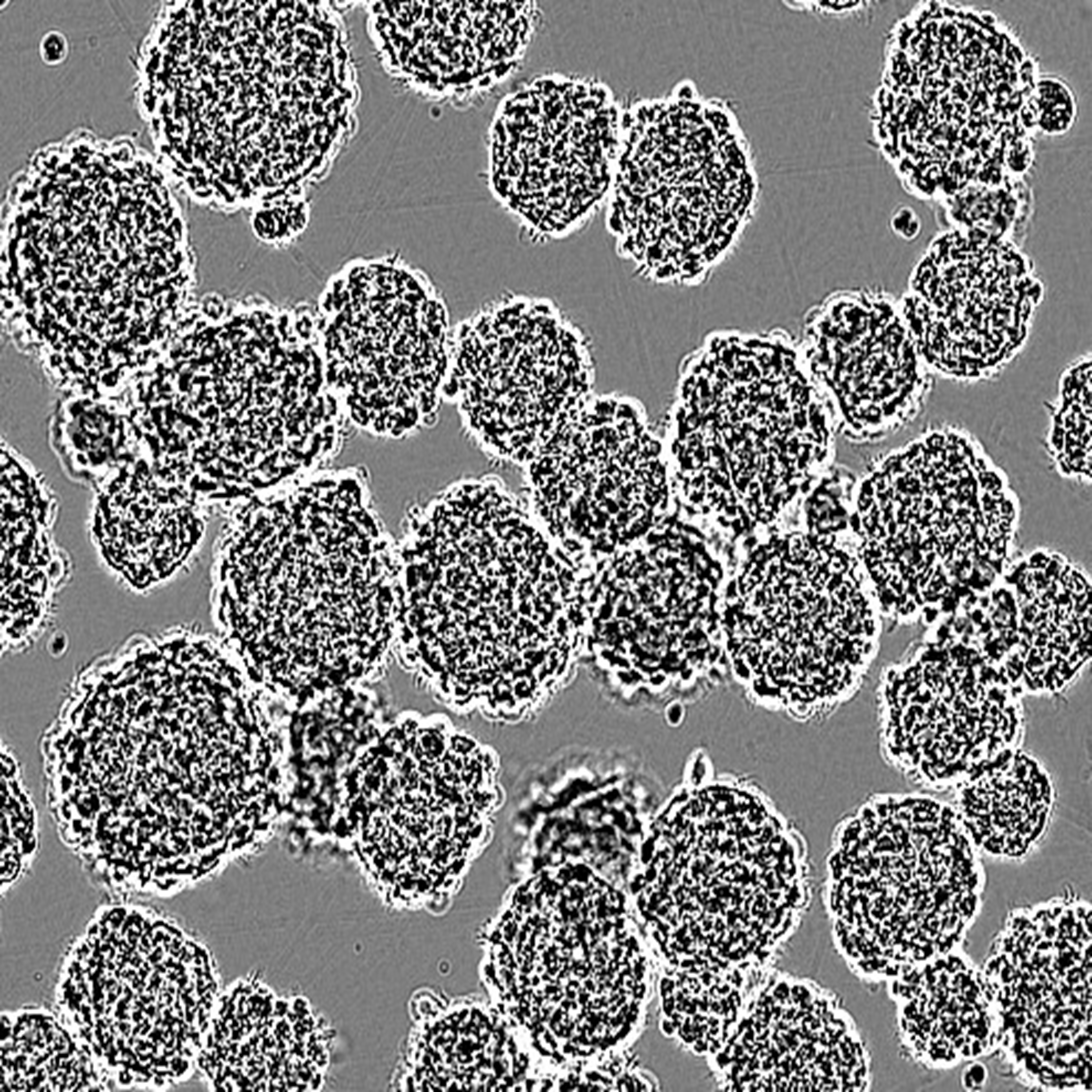

Supplement: Supplementary file 5 — Source data Fig. 3 [file 44319_2026_759_MOESM5_ESM.zip › Source Data for Figure 3/Fig. 3K/WT.tif]

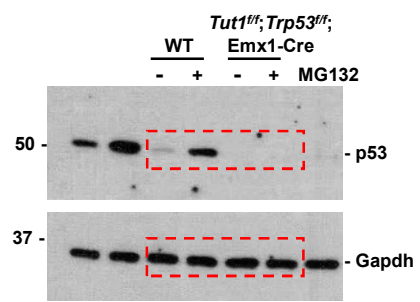

Supplement: Supplementary file 5 — Source data Fig. 3 [file 44319_2026_759_MOESM5_ESM.zip › Source Data for Figure 3/Fig. 3N/western.pdf]

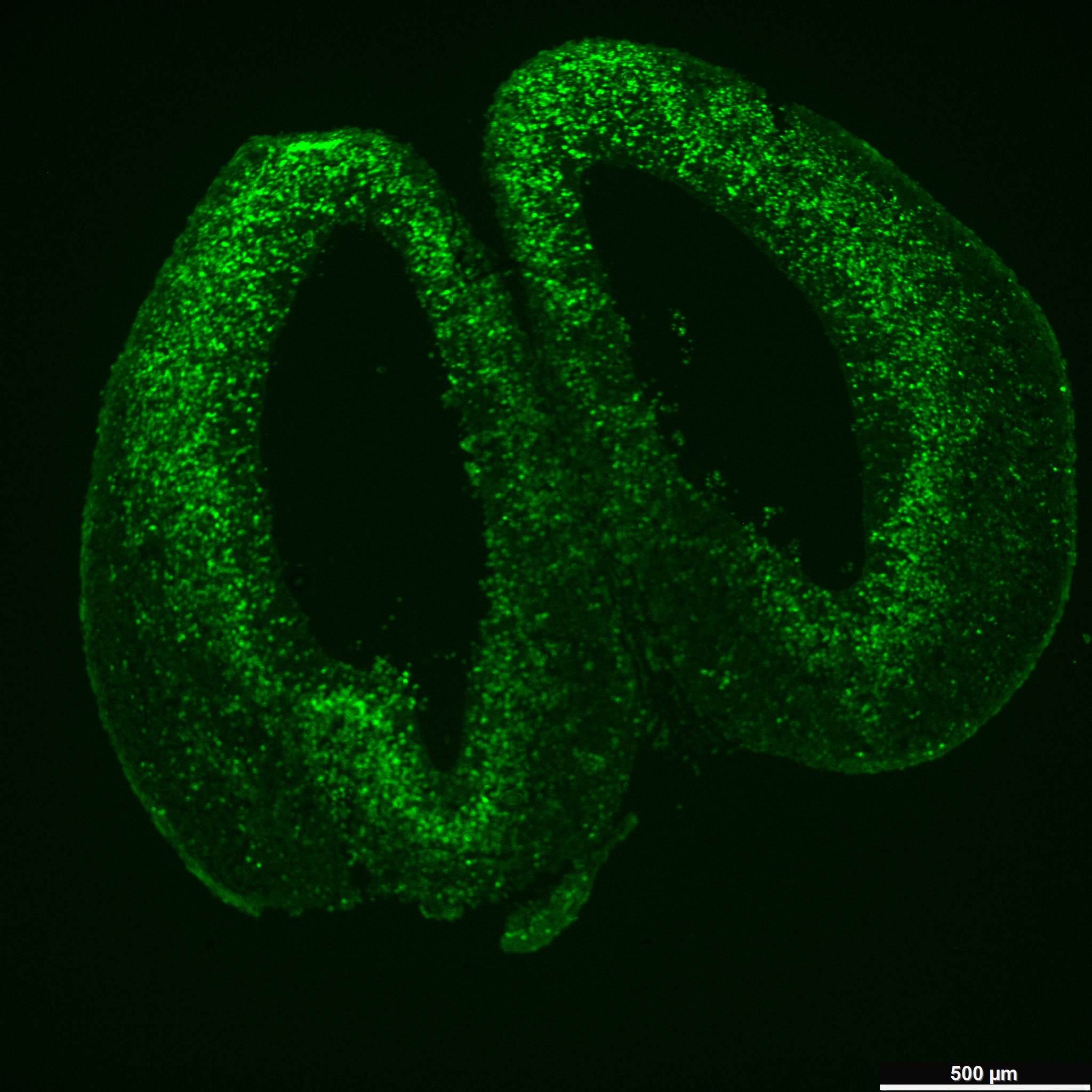

Supplement: Supplementary file 5 — Source data Fig. 3 [file 44319_2026_759_MOESM5_ESM.zip › Source Data for Figure 3/Fig. 3O/Tut1ff;nestin-cre.tif]

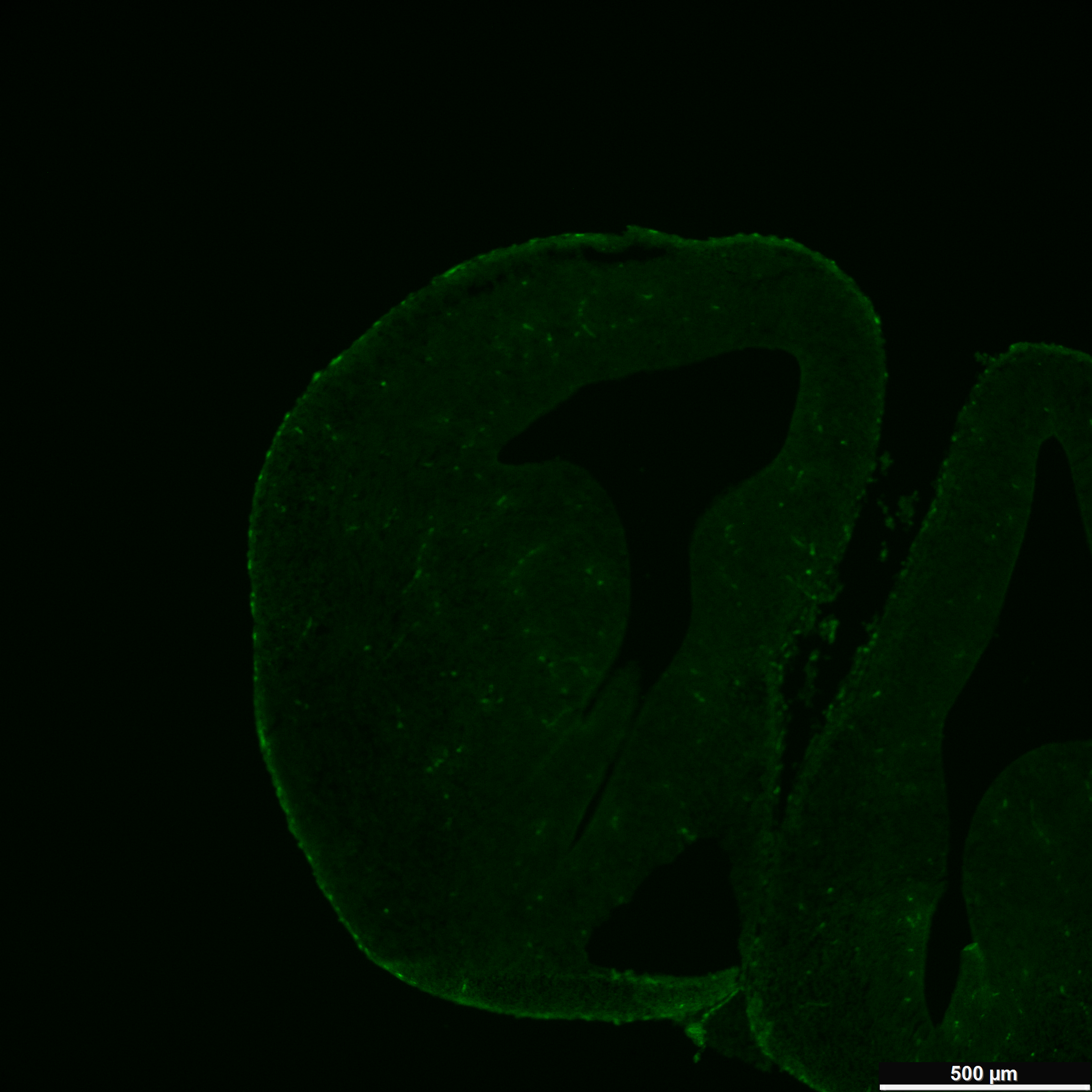

Supplement: Supplementary file 5 — Source data Fig. 3 [file 44319_2026_759_MOESM5_ESM.zip › Source Data for Figure 3/Fig. 3O/Tut1ff;Trp53ff;nestin-cre.tif]

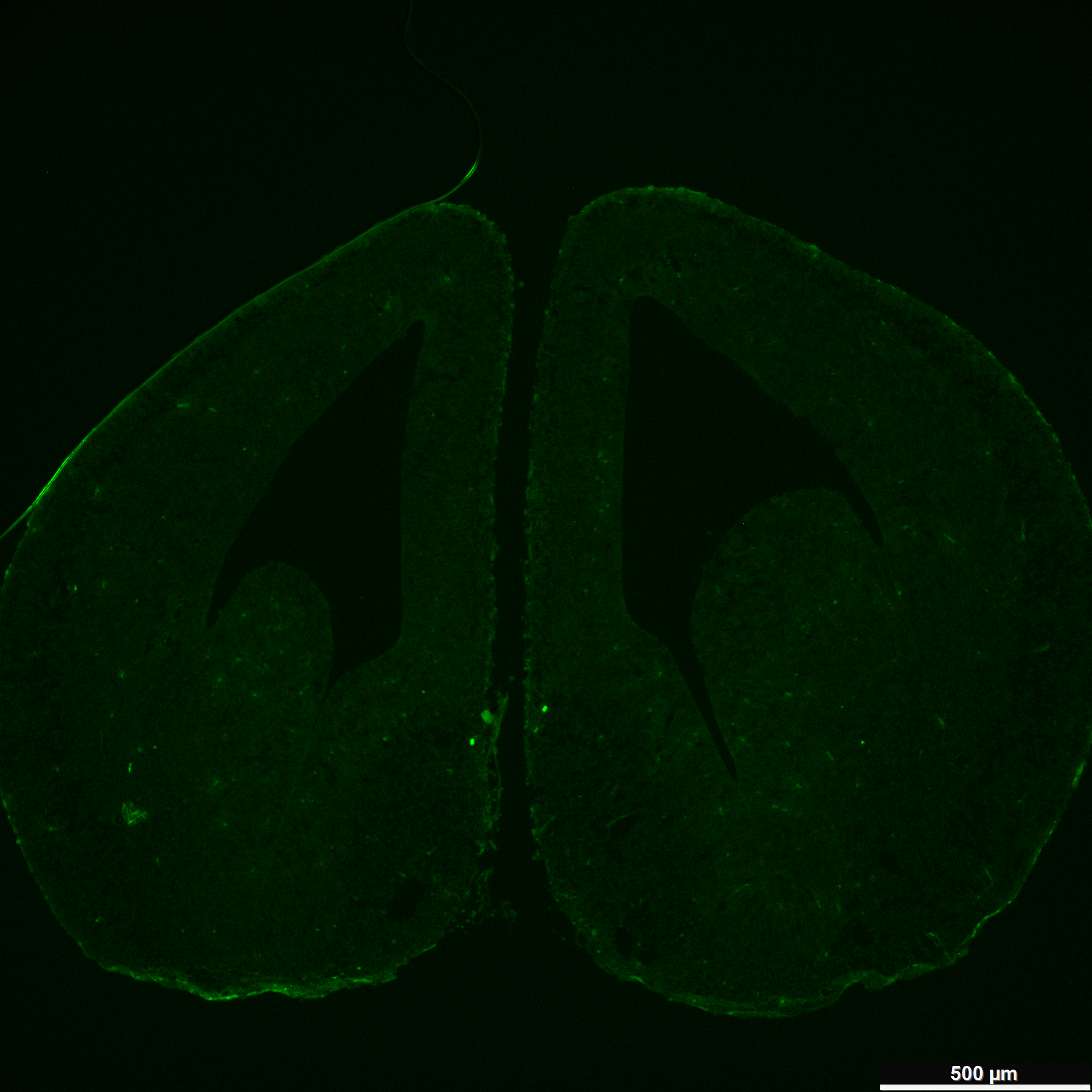

Supplement: Supplementary file 5 — Source data Fig. 3 [file 44319_2026_759_MOESM5_ESM.zip › Source Data for Figure 3/Fig. 3O/WT.tif]

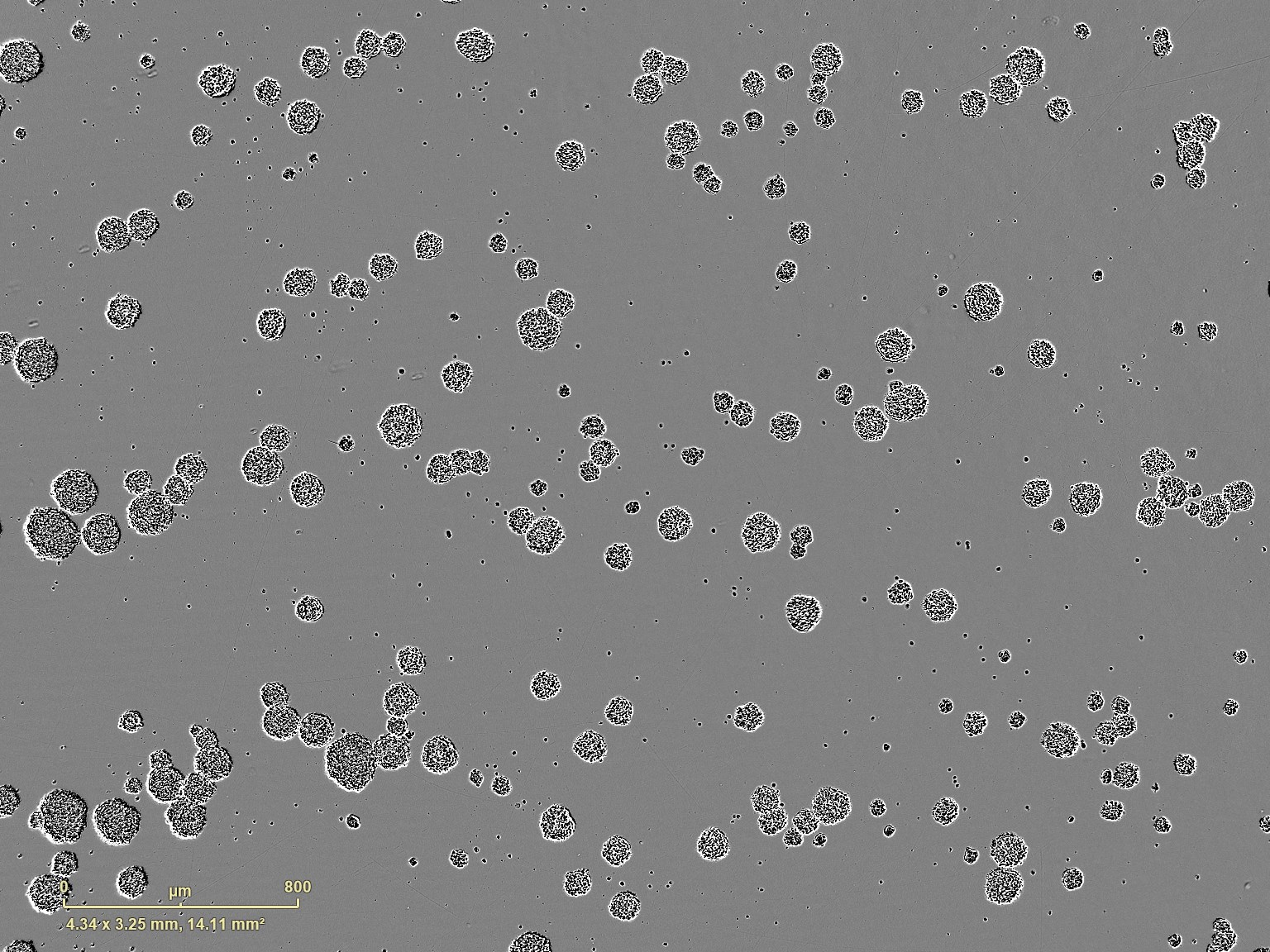

Supplement: Supplementary file 6 — Source data Fig. 4 [file 44319_2026_759_MOESM6_ESM.zip › Source Data for Figure 4/Fig. 4D/Ai14;Cre -.jpg]

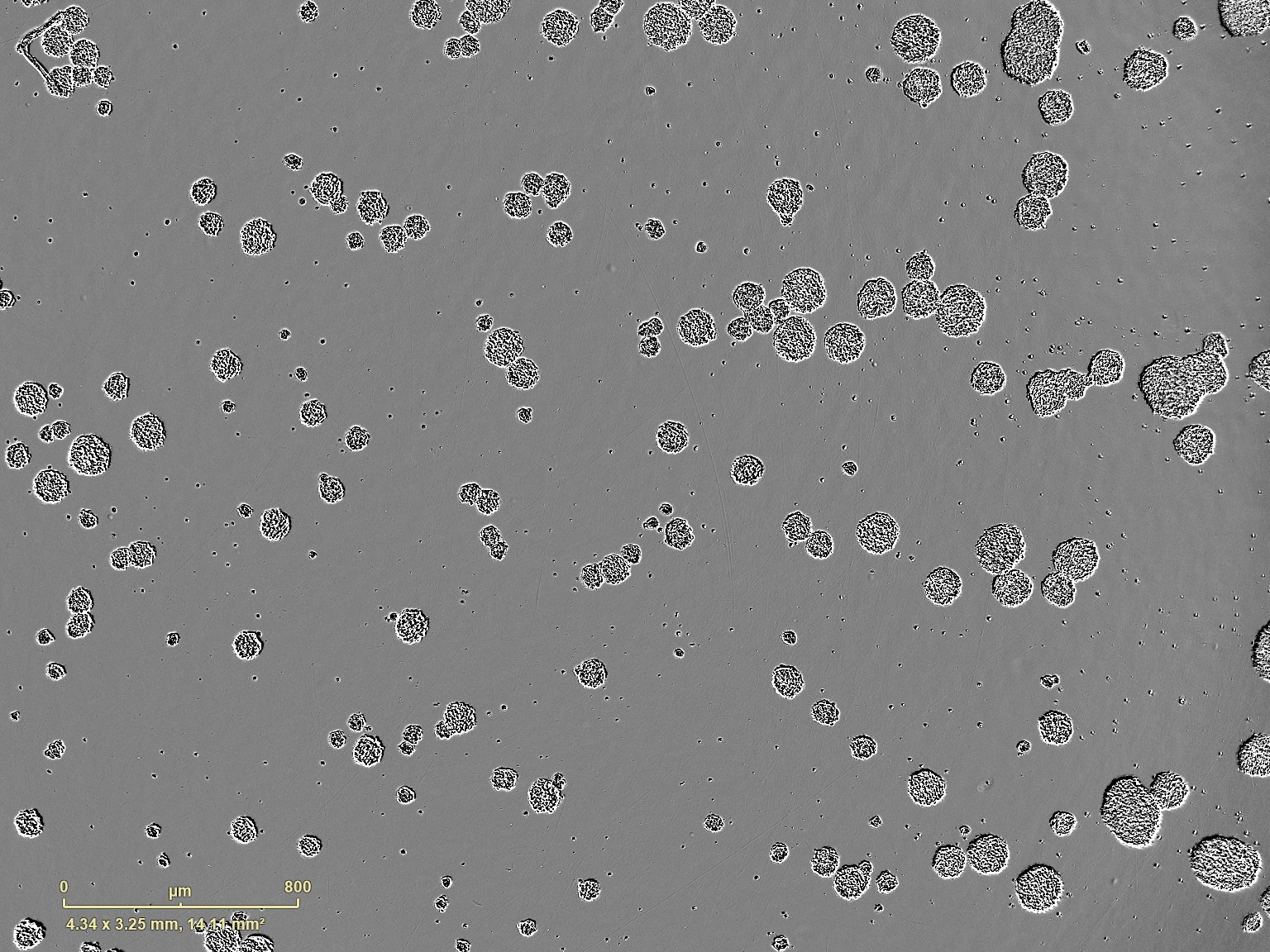

Supplement: Supplementary file 6 — Source data Fig. 4 [file 44319_2026_759_MOESM6_ESM.zip › Source Data for Figure 4/Fig. 4D/Tut1ff.jpg]

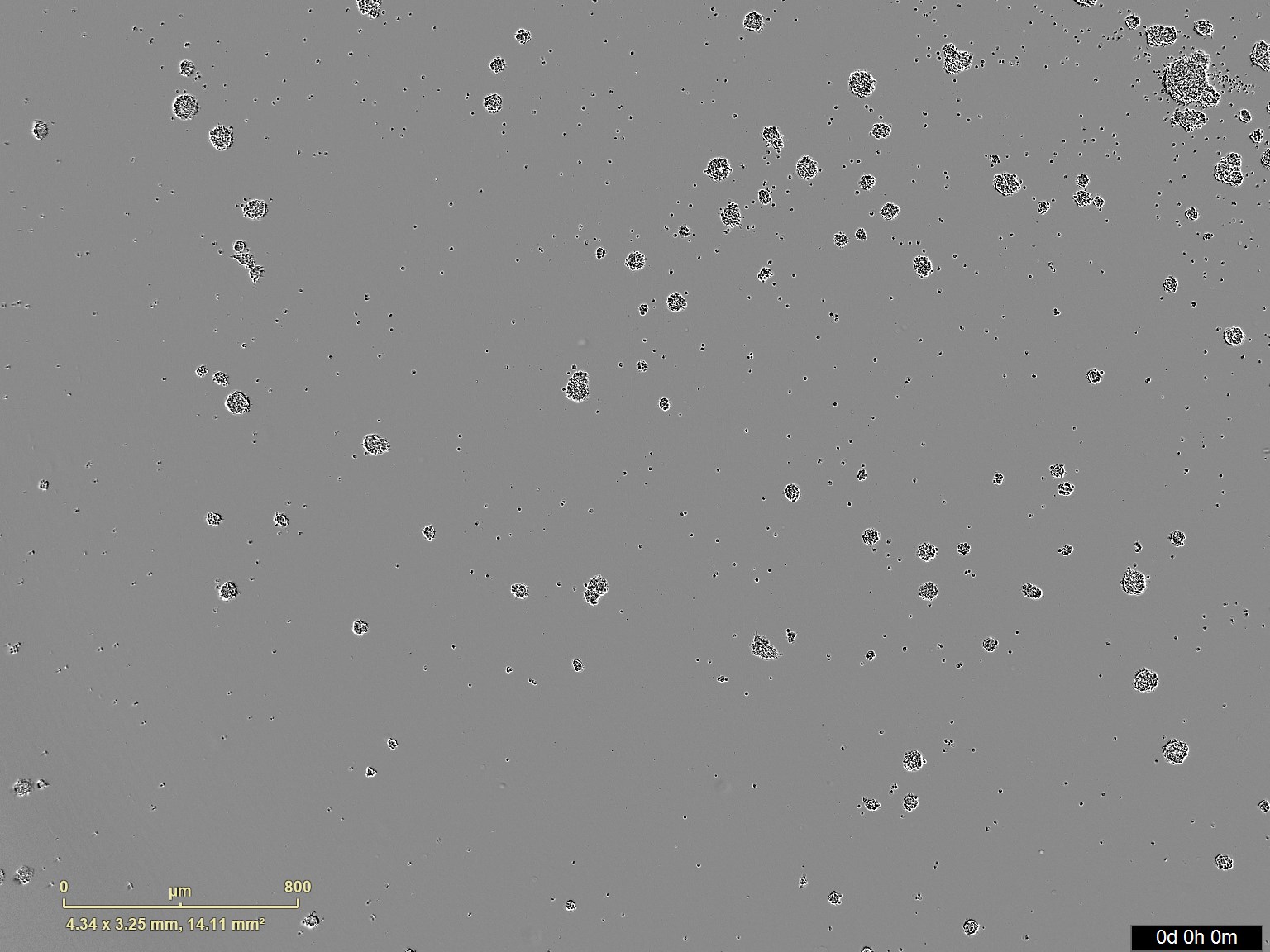

Supplement: Supplementary file 6 — Source data Fig. 4 [file 44319_2026_759_MOESM6_ESM.zip › Source Data for Figure 4/Fig. 4D/Tut1ff;Cre.jpg]

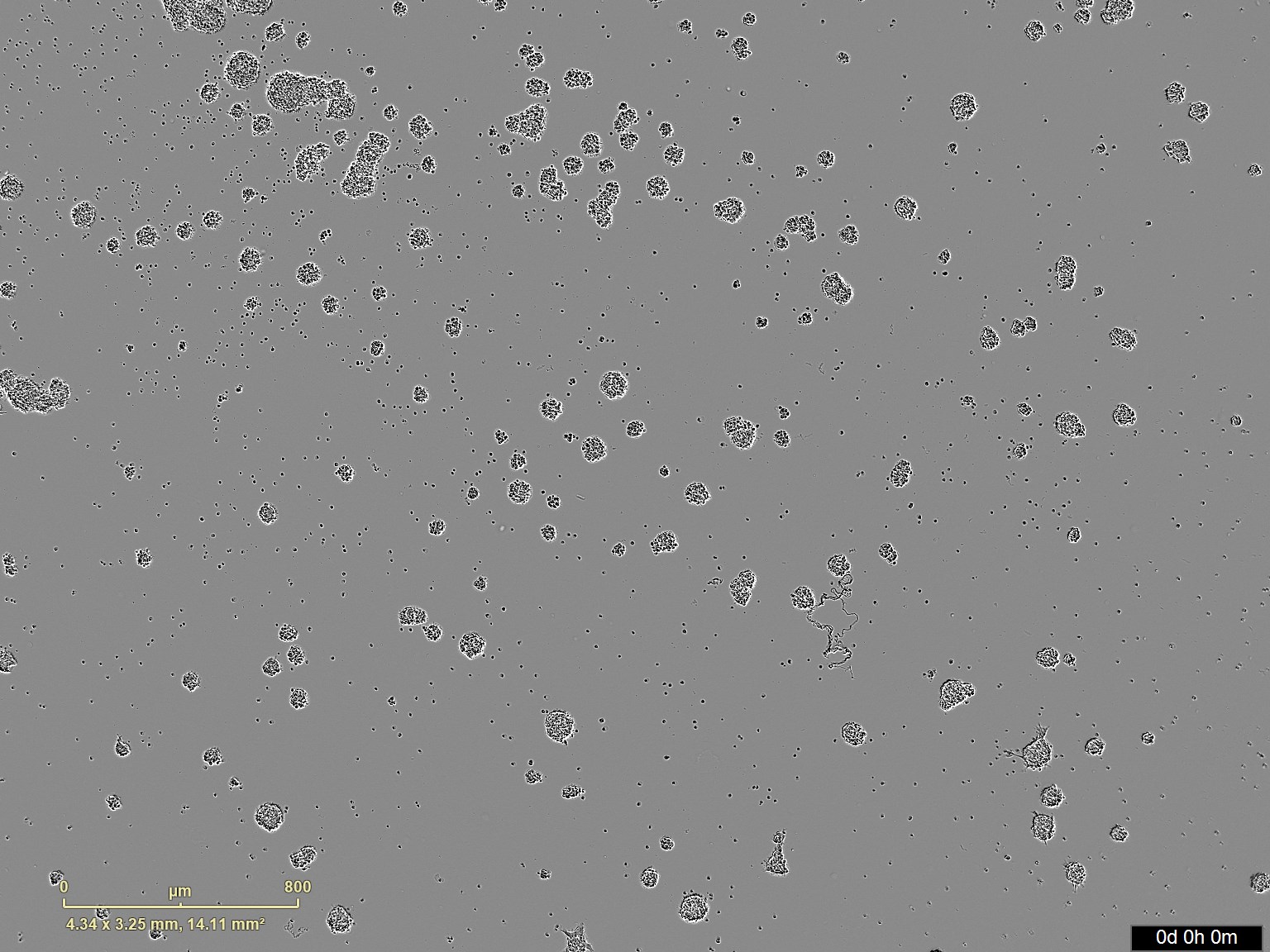

Supplement: Supplementary file 6 — Source data Fig. 4 [file 44319_2026_759_MOESM6_ESM.zip › Source Data for Figure 4/Fig. 4F/0U.jpg]

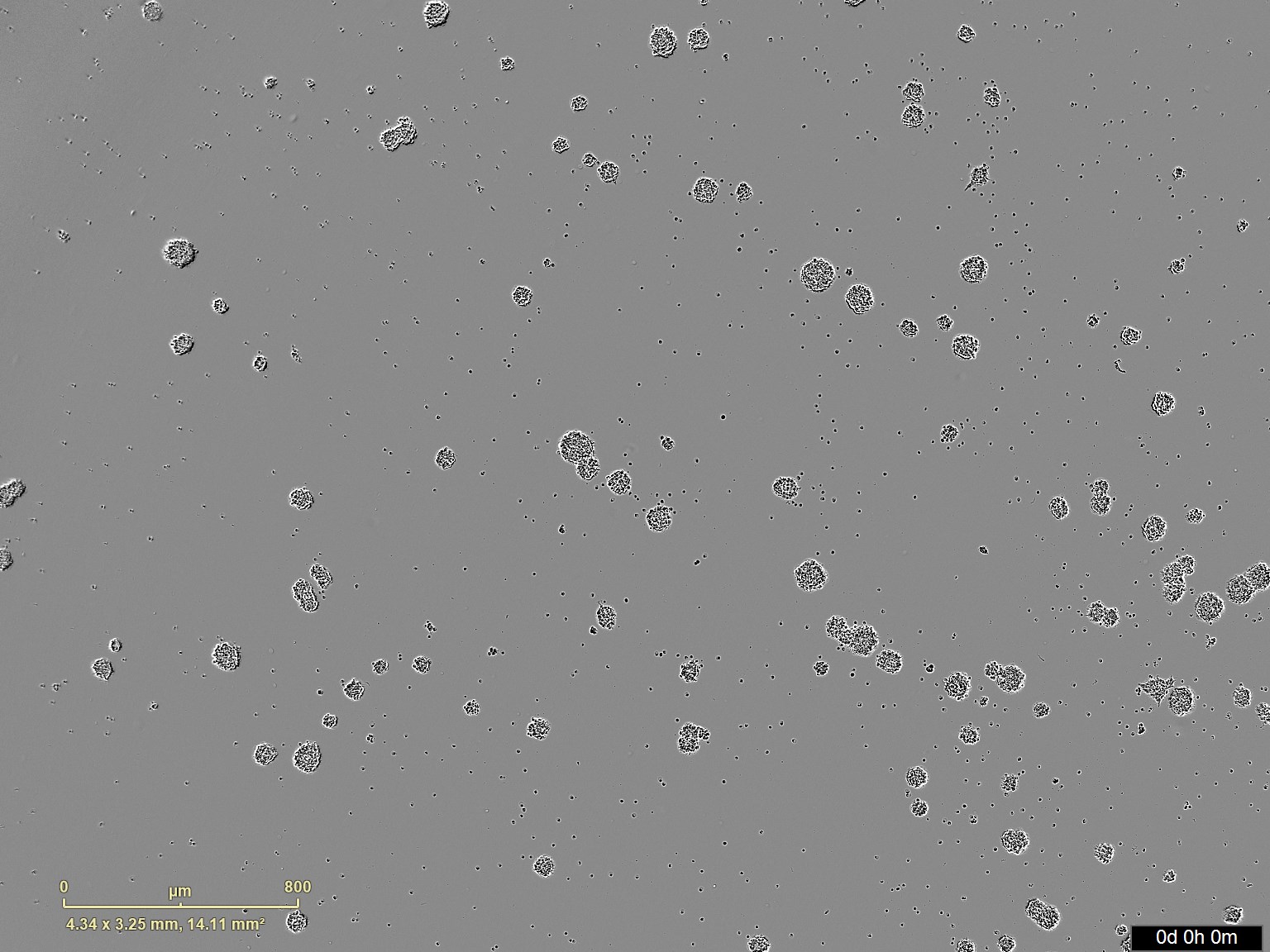

Supplement: Supplementary file 6 — Source data Fig. 4 [file 44319_2026_759_MOESM6_ESM.zip › Source Data for Figure 4/Fig. 4F/2U.jpg]

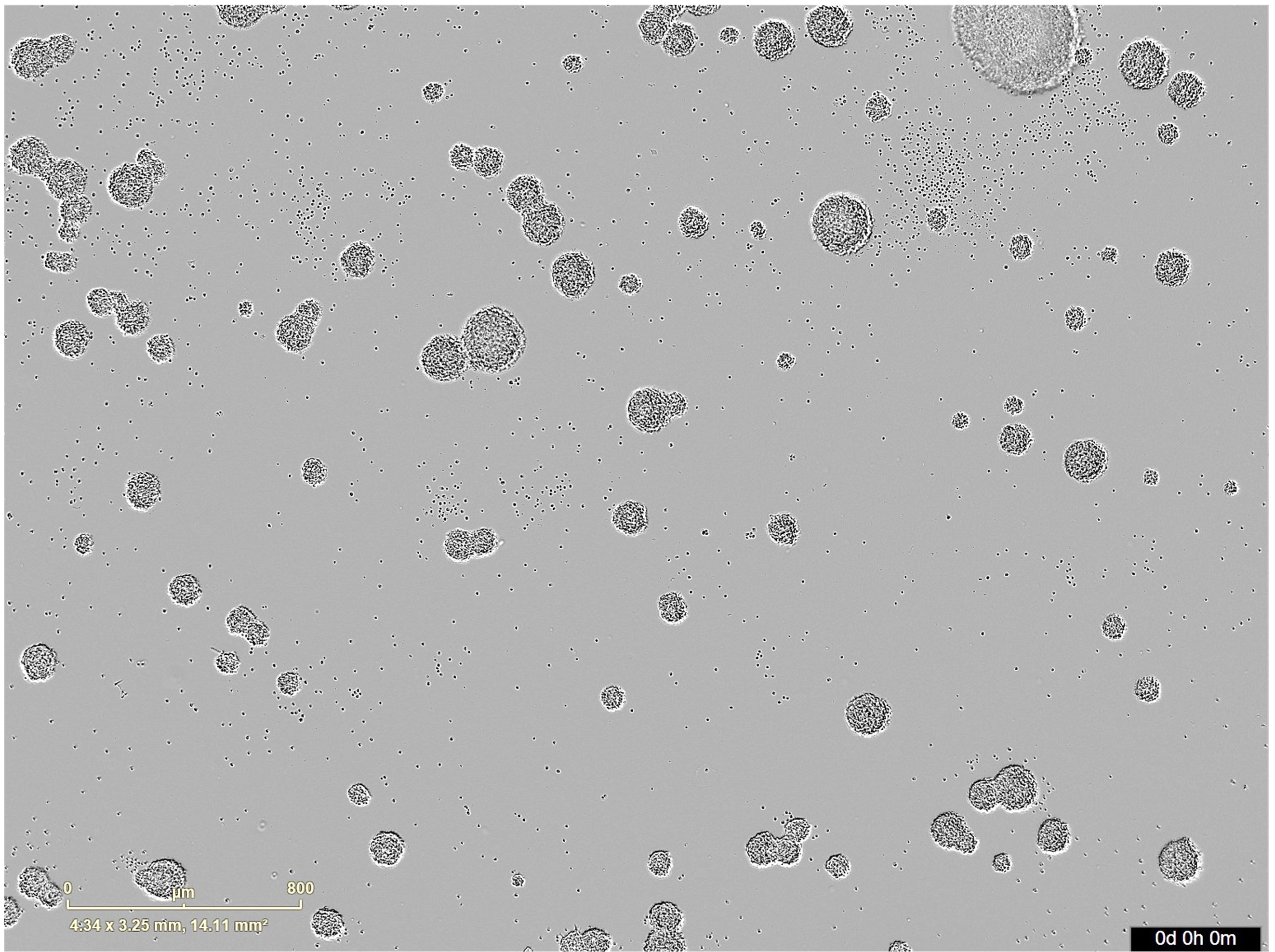

Supplement: Supplementary file 6 — Source data Fig. 4 [file 44319_2026_759_MOESM6_ESM.zip › Source Data for Figure 4/Fig. 4F/4U.jpg]

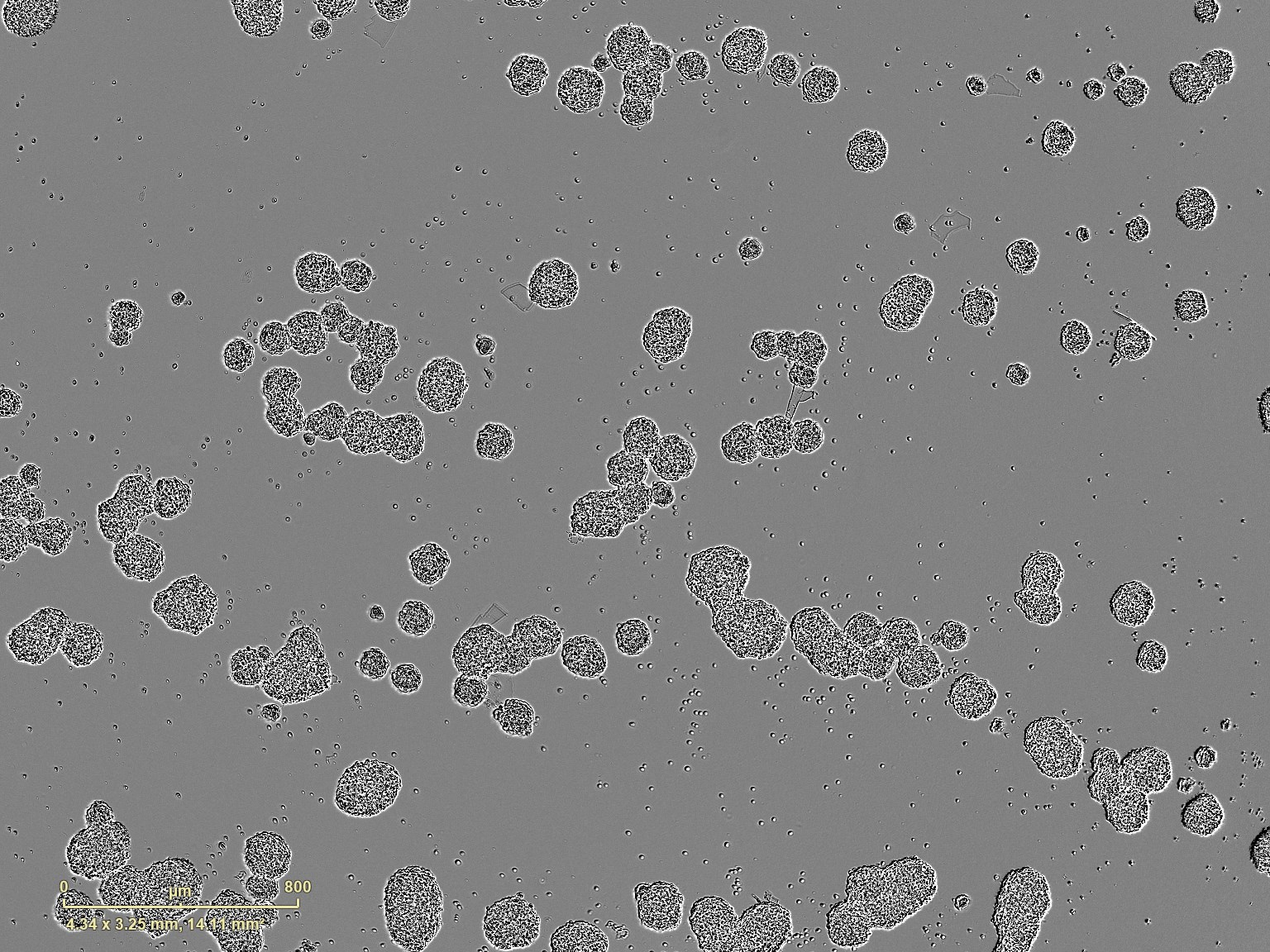

Supplement: Supplementary file 6 — Source data Fig. 4 [file 44319_2026_759_MOESM6_ESM.zip › Source Data for Figure 4/Fig. 4F/6U.jpg]

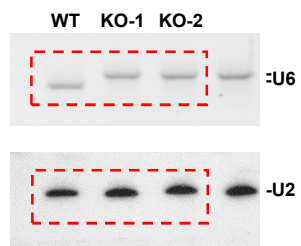

Supplement: Supplementary file 8 — Source data Fig. 6 [file 44319_2026_759_MOESM8_ESM.zip › Source Data for Figure 6/Fig. 6B/northern.pdf]

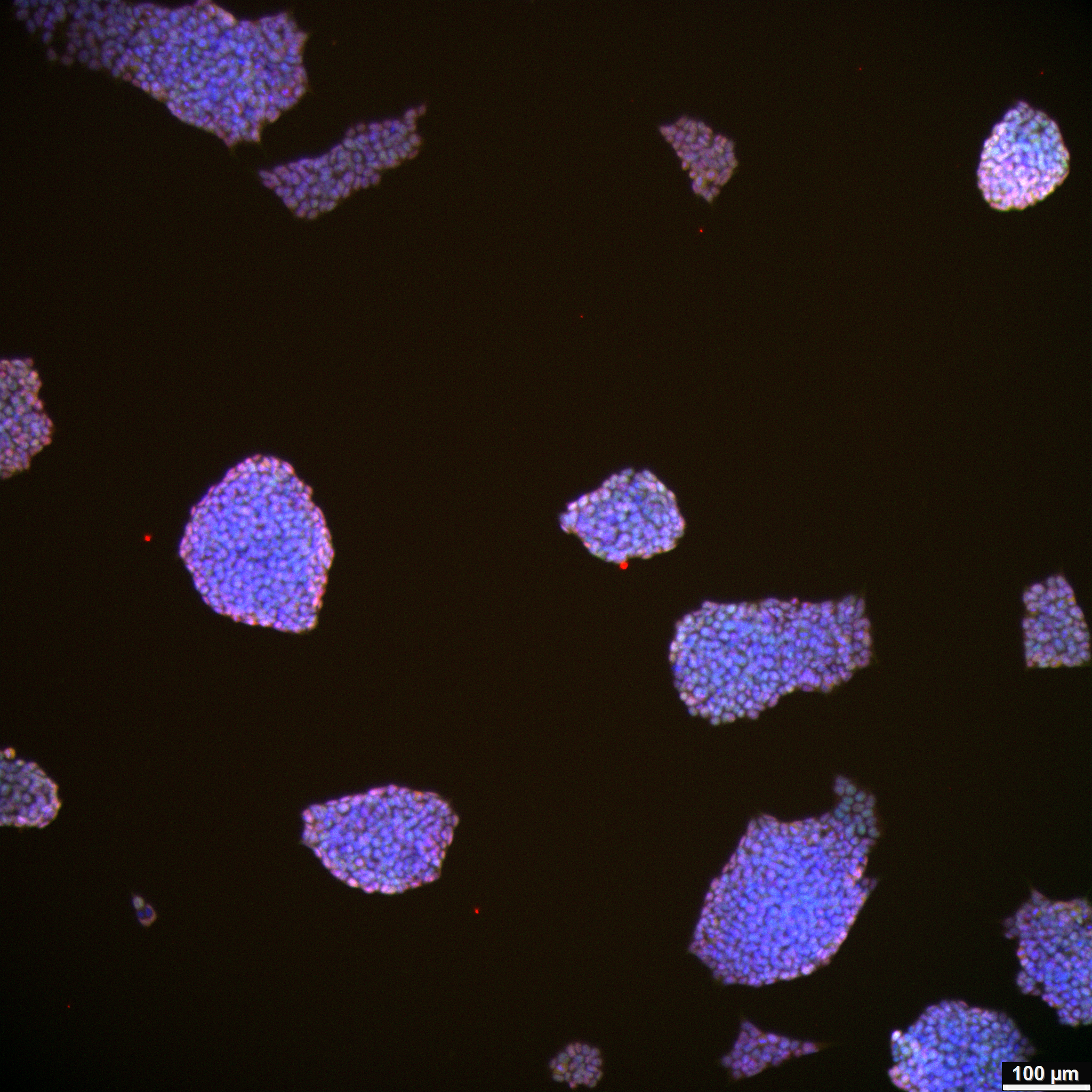

Supplement: Supplementary file 8 — Source data Fig. 6 [file 44319_2026_759_MOESM8_ESM.zip › Source Data for Figure 6/Fig. 6C/uko dapi oct4 esrrb.tif]

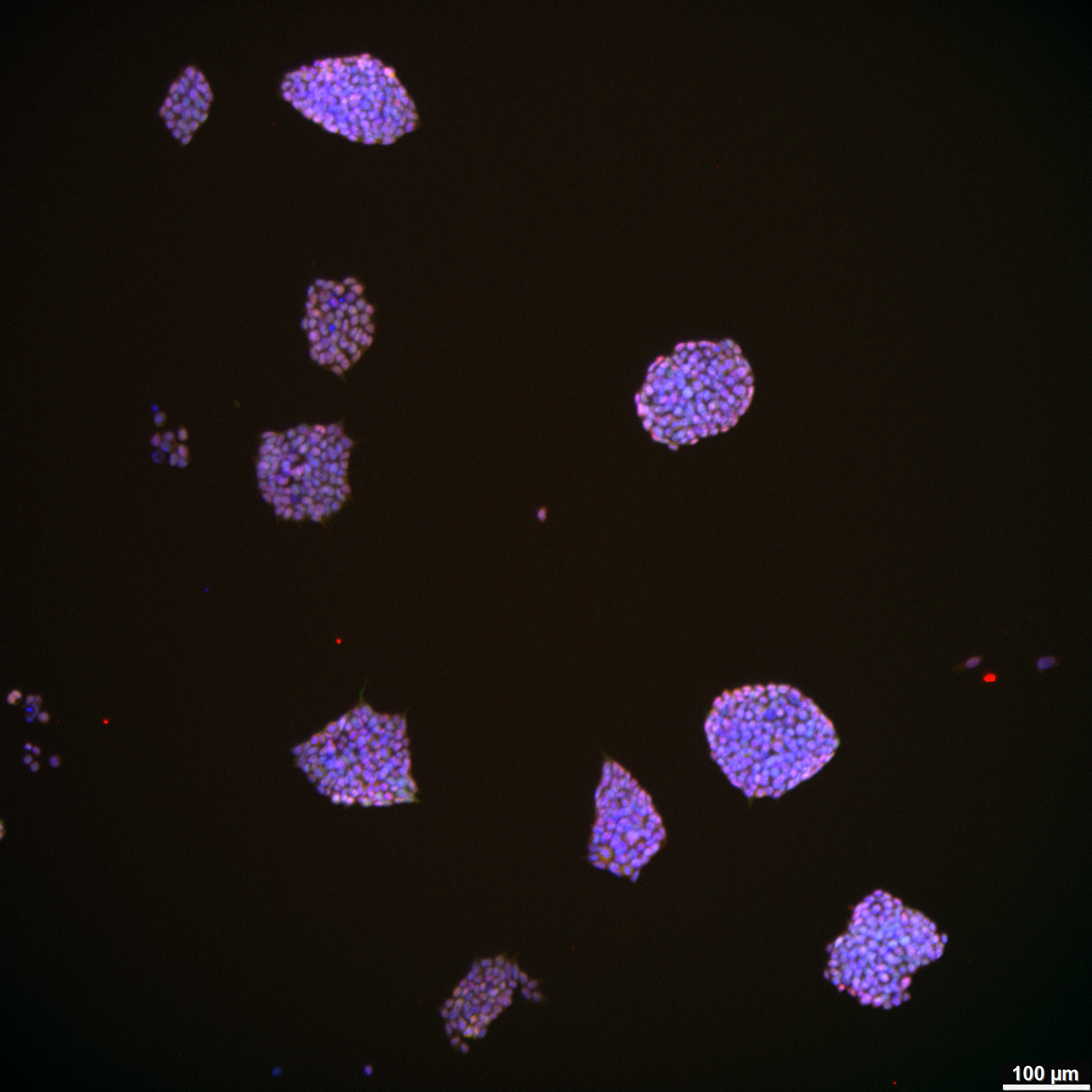

Supplement: Supplementary file 8 — Source data Fig. 6 [file 44319_2026_759_MOESM8_ESM.zip › Source Data for Figure 6/Fig. 6C/wt dapi oct4 esrrb.tif]

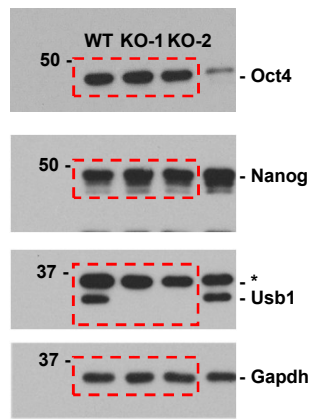

Supplement: Supplementary file 8 — Source data Fig. 6 [file 44319_2026_759_MOESM8_ESM.zip › Source Data for Figure 6/Fig. 6D/werstern.pdf]

E15.5 dorsal pallium

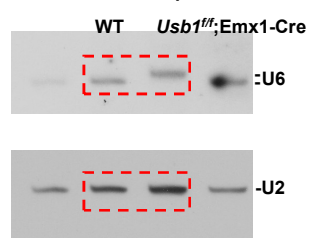

Supplement: Supplementary file 9 — Source data Fig. 7 [file 44319_2026_759_MOESM9_ESM.zip › Source Data for Figure 7/Fig. 7D/northern.pdf]

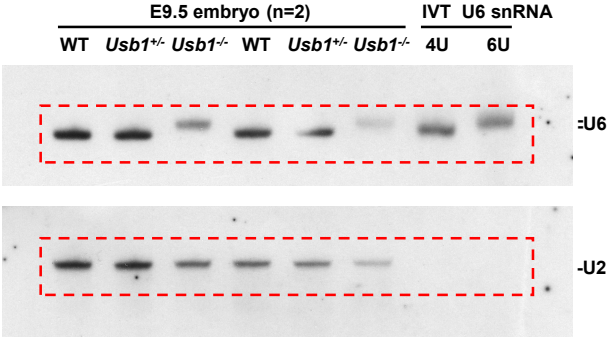

Supplement: Supplementary file 9 — Source data Fig. 7 [file 44319_2026_759_MOESM9_ESM.zip › Source Data for Figure 7/Fig. 7J/northern.pdf]

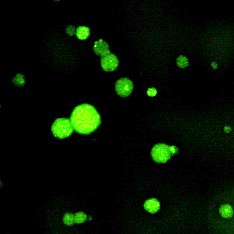

Supplement: Supplementary file 11 — Figure EV2 Source Data [file 44319_2026_759_MOESM11_ESM.zip › Source Data for Fig. EV2/Fig. EV2A/Neural stem cell with LNPGFP-mRNA.tif]

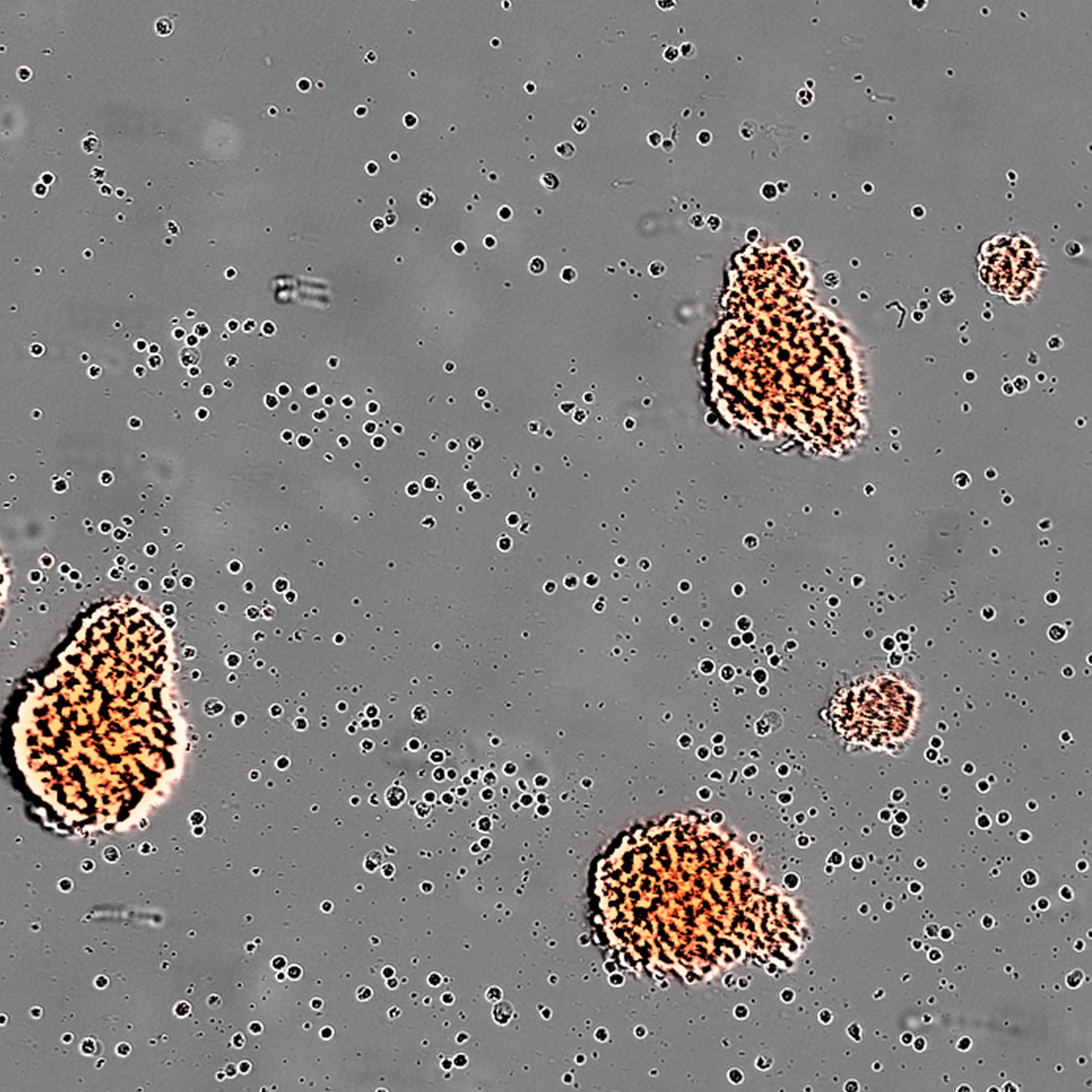

Supplement: Supplementary file 11 — Figure EV2 Source Data [file 44319_2026_759_MOESM11_ESM.zip › Source Data for Fig. EV2/Fig. EV2C/LNP Cre-mRNA.tif]

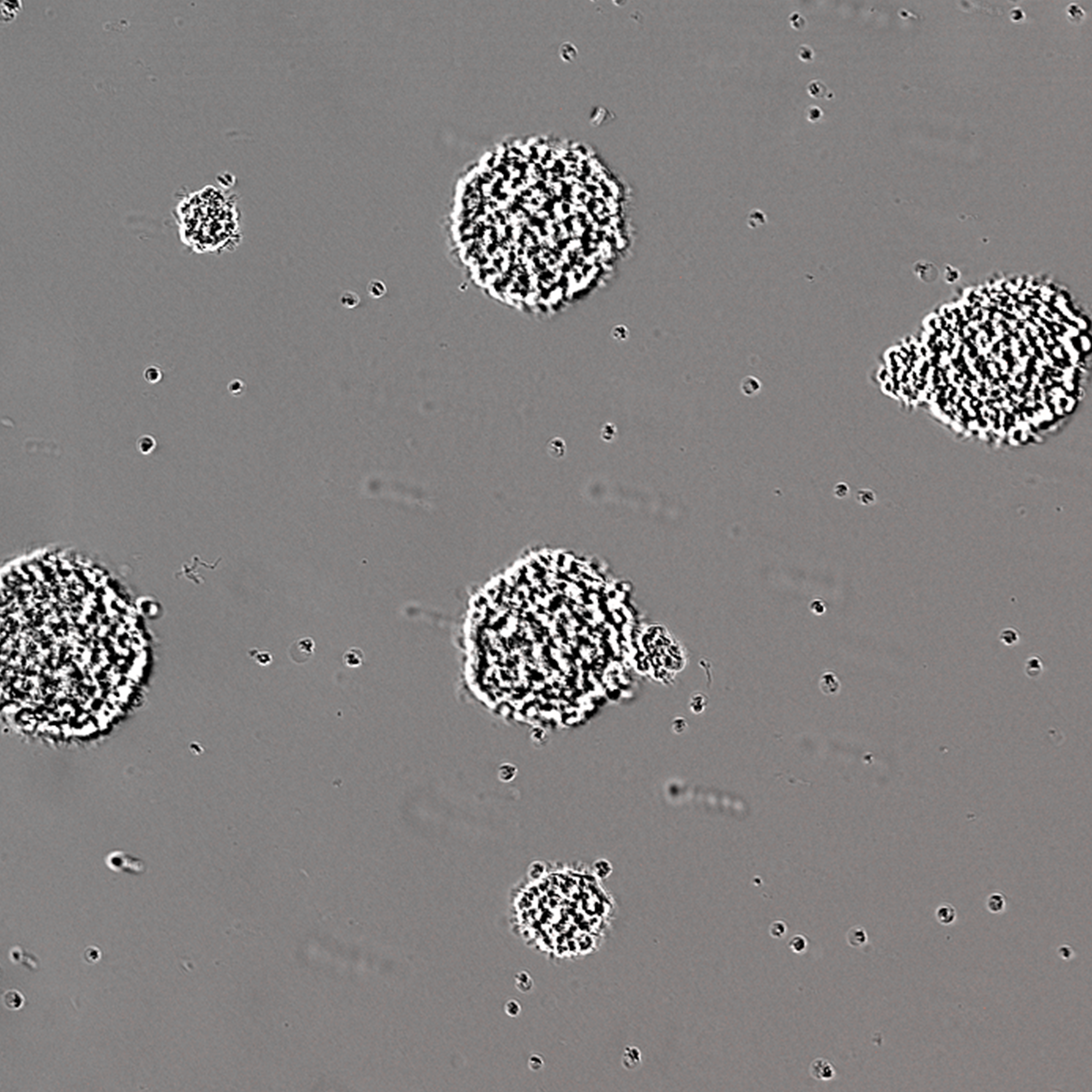

Supplement: Supplementary file 11 — Figure EV2 Source Data [file 44319_2026_759_MOESM11_ESM.zip › Source Data for Fig. EV2/Fig. EV2C/LNP.tif]

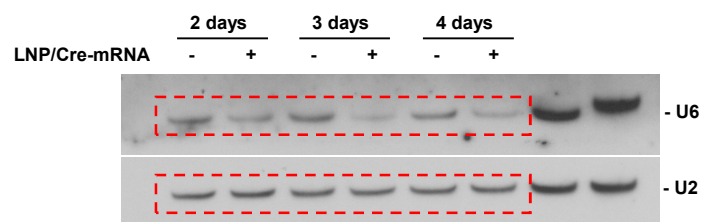

Supplement: Supplementary file 11 — Figure EV2 Source Data [file 44319_2026_759_MOESM11_ESM.zip › Source Data for Fig. EV2/Fig. EV2D/northern.pdf]

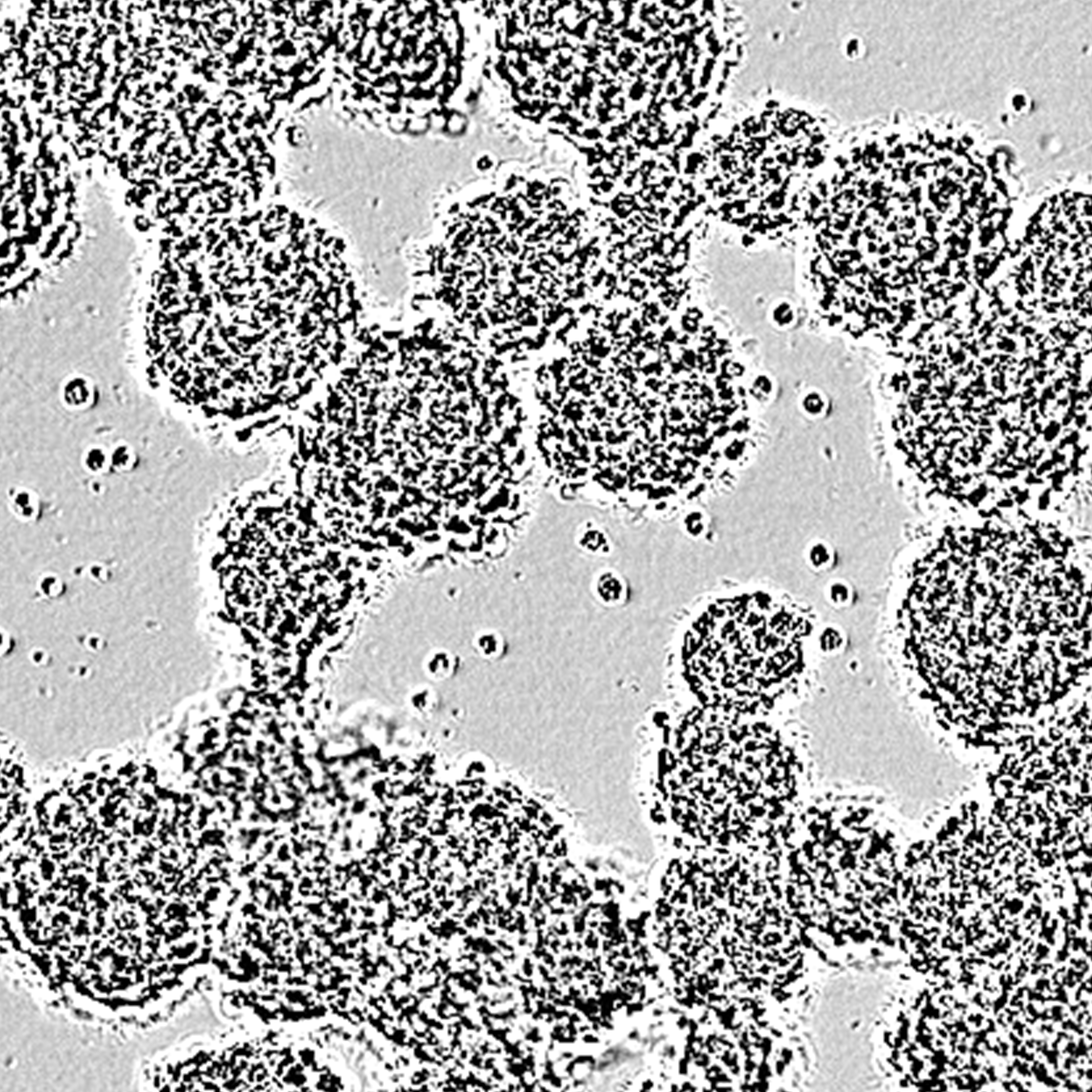

Supplement: Supplementary file 12 — Figure EV4 Source Data [file 44319_2026_759_MOESM12_ESM.zip › Source Data for Fig. EV4/Fig. EV4B/Usb1 ff.tif]

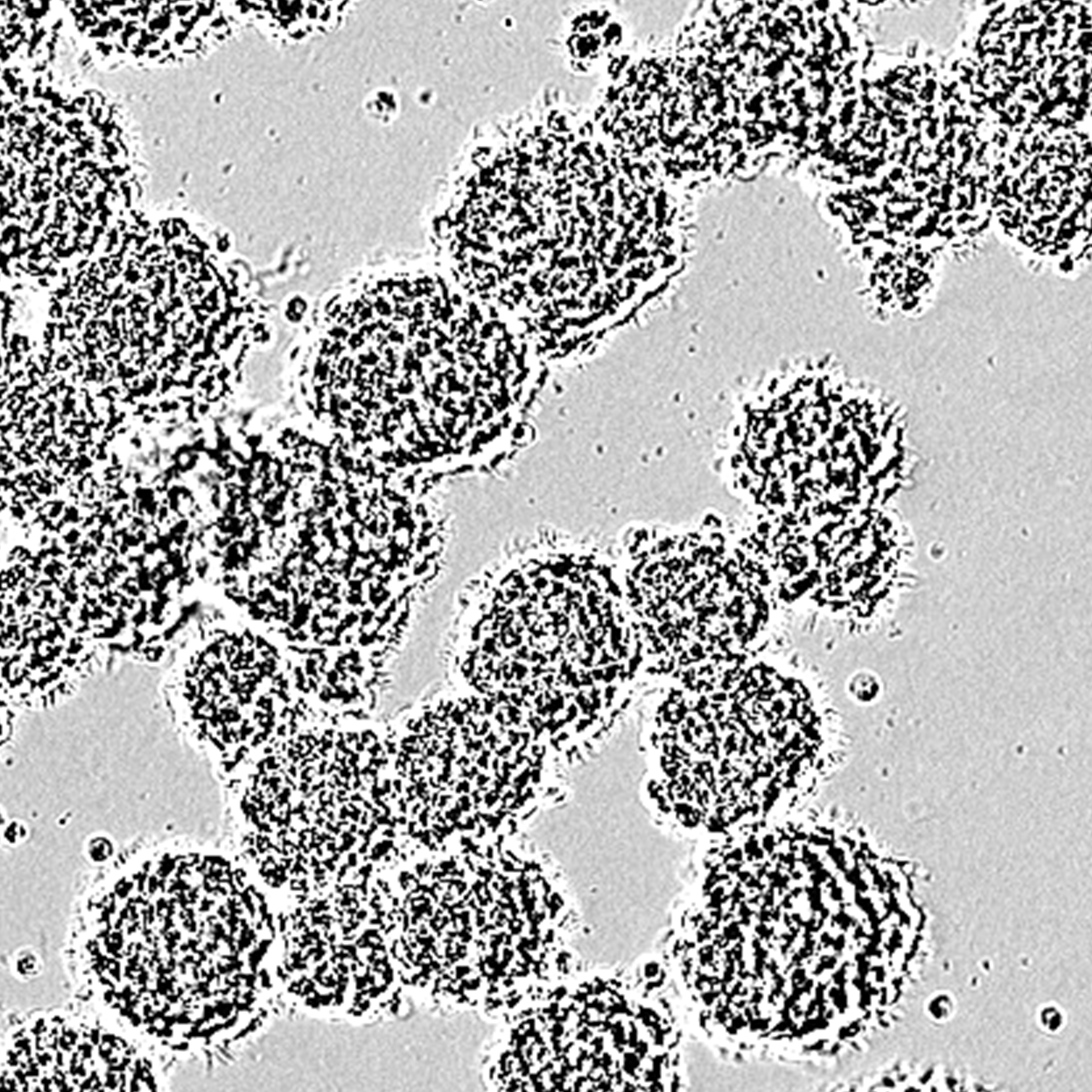

Supplement: Supplementary file 12 — Figure EV4 Source Data [file 44319_2026_759_MOESM12_ESM.zip › Source Data for Fig. EV4/Fig. EV4B/Usb1 ff;Emx1-Cre.tif]

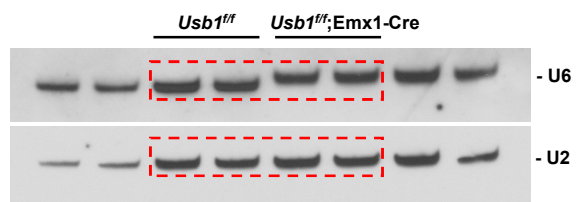

Supplement: Supplementary file 12 — Figure EV4 Source Data [file 44319_2026_759_MOESM12_ESM.zip › Source Data for Fig. EV4/Fig. EV4C/northern.pdf]
