# Supplementary material for: TUT1-catalyzed U6 snRNA 3′-end maturation is essential for RNA splicing and stem cell survival
Source: EMBO Rep. 2026 Apr 9;27(10):2703–30. doi: 10.1038/s44319-026-00759-8 (PMC13219678; doi:10.1038/s44319-026-00759-8)
Supplement: Supplementary file 13 — Expanded View Figures [file 44319_2026_759_MOESM13_ESM.pdf]

Expanded View Figures

Figure EV1. *Tut1* heterozygous mice generated by nestin-Cre (*Tut1*$^{f/+}$;nestin-Cre) are normal without behavioral abnormalities.

(**A**) The number of pups recovered from *Tut1*$^{f/+}$;nestin-Cre intercrosses. (**B**) Sagittal sections and H&E staining of P70 wild-type (WT) and *Tut1*$^{f/+}$;nestin-Cre brains ($n = 3$). Scale bar: 50 µm. (**C**) Open field test. Representative traces of WT ($n = 17$) and *Tut1*$^{f/+}$;nestin-Cre ($n = 16$) mice in the open field (left panel), and time spent in the indicated areas (right panel). The data represents mean ± SEM. *P* values, unpaired Student's *t*-test. (**D**) Tree-chamber sociability and social novelty test. Representative traces of WT ($n = 16$) and *Tut1*$^{f/+}$;nestin-Cre (Het) ($n = 15$) mice in three-chamber (left panel), and total sniffing time spent toward stranger 1 or stranger 2 (right panel). The data represents mean ± SEM. *P* values, unpaired Student's *t*-test. (**E**) Elevated plus maze test. Representative traces of WT ($n = 17$) and *Tut1*$^{f/+}$;nestin-Cre (Het) ($n = 16$) mice in the elevated plus maze (left panel), and time spent in the indicated areas (right panel). The data represent mean ± SEM. *P* values, unpaired Student's *t*-test. (**F**) Morris water maze test. Representative traces of WT ($n = 19$) and *Tut1*$^{f/+}$;nestin-Cre (Het) ($n = 20$) mice in Morris water maze (left panel), and the number of target quadrant crossings as well as time spent in the indicated areas (right panel). The data represent mean ± SEM. *P* values, unpaired Student's *t*-test. (**G**) The knockout efficiency of *Tut1*$^{f/f}$;Camk2a-Cre, determined by qPCR analysis of *Tut1* exon 2 expression as in Fig. 4C. Source data are available online for this figure.

A

Tut1$^{f/+}$;nestin-Cre X *Tut1*$^{f/+}$;nestin-Cre

Stage	Genotype		
	WT	*Tut1*$^{f/+}$;nestin-*Cre*	*Tut1*$^{f/f}$;nestin-Cre
P7	29	24	0
10 months	29	24	0

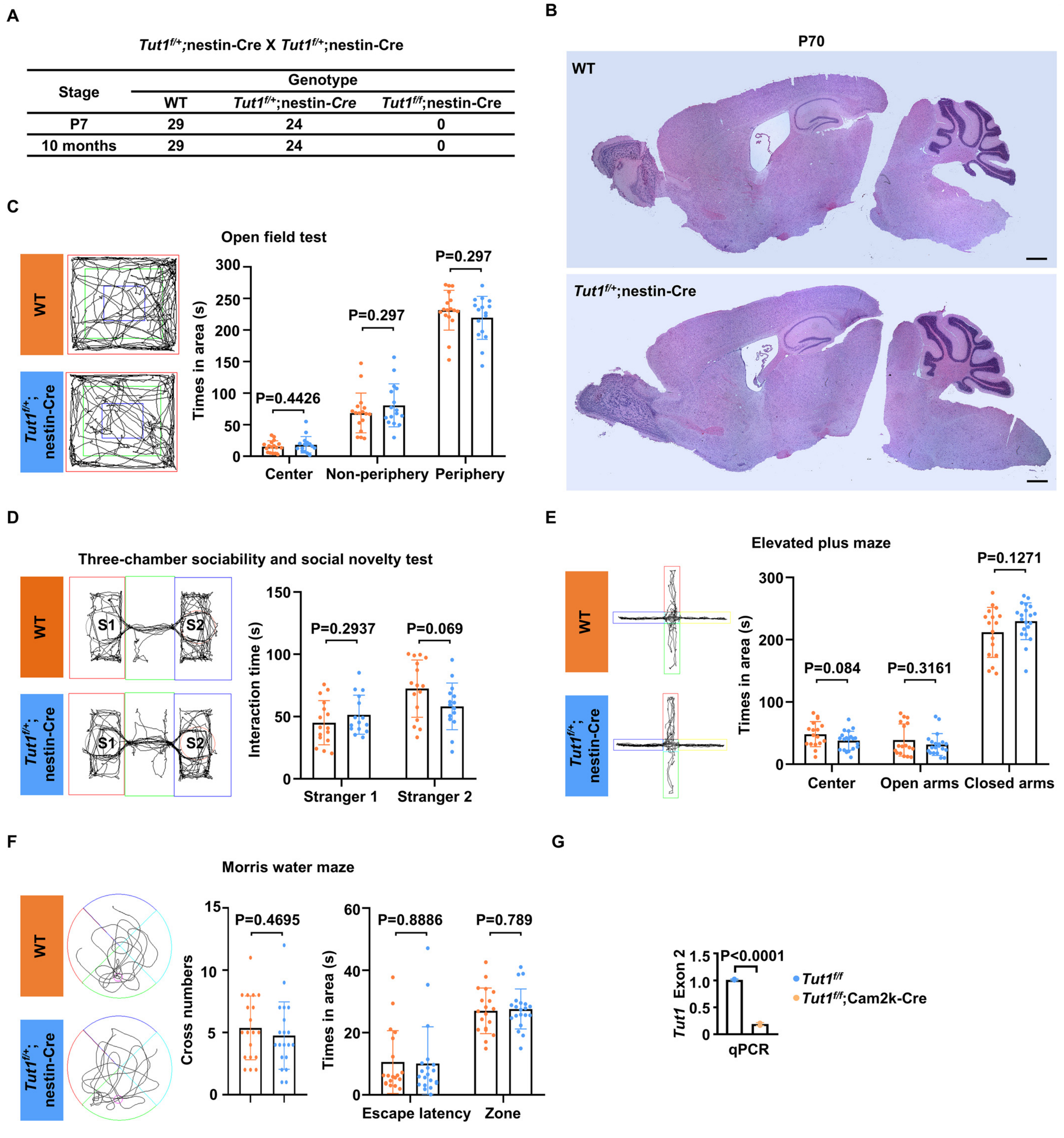

 © The Author(s)

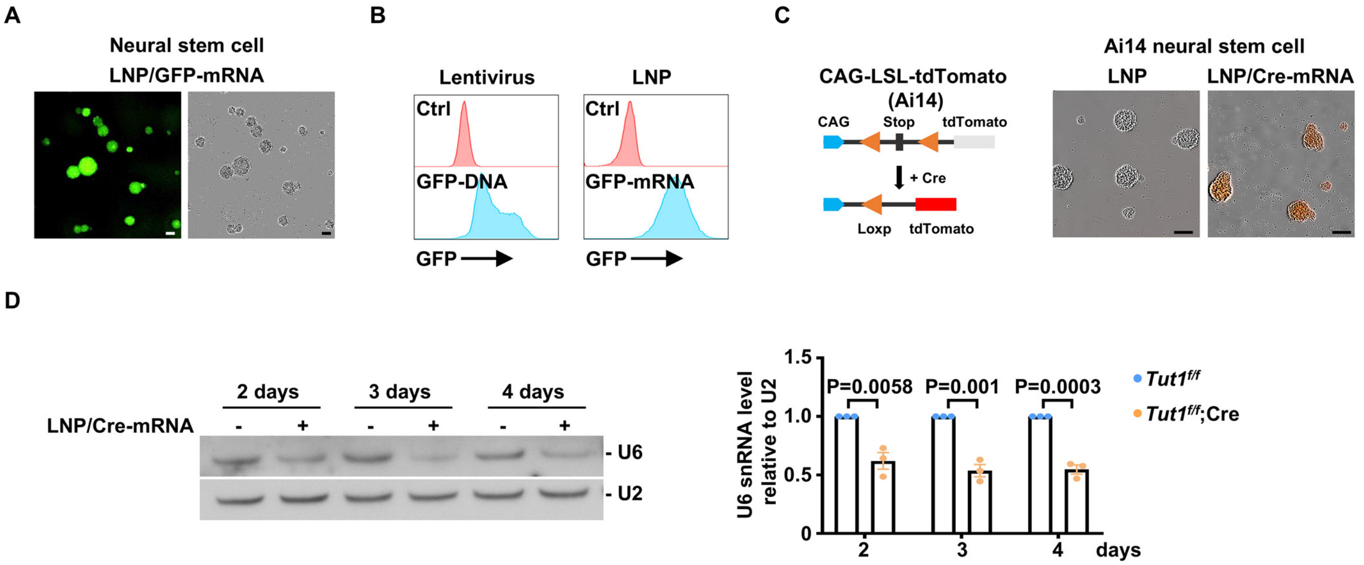

E

Sample	Data (Gb)	Total reads	% PASS	% full length	Median pass read length	Mean pass read length	No. of genes detected	% genes with AS
WT-1	12.022	26144584	33.24	88.11	623	900	13263	58.50%
WT-2	7.022	9851047	77.97	80.24	684	842	13549	56.40%
WT-3	7.343	11946731	74.21	82.99	621	737	13637	55.10%
Tut1$^{f/f}$;Cre-1	7.365	10899850	76.67	81.26	661	803	13689	55.80%
Tut1$^{f/f}$;Cre-2	10.536	16970857	72.30	77.43	632	762	13894	56.20%
Tut1$^{f/f}$;Cre-3	6.736	8966639	80.15	79.49	700	871	13555	55.80%
p53$^{f/f}$;Cre-1	8.24	12658680	76.61	81.63	651	772	13698	55.80%
p53$^{f/f}$;Cre-1	8.401	11383350	79.96	82.17	691	855	13742	56.90%
p53$^{f/f}$;Cre-1	7.597	10987475	79.62	84.05	666	801	13612	56.30%
p53$^{f/f}$;*Tut1$^{f/f}$*;Cre-1	7.393	9704333	79.20	79.15	707	893	13620	56.30%
p53$^{f/f}$;*Tut1$^{f/f}$*;Cre-1	6.934	9500305	80.38	80.95	686	841	13603	55.60%
p53$^{f/f}$;*Tut1$^{f/f}$*;Cre-1	6.311	9576746	76.89	82.41	648	778	13435	54.80%

Figure EV2. Analysis of *Tut1* function in neural stem cells in vitro.

(**A**) Incucyte live-cell imaging analysis of neural stem cells for visual inspection of overall cell morphology ($n = 3$), with or without transfection by LNP containing GFP-mRNA (LNP/GFP-mRNA),. Scale bar: 100 μm. (**B**) Lentiviral transduction of neural stem cells with GFP cDNA (left, $n = 3$) or LNP transfection with GFP-mRNA (LNP/GFP-mRNA) (right, $n = 3$). Flow cytometry was used to analyze GFP-positive cells 2 days after transduction or transfection. Notably, compared to lentiviral transduction, LNP transfection showed a higher percentage of GFP-positive cells and more uniform GFP expression. (**C**) Ai14 neural stem cells transfected with LNP/Cre-mRNA. (**D**) Northern blot analysis of U6 snRNA expression in neural stem cells after LNP/Cre-mRNA transfection. Time course analysis of U6 snRNA expression. Total RNA was derived from *Tut1$^{f/f}$* neural stem cells with or without LNP/Cre-mRNA transfection for 2, 3, or 4 days ($n = 3$). U2 snRNA was used as a loading control. (**E**) Nanopore-sequencing statistics for Fig. 4H. The percentage of full-length (% full length) reads was calculated as the number of reads covering 80% of nucleotides for the transcript. The number of genes (No. of genes detected) was computed by counting the genes, including all the isoforms. Genes with multiple isoforms identified were considered alternatively spliced (% genes with AS). Source data are available online for this figure.

© The Author(s)

Sample	Data (Gb)	Total reads	% PASS	% full length	Median pass read length	Mean pass read length	No. of genes detected	% genes with AS
Tut1$^{f/f}$-1	1.606	7436242	96.72	54.83	728	1068	12442	61.50%
Tut1$^{f/f}$-2	1.69	7886748	97.19	56.50	687	1011	12788	61.30%
Tut1$^{f/f}$;Emx1-Cre-1	1.712	7903509	96.91	56.28	696	1039	13158	61.50%
Tut1$^{f/f}$;Emx1-Cre-2	1.247	5850069	97.24	61.85	662	975	12522	60.30%

Figure EV3. Nanopore-sequencing statistics for Fig. 5.

The percentage of full-length (% full length) reads was calculated as the number of reads covering 80% of nucleotides for the transcript. The number of genes (No. of genes detected) was computed by counting the genes, including all the isoforms. Genes with multiple isoforms identified were considered alternatively spliced (% genes with AS).

 © The Author(s)

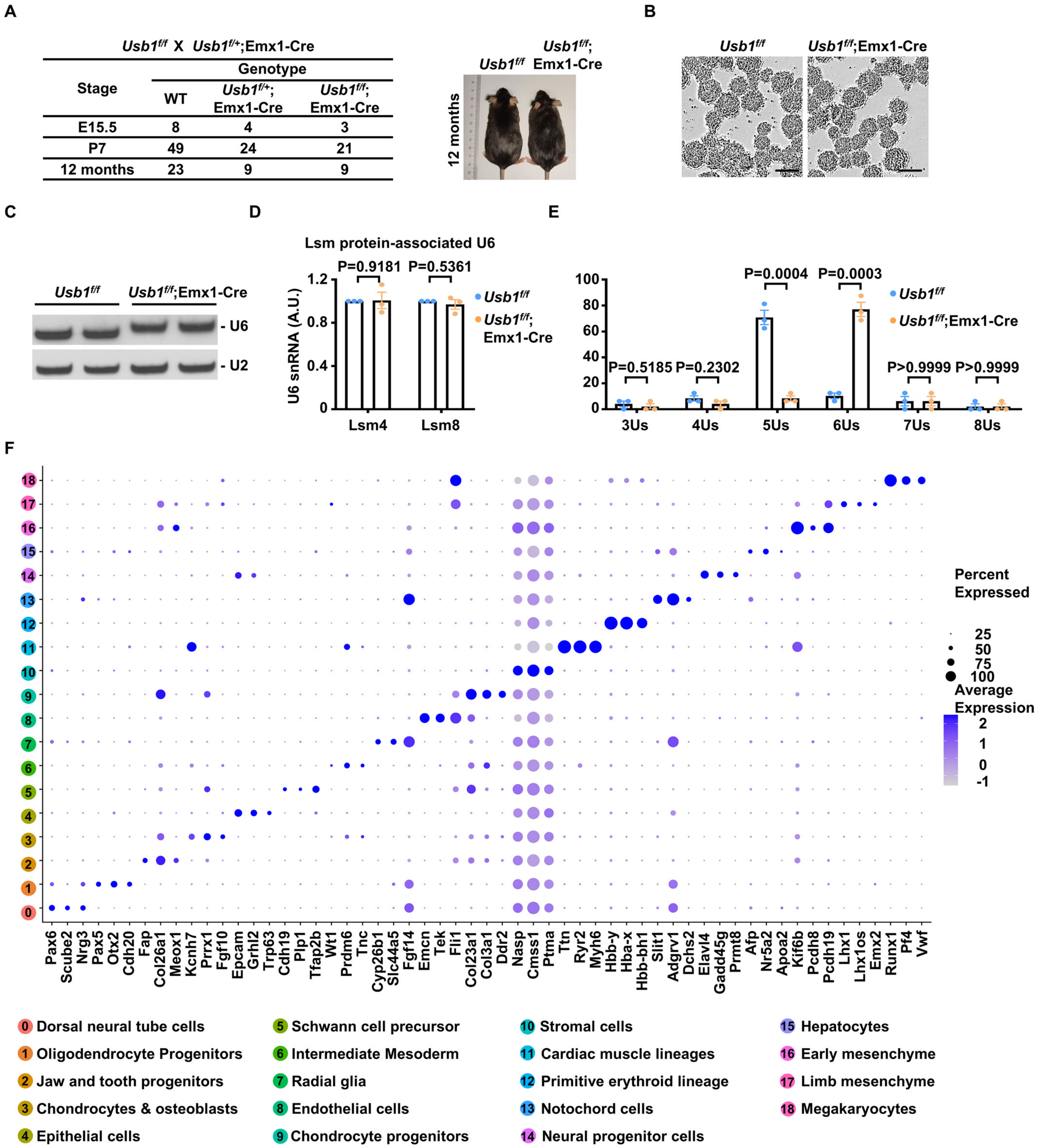
A
Usb1f/f X Usb1f/+;Emx1-Cre
Stage
Genotype
WT
Usb1f/+; Emx1-Cre
Usb1f/f; Emx1-Cre
E15.5 8 4 3
P7 49 24 21
12 months 23 9 9
12 months
Usb1f/f
Usb1f/f; Emx1-Cre
B
Usb1f/f
Usb1f/f;Emx1-Cre
C
Usb1f/f
Usb1f/f;Emx1-Cre
- U6
- U2
D
Lsm protein-associated U6
P=0.9181 P=0.5361
U6 snRNA (A.U.)
1.2
0.8
0.4
0
Lsm4
Lsm8
Usb1f/f
Usb1f/f; Emx1-Cre
E
100
80
60
40
20
0
P=0.5185 P=0.2302 P=0.0004 P=0.0003 P>0.9999 P>0.9999
3Us 4Us 5Us 6Us 7Us 8Us
Usb1f/f
Usb1f/f;Emx1-Cre
F
18 17 16 15 14 13 12 11 10 9 8 7 6 5 4 3 2 1 0
Pax6 Scube2 Nrg3 Pax5 Otx2 Cdh20 Fap Col26a1 Meox1 Kcnh7 Prrx1 Fgf10 Epcam Grhl2 Trp63 Cdh19 Plp1 Tfap2b Wt1 Prdm6 Tnc Cyp26b1 Slc44a5 Fgf14 Emcn Tek Fli1 Col23a1 Col3a1 Ddr2 Nasp Cmss1 Ptma Ttn Ryr2 Myh6 Hbb-y Hba-x Hbb-bh1 Slit1 Adgrv1 Dchs2 Elavl4 Gadd45g Prmt8 Afp Nr5a2 Apoa2 Kif6b Pcdh8 Pcdh19 Lhx1 Lhx1os Emx2 Runx1 Pf4 Vwf
Percent Expressed
25
50
75
100
Average Expression
2
1
0
-1
0 Dorsal neural tube cells
1 Oligodendrocyte Progenitors
2 Jaw and tooth progenitors
3 Chondrocytes & osteoblasts
4 Epithelial cells
5 Schwann cell precursor
6 Intermediate Mesoderm
7 Radial glia
8 Endothelial cells
9 Chondrocyte progenitors
10 Stromal cells
11 Cardiac muscle lineages
12 Primitive erythroid lineage
13 Notochord cells
14 Neural progenitor cells
15 Hepatocytes
16 Early mesenchyme
17 Limb mesenchyme
18 Megakaryocytes

Figure EV4. Analysis of *Usb1* function by germline and conditional knockout mouse models.

(**A**) Genotype analyses of pups generated from crossing of *Usb1*$^{f/+}$;Emx1-Cre and *Usb1*$^{f/f}$ mice. Wild-type (WT) denotes both *Usb1*$^{f/+}$ and *Usb1*$^{f/f}$ genotypes. Littermates of *Usb1*$^{f/f}$ and *Usb1*$^{f/+}$;Emx1-Cre mice at the age of 12 months were shown ($n = 21$). (**B**) Phase contrast images of cultured neural stem cells derived from E12.5 wild-type and *Usb1*$^{f/f}$;Emx1-Cre neocortices ($n = 3$), respectively. Scale bars: 100 µm. (**C**) RNA blot analysis of U6 snRNA. Total RNA was extracted from wild-type (WT) and *Usb1* knockout (*Usb1*$^{f/f}$;Emx1-Cre) neural stem cells ($n = 3$). U2 snRNA was used as a loading control. Of note, the length of U6 snRNA was extended in *Usb1*$^{f/f}$;Emx1-Cre cells. (**D**)The interaction between U6 snRNA and Lsm proteins in *Usb1*$^{f/f}$;Emx1-Cre neural stem cells. Lsm4- or Lsm8-associated U6 snRNA was quantified by qPCR. The data represent mean ± SEM. AU arbitrary unit. (**E**) 3′ RACE analysis of U6 oligo(U) tails in wild-type (*Usb1*$^{f/f}$) and *Usb1* knockout (*Usb1*$^{f/f}$;Emx1-Cre) neural stem cells ($n = 3$). The data represent mean ± SEM. *P* values, unpaired Student's *t*-test. (**F**) Dot plot showing the expression of three marker genes for each cell type. The size of the dot denotes the percentage of cells expressing the indicated genes, and the color of the dot denotes the average expression level. Source data are available online for this figure.

 © The Author(s)
